# Metabolic labelling of DNA in cells by means of the “photoclick” reaction triggered by visible light†

**DOI:** 10.1039/d3cb00150d

**Published:** 2023-08-30

**Authors:** Lisa Rieger, Bastian Pfeuffer, Hans-Achim Wagenknecht

**Affiliations:** a Institute of Organic Chemistry, Karlsruhe Institute of Technology (KIT), Fritz-Haber-Weg 6 Karlsruhe 76131 Germany Wagenknecht@kit.edu

## Abstract

Two pyrene-tetrazole conjugates were synthesized as photoreactive chromophores that allow for the first time the combination of metabolic labelling of DNA in cells and subsequent bioorthogonal “photoclick” modification triggered by visible light. Two strained alkenes and three alkene-modified nucleosides were used as reactive counterparts and revealed no major differences in their “photoclick” reactivity. This is a significant advantage because it allows 5-vinyl-2′-deoxyuridine to be applied as the smallest possible alkene-modified nucleoside for metabolic labelling of DNA in cells. Both pyrene-tetrazole conjugates show fluorogenicity during the “photoclick” reactions, which is a second advantage for cellular imaging. Living HeLa cells were incubated with 5-vinyl-2′-deoxyuridine for 48 h to ensure one cell division. After fixation, the newly synthesized genomic DNA was successfully labelled by irradiation with visible light at 405 nm and 450 nm. This method is an attractive tool for the visualization of genomic DNA in cells with full spatiotemporal control by the use of visible light as a reaction trigger.

## Abstract 2

Two pyrene-tetrazole conjugates were synthesized as photoreactive chromophores that allow for the first time the combination of metabolic labelling of DNA in cells and subsequent bioorthogonal “photoclick” modification triggered by visible light.

## Graphical abstract

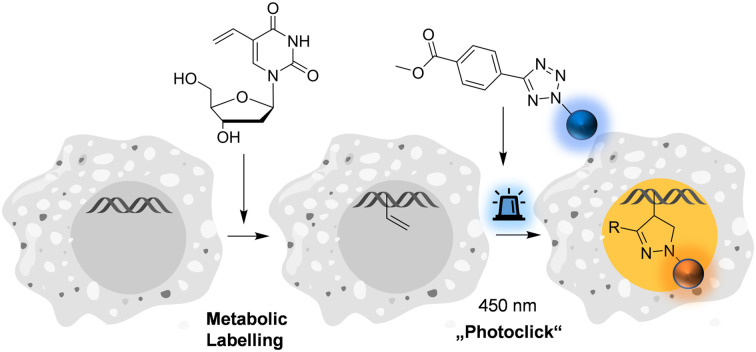

## Introduction

The fluorescent labelling of biomolecules in the cellular environment represents a prerequisite to visualize and understand biological structures and processes in living cells. Bioorthogonal modification strategies follow this research task and have emerged as a powerful tool to label proteins, glycans and nucleic acids.^1–3^ Transition metal-free bioconjugation is preferred for the labelling of nucleic acids to avoid oxidative damage and cytotoxic metals.^4^ An intriguing bioconjugation is the photoinduced reaction between diaryltetrazoles and alkenes, because it combines high reaction rates with control by light *via* intensity and wavelength.^3^ The reaction was reported already in the 1960s by Huisgen *et al.* as the 1,3-dipolar cycloaddition of an intermediate diarylnitrilimine that was generated from tetrazoles after releasing N_2_.^5^ The reaction with alkenes forms pyrazolines, and is called “photoclick” reaction for bioconjugations^3,6^ or “nitrile-imine mediated tetrazole-ene cycloaddition” for polymers.^7^

**Fig. 1:**
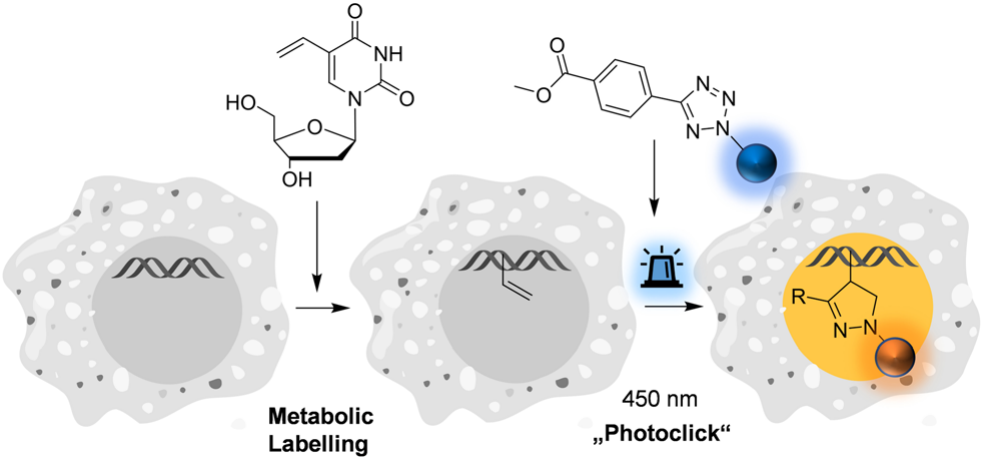
Metabolic labelling of DNA using 5-vinyl-2′-deoxyuridine and pyrene-modified tetrazoles (for structures see Fig. 2) by means of the fluorogenic “photoclick” reaction triggered with visible light.

Diaryltetrazoles are typically irradiated by UV-B light (280–315 nm). Their absorbance was red-shifted by substituents, but only to the border of the visible light (for one-photon excitations),^8^ by Lin *et al.* to 405 nm and by Barner-Kowollik *et al.* to 410 nm, the latter only in the context of polymer chemistry.^9,10^ The first “photoclick” labellings for DNA and RNA were established by us with tetrazole-modified nucleotides.^11^ The use of UV light has several principal disadvantages, including safety hazards, cell damage, cell toxicity, and missing chemical selectivity (in the UV range many biocompounds are excited). Shifting the irradiation wavelength into the visible light range would require even larger diaryltetrazoles as DNA modifications, which are very likely not compatible with metabolic labelling^12,13^ as a new technique for DNA labelling in cells.^14^ Therefore, it looked reasonable to use the complementary approach and apply tetrazoles as reactive units at the fluorophore together with alkene-modified DNA for “photoclick” labellings. Herein we follow this new approach and present the pyrene-modified tetrazoles 1 and 2 and their reactivity with a small library of alkenes and alkynes (Fig. 1). This bioconjugation is suitable in particular for DNA as it combines high reactivity induced by visible light with fluorogenicity for metabolic labelling in cells. There are reports on photoactivatable fluorophores^13^ and photocages^14^ to control the reactions by light, but to the best of our knowledge, visible light^15^ has not yet been used for the metabolic labelling of nucleic acids. This is disappointing because the use of light has the intrinsic property of spatiotemporal resolution.^16,17^

## Results and discussion

**Fig. 2:**
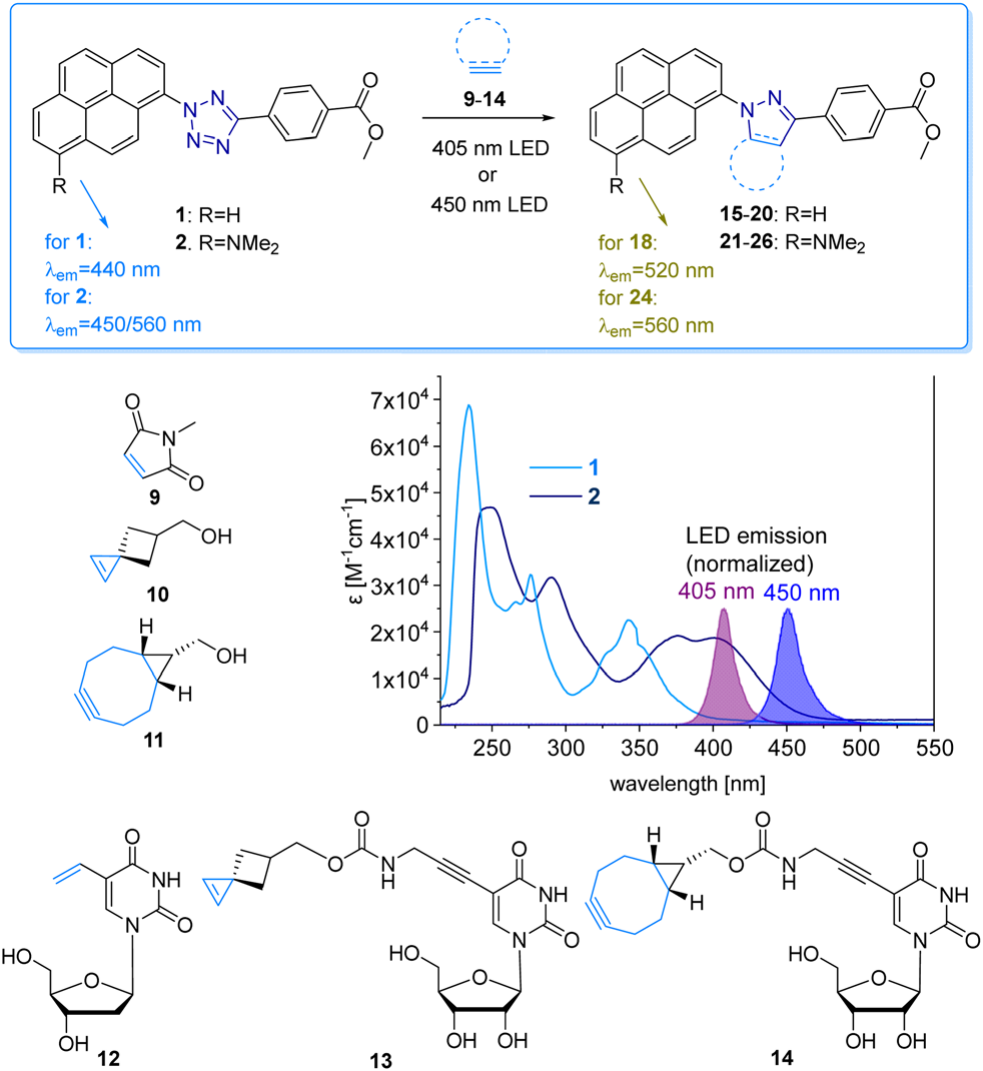
“Photoclick” reaction between pyrene tetrazoles 1 and 2 with alkenes 9, 10 and alkyne 11, and alkene/alkyne-modified nucleosides 12-14 to products 15-20 and 21-26, respectively. UV/Vis extinction of 1 and 2 in comparison with the normalized emission of the 405 nm and 450 nm LEDs.

The pyrene tetrazole 1 and the *N*,*N*-dimethylaminopyrene tetrazole 2 (Fig. 2) were prepared to allow the “photoclick” modification of DNA to be triggered by light in the visible range (*λ*_exc_ = 405–450 nm). In both molecules, the pyrene moiety was attached to the nitrogen in position 1. The synthesis is straightforward and applies the tetrazole formation from the pyrene diazonium salts and the hydrazone sulfonate as the central reaction (ESI,† Schemes S1 and S2, and Fig. S1–S9).^18^ In order to identify the appropriate LEDs for the irradiation experiments, both synthetic tetrazoles 1 and 2 were characterized by their UV/Vis absorption (Fig. 2). The tetrazoles show a significant bathochromic shift not only just across the border into the visible range (for 1) but clearly into the visible range (for 2). The absorbance of tetrazole 1 shows a local maximum at 342 nm with an extinction coefficient of *ε*_342 nm_ = 2.3 × 10^4^ M^−1^ cm^−1^. The broad shoulder of the absorbance into the visible range indicates that tetrazole 1 can be activated by light of a 405 nm LED. Tetrazole 2 shows a strong bathochromic shift with two local maxima at 375 nm and 405 nm, and an extinction coefficient of *ε*_405 nm_ = 1.9 × 10^4^ M^−1^ cm^−1^. Accordingly, tetrazole 2 can be excited by irradiation with a 450 nm LED, which is clearly in the visible light range and should therefore be optimal for cellular applications.

**Fig. 3:**
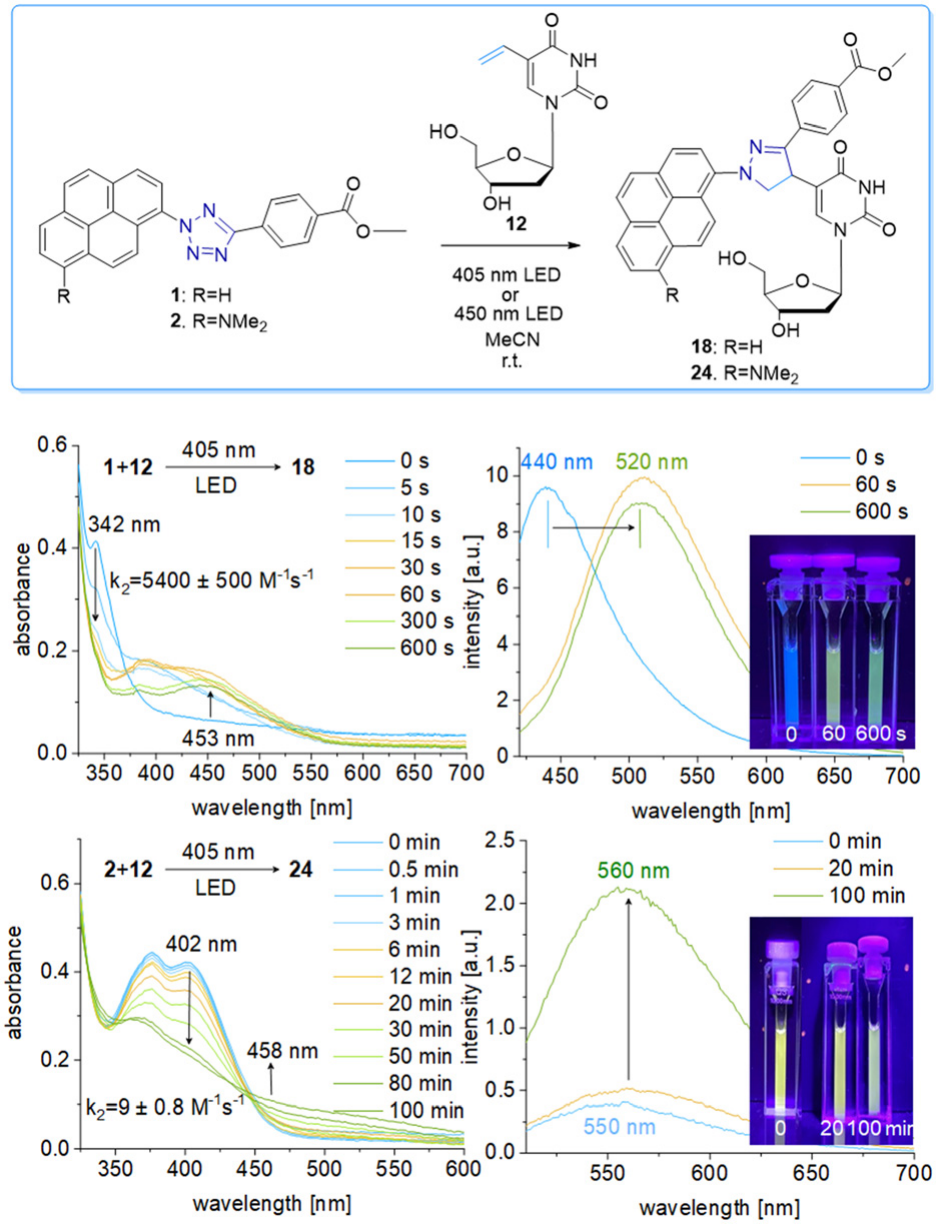
Top: “Photoclick” reaction of tetrazole 1 (25 μM) or tetrazole 2 (25 μM), respectively, and nucleoside 12 (250 μM) to product 18 or 24 in MeCN by irradiation using the 405 nm or 450 nm LED. Middle: UV/Vis absorption changes, fluorescence changes (*λ*_exc_ = 480 nm) and images of the cuvettes for the reaction of tetrazole 1 with nucleoside 12 at different irradiation times. Bottom: The corresponding results of the “photoclick” reaction of tetrazole 2 and nucleoside 12.

We chose a small library of reactive counterparts, including maleimide 9 as an unstrained alkene, spiro[2.3]hex-1-ene (SPH) 10 as a strained alkene, and bicyclo[0.1.6]nonyne (BCN) 11 as a strained alkyne, and the corresponding modified nucleosides, including 5-vinyl-2′-deoxyuridine (12), the SPH-modified uridine 13 and the BCN-modified uridine 14, to evaluate the optimal kinetics by their reaction rates (Table 1, for synthesis of 10-14 see Scheme S3 and Fig. S10–S30, ESI†). We assume that an influence of the additional 2′-hydroxyl group in 13 and 14 on the “photoclick” reactivity can be excluded. We representatively show the UV/Vis analysis of the “photoclick” reaction of 1 and 2 (Fig. 2) with 5-vinyl-2′-deoxyuridine (12) to provide details about the changes of absorbance and fluorescence properties. These photoclick reactions *in vitro* were performed in MeCN and irradiated with a 405 nm LED to allow their comparison (Fig. 3 middle). The results of the other “photoclick” reactions with 9-11, 13 and 14 are provided in the ESI† (Schemes S4–S15 and Fig. S31–S86). During the photoinduced reaction of tetrazole 1, the characteristic maximum at *λ* = 342 nm vanishes and shifts bathochromically to 400 nm. According to the isosbestic point observed at 375 nm, the reaction seems to be completed already after 60 s without any intermediate. This shows that the nitrilimine as an intermediate after the photolysis of tetrazole 1 is extremely short-lived and rapidly reacts with the provided alkene in 12, which will be further evidenced by the kinetic analysis (*vide infra*). In the time range between 60 s and 600 s the isosbestic points vanish and the absorbance shifts further bathochromically indicating a second reaction. Control experiments with irradiation times of only 60 s suggest that this second step might also be a light-induced reaction as no bathochromic shift is observed after the irradiation (Fig. S40, ESI†). According to mass spectrometry analysis (Fig. S44, ESI†), we assume that the second step is a light-induced dehydrogenation of the pyrazoline to the pyrazole chromophore in the final product 18. During the course of the reaction the red-shift of the fluorescence from 440 nm (starting material) to 520 nm (product) was observed, which could be visualized in the cuvettes as a color change from blue to yellow-green. Excitation spectra (Fig. S45, ESI†) and 3D fluorescence (Fig. S46, ESI†) reveal that the pyrene is the emitter for the 440 nm fluorescence in the tetrazole 1 as the starting material (excited at 320–360 nm), whereas the fluorescence of the product 18 originates from the pyrazole chromophore (excited at 380–420 nm). In contrast to tetrazole 1, the reaction of tetrazole 2 with 12 shows an absorbance loss of the characteristic bands at 376 nm and 402 nm together with an isosbestic point at 450 nm (Fig. 3 bottom). When the fluorescence is excited above that isosbestic point at 480 nm, the “photoclick” reaction is fluorogenic because the product 24 is fluorescent with a broad band at 560 nm (Fig. S65b, ESI†). Additionally, the emission color changes slightly from yellow (excitation at 405 nm) to green (excited at 480 nm). Taken together, the fluorescence color change and fluorogenicity observed for the reactions of tetrazole 1 and tetrazole 2 are significant advantages for the imaging of metabolically labelled genomic DNA in cells (Fig. 3).

**Table 1:** Second order rate constants *k*_2_ [M^−1^ s^−1^] of the “photoclick” reactions between tetrazoles 1 and 2, and alkenes/alkynes 9-14 to products 15-20 and 21-26

Alkene/alkyne	Tetrazole 1		Tetrazole 2
	Photolysis *k*_2_ [M^−1^ s^−1^]	Product formation *k*_2_ [M^−1^ s^−1^]	Photolysis *k*_2_ [M^−1^ s^−1^]
—	3640 ± 250^a^	—	15 ± 1.6^a^
9	6320 ± 950^a^	15 : 650 ± 20^b^	30 ± 3^a^
	600 ± 25^b^		2.0 ± 1.1^b^
10	2730 ± 130^a^	16 : 540 ± 150^b^	25 ± 3^a^
	560 ± 170^b^		1.6 ± 1^b^
11	4070 ± 190^a^	17 : 680 ± 190^b^	27 ± 4^a^
	480 ± 110^b^		7.0 ± 0.8^b^
12	5400 ± 500^a^	n.d.^c^	9.0 ± 0.8^a^
13	5000 ± 390^a^	n.d.^c^	11 ± 0.8^a^
14	6800 ± 700^a^	n.d.^c^	15 ± 1^a^

a Determined by means of UV/Vis absorption changes with 1 or 2 (each 25 μM) and 9-14 (each 250 μM).

b Determined by means of RP-HPLC analyses with 1 or 2 (each 100 μM) and 9-14 (each 1000 μM).

c Not determined due to insufficient HPLC separation.

To gain kinetic information about the photoinduced reactivity of 1 and 2, the library of different reactive counterparts 9-14 was investigated. These results will help to decide which particular reaction is the best for application in cells. The “photoclick” reaction is a two-step reaction: the photolysis of the tetrazole forms the nitrilimine in the first step, and this highly reactive intermediate reacts with the alkene or alkyne in the second step. The determination of the second order rate constant was firstly based on the absorbance loss at *λ* = 342 nm for 1 and *λ* = 402 nm for 2 which correspond to the photolysis of the tetrazoles. This investigation by optical spectroscopy allows only a rather low concentration of tetrazole 1 and 2 (25 μM); a tenfold access of the reactive counterpart was applied (250 μM) to yield pseudo-first order kinetics according to the literature.^19^ The measured rate constants *k*_2_ for the photolysis with tetrazole 1 (when excited by the 405 nm LED) are likewise and lie in the range between 2730 ± 130 M^−1^ s^−1^ to 6800 ± 700 M^−1^ s^−1^ (Table 1). A quite similar behavior was observed for the “photoclick” reactions with tetrazole 2 (also excited at 450 nm LED to allow the direct comparison), albeit the photolysis proceed much slower with rate constants *k*_2_ in the range between 9 ± 0.8 M^−1^ s^−1^ and 30 ± 3 M^−1^ s^−1^. Here also, only little variations of the rate constants *k*_2_ were observed. The absorbance changes of the control experiments with 1 and 2 without the addition of any alkene or alkyne as reactive counterpart gave similar results, with rate constants for 1 of *k*_2_ = 3640 ± 250 M^−1^ s^−1^ and for 2 of *k*_2_ = 15 ± 1.6 M^−1^ s^−1^ (Fig. S87 and S88, ESI†). The rate constants for the photolysis were verified by means of RP-HPLC analyses in fourfold higher concentrations of the reacting partners (100 μM 1 or 2) while keeping the tenfold excess of the reactive counterpart (1 mM) allowing also to determine the rate constants for the “photoclick” reactions based on the product formation. This was done for the reactions of tetrazole 1 with the reactive functional groups 9-11 (Fig. S89, S90, S92, S93, S95 and S96, ESI†). The resulting second-order rate constants for the photolysis, which correspond to the loss of starting material in the HPLC analytics, are generally lower than those obtained from the UV/Vis absorption analyses, with *k*_2_ = 600 ± 25 M^−1^ s^−1^ for the photolysis of 1 in the presence of 9, *k*_2_ = 560 ± 170 M^−1^ s^−1^ in the presence of 10, and *k*_2_ = 480 ± 110 M^−1^ s^−1^ in the presence of 11. The rates from these experiments are lower than those from the UV/Vis absorption changes, which is probably due to light filtering effects caused by the higher concentrations of both the tetrazoles and the reactive counterparts 9-11. Similar experiments with the nucleosides 12-14 failed due to insufficient HPLC separation.

The rate constants for the product formation from tetrazole 1 according to HPLC analyses are *k*_2_ = 650 ± 20 M^−1^ s^−1^ for the reaction with 9 to product 15, *k*_2_ = 540 ± 150 M^−1^ s^−1^ for the reaction with 10 to product 16, and *k*_2_ = 680 ± 190 M^−1^ s^−1^ for the reaction with 11 to product 17. All three products were confirmed by ESI-HRMS (Fig. S91, S94 and S97, ESI†). The spirocycle 10 and the bicyclononye 11 are representatives of highly strained alkenes and alkynes, respectively, and should therefore be very reactive. In particular, the spirocycle 10 and the bicyclononyne 11 were identified to promote extremely fast “photoclick” reactions.^19,20^ Obviously, the rates of these “photoclick” reactions with tetrazole 1 do not depend on the reactive counterpart. This stands in contrast to the aforementioned results by *Lin et al*. who showed that the SPH moiety of 10 speeds up the reaction kinetics up to *k*_2_ = 39 000 M^−1^ s^−1^.^19^ Compared to those, the “photoclick” reactions with tetrazole 1 and 2 are not only slower, but also the alkene ring strain obviously does not significantly matter. The rate for the photolysis is – within the experimental error - at least similar or even identical to the rate for product formation, not only for each of the reactions but also in comparison between them. This indicates that it might be a question of the light source and the light intensity in combination with the inner filter effect of both the tetrazoles and the reacting counterparts that determine the kinetics. The photolysis step determines the kinetics of the whole “photoclick” reaction. This is also supported by the major difference between both tetrazoles, as the rate constants for the photolysis with 2 are two order of magnitudes lower than those of 1, when excited at 450 nm, and lie in the range between 1.6 ± 1 and 7 ± 0.8 M^−1^ s^−1^. As a consequence, for “photoclick” labelling in cells, the simplest and smallest alkene-modified nucleoside, which is 12, can be used as a reactive counterpart in metabolically modified DNA. It must be noted that the efficiency of metabolic labelling strongly depends on the size of the modification as part of the applied modified 2′-deoxynucleoside.^13^ The nucleoside 12 has a similar size as the natural thymidine and is therefore well accepted by the cellular enzymes to be metabolically incorporated into DNA. Based on our kinetic analysis, it is not necessary to increase the efforts to metabolically label genomic DNA with the nucleosides 13 or 14, carrying large modifications. Additionally, the fluorogenic properties of the tetrazoles 1 and 2 are advantageous compared to other literature-known tetrazoles because the method allows the combination of the visible light-induced “photoclick” reaction with metabolic labelling of genomic DNA in HeLa cells.

**Fig. 4:**
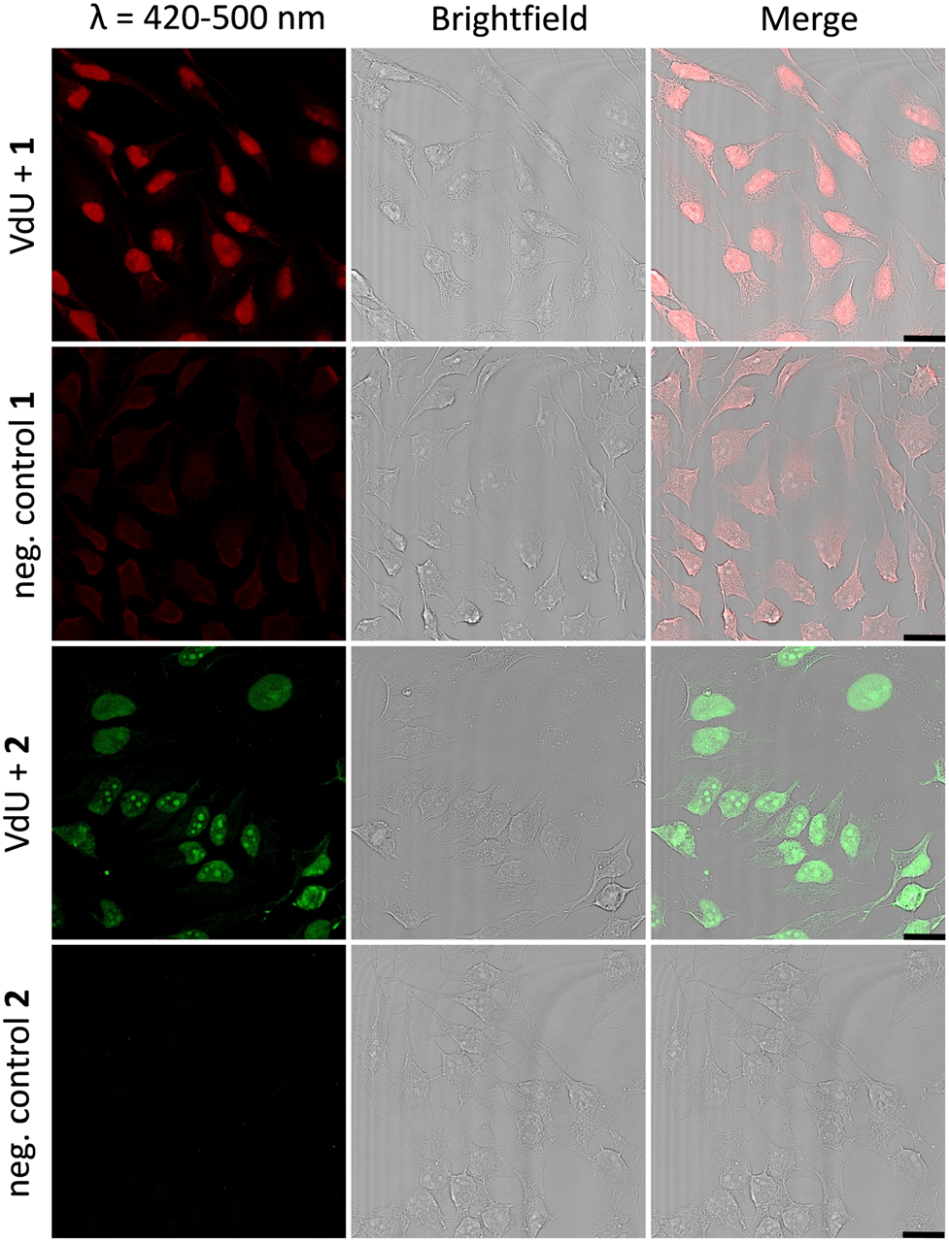
HeLa cells metabolically labelled with 12 (20 μM) for 48 h, fixed, denatured with HCl, treated with 1 (60 μM) or 2 (30 μM), and subsequently photoirradiated with a 405 nm LED for 90 s (1)/20 min (2), respectively. Imaging was performed with confocal fluorescence microscopy with a 405 nm laser and a fluorescence emission channel at 420–500 nm. The results are always shown with an additional brightfield and merged picture. As negative controls 1 and 2, cells were only treated with 1 or 2, respectively, without prior incubation with 12. Scale bar: 20 μm. The different concentrations of 1 (60 μM) and 2 (30 μM) are for technical reasons to obtain the best cell images.

Fixed HeLa cells were labelled using both tetrazoles by means of visible light at 405 nm and 450 nm. The resulting fluorescence allows the imaging of genomic DNA in cells by confocal microscopy with full spatiotemporal control (Fig. 4). HeLa cells were incubated with 12 (20 μM) for 48 h to ensure at least one cell division. After the metabolic labelling of the nascent DNA by 12, cells were fixed with PFA (4%) and the DNA denaturated with HCl. As a next step, the cells were treated with 1 (60 μM) or 2 (30 μM) for 1 h and subsequently photoirradiated using a 405 nm LED for 90 s (1) and 20 min (2), respectively. After two final washing steps with MeCN, the samples were imaged *via* confocal fluorescence microscopy. Using a 405 nm laser for excitation of the fluorescence and the emission channel set to 420–500 nm, we could clearly visualize the “photoclicked” 1 and 2 mainly located in the nucleus (Fig. 4). To exclude unspecific binding and reaction of the tetrazoles, we performed the same experiments without previous metabolic labelling by excluding 12 as a negative control. In contrast, these images showed only a diffuse fluorescence pattern over the whole cell enabling us a definite distinction between unspecifically (in the absence of DNA metabolically labelled with 12) and specifically (in the presence of DNA metabolically labelled with 12) reacted fluorophores. The rather low unspecific fluorescence in the controls is probably due to photoreactions with nucleophiles (amines, thiols, *etc.*) in the cells which are typical for tetrazole photochemistry.

**Fig. 5:**
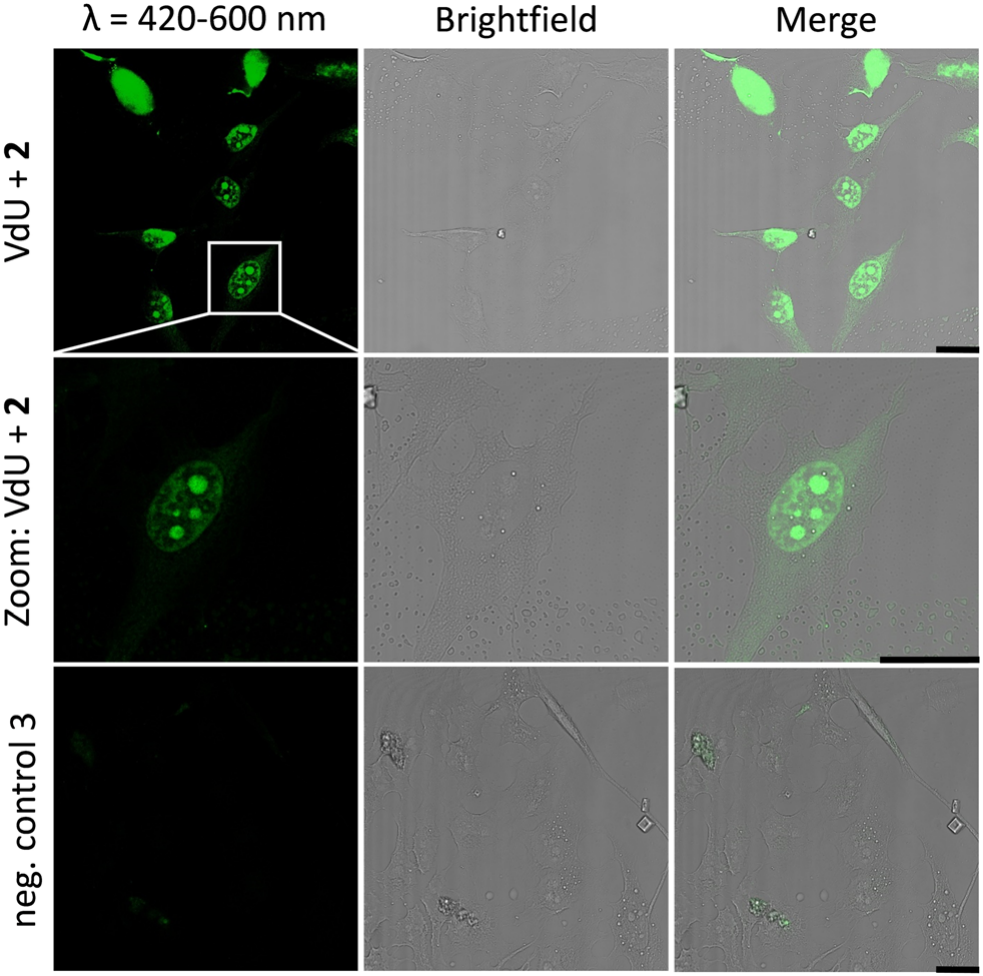
HeLa cells metabolically labelled with 12 for 48 h, fixed, denatured with HCl, treated with 2 (30 μM) and subsequently photoirradiated with a 450 nm LED for 30 min. Imaging was performed with confocal fluorescence microscopy with a 405 nm laser for fluorescence excitation and an emission channel at 420–600 nm. Results are always shown with an additional brightfield and merged picture. As a negative control, cells were only treated with 2 without prior incubation with 12. Scale bar: 20 μm.

Similar observations were made for the “photoclick” modifications with 2 using a 450 nm LED for irradiation (Fig. 5). Here the reaction products were imaged with a 405 nm laser for excitation of the fluorescence and an emission channel at 420–600 nm. With the latter results we could demonstrate the precise visualization of metabolically labelled DNA in cells by means of the “photoclick” reaction not only after irradiation by the 405 nm, but also by the 450 nm LED, which is clearly in the visible range. Through control staining experiments with DRAQ5 that stains nuclear DNA (Fig. S104 and S105, ESI†) we prove that the “photoclick” reactions enable us to image genomic DNA. The staining of the nucleoli in some cells is likely not an artifact, since fixation, permeabilization and washing precedes the imaging, and other research groups working in the field of metabolic labelling observe this type of staining also.^21^ Accordingly, the staining depends on the cell phase. It is clear that the use of visible light is not a prerequisite for labelling of fixed cells. Nevertheless, this is an important result because the application of the less harmful visible light with its intrinsic property for spatiotemporal resolution adds a huge potential for future applications in living cells, in particular with respect to the metabolic labelling strategies.

## Conclusion

We report the first example of a visible-light induced and fluorogenic “photoclick” modification of genomic DNA in cells that was previously metabolically labelled with an alkene as a reactive group. The synthesized tetrazoles 1 and 2 as photoreactive chromophores yield this unique combination. The attached pyrene not only shifts the photochemistry to the visible range but provides also the fluorescent readout for this labelling. The conversion of the tetrazole into the pyrazoline and pyrazole induces a fluorescence color change and fluorogenicity, which is a significant advantage for fluorescent cell imaging. The photolysis of the tetrazoles 1 and 2 in the presence of alkenes and alkynes as reactive counterparts for this bioorthogonal chemistry revealed no major difference in the rate constants. The determined reaction rate constants *k*_2_ for the photolysis lie in the range of 2730–6800 M^−1^ s^−1^ for 1 and 9–30 M^−1^ s^−1^ for 2. The overall rate constants based on the product formation lie in the range of 540–680 M^−1^ s^−1^ for 1. These rates do not depend on the type of alkene or alkyne as the reactive counterpart and are similar to the photolysis rates under these reaction conditions. This is an advantage of the tetrazoles 1 and 2 because the simplest and smallest alkene-modified nucleoside, which is 12, can be used as a reactive counterpart in DNA. It is known that the efficiency of metabolic labelling strongly depends on the size of the applied modified 2′-deoxynucleoside.^12,13^ The nucleoside 12 has a similar size as the natural thymidine and is therefore well accepted by the cellular enzymes to be metabolically incorporated into DNA. Fixed HeLa cells were first incubated with 12 and labelled using both tetrazoles by means of visible light at 405 nm and 450 nm. The fluorogenicity of both “photoclick” reactions allows the visualization of nascent genomic DNA in cells by confocal microscopy with full spatiotemporal control. Fluorogenicity was previously achieved by metabolic labelling using the Diels–Alder reaction with inverse electron demand in combination with an intercalating dye (acridine orange).^22^ A fluorogenic “photoclick” reaction was also described which was triggered by UV light (350 nm) and a negatively charged coumarin-tetrazole conjugate was applied although these molecules induce electrostatic repulsion with the negatively charged phosphodiester backbone of DNA.^23^ Our pyrene-tetrazole conjugates have intercalating properties (by the pyrene) and avoid electrostatic repulsion. Our results demonstrate that this method has significant potential as a versatile tag that reacts rapidly. It is clear that labelling of fixed cells could also be done by UV light instead of visible light. Nevertheless, the shift to visible light is an important result because the application of the less harmful visible light with its intrinsic property for spatiotemporal resolution adds huge potential for future applications in living cells, in particular with respect to the metabolic labelling strategies. This turns the tetrazoles into attractive tools for the visualization of genomic DNA, and also RNA, inside living cells.

## Author contributions

LR synthesized 1 and 2, performed the kinetic experiments and the cell experiments, and wrote parts of the manuscript. BP synthesized 10 and 13. HAW wrote parts of the manuscript and supervised the research.

## Conflicts of interest

There are no conflicts to declare.

## Supplementary Material

CB-004-D3CB00150D-s001
