# Supplementary material for: Metabolic labelling of DNA in cells by means of the “photoclick” reaction triggered by visible light
Source: RSC Chem Biol. 2023 Aug 30;4(12):1037–42. doi: 10.1039/d3cb00150d (PMC10685802; doi:10.1039/d3cb00150d)
Supplement: CB-004-D3CB00150D-s001 [file CB-004-D3CB00150D-s001.pdf]

Electronic Supplementary Material (ESI) for RSC Chemical Biology.
This journal is © The Royal Society of Chemistry 2023

Metabolic Labelling of DNA in Cells by Means of the "Photoclick" Reaction Triggered by Visible Light

Supporting Information

Lisa Rieger, Bastian Pfeuffer and Hans-Achim Wagenknecht*

Institute of Organic Chemistry, Karlsruhe Institute of Technology (KIT), Fritz-Haber-Weg 6, 76131 Karlsruhe, Germany

*Corresponding author. Email: Wagenknecht@kit.edu

Table of contents

1. **Materials and methods** 2
2. **Synthesis** 3
   a) **Synthesis of 1 and 2** 3
   b) **Synthesis of 12 and 13** 11
   c) **„Photoclick" reactions** 27
   d) **Irradiation of both tetrazoles without reaction partner** 54
   e) **RP-HPLC and ESI-HRMS Data** 56
   f) **Cell experiments** 65
   g) **References** 67

1. Materials and methods

All solvents and reagents were purchased from ABCR, ACROS Organics, ALFA Aesar, Carl Roth, Merck, Sigma Aldrich, VWR or Tokyo Chemical Industry (TCI). Unless stated otherwise, the chemicals were used as received. HPLC grade solvents were bought at Fisher Scientific, NMR solvents (deuterated) were acquired at Eurisotop. Reactions under argon atmosphere were heated with a heat gun, dried in high vacuum and flooded with argon (99.999 % purity). Reactions were observed via thin layer chromatography. Thin layer chromatography was performed using Fluka silica gel 60 F_{254} coated aluminum foil. Flash chromatography was performed on silica gel 60, which was purchased at Sigma Aldrich (43-60 µm).

Spectroscopy

NMR spectroscopy

NMR spectroscopic data was recorded using the following spectrometer hardware: Bruker Avance Neo ^{1}H-NMR (400 MHz), ^{13}C-NMR (101 MHz). 5-10 mg of the compound was dissolved in ca. 400 µL deuterated solvent ($CDCl_3$, DMSO, MeOD). Chemical shifts of the ^{1}H- and ^{13}C-NMR spectra are reported in parts per million (ppm) relative to the solvent as an internal standard and was converted to the TMS reference system, Routine ^{13}C-NMR spectroscopy was recorded while applying broadband ^{1}H-decoupling. Coupling constants (*J*) are given in Hertz (Hz) and the multiplicity of signals are reported as followed: s (singlet), d (doublet), t (triplet), q (quadruplet), m (multiplet), dd (doublet of doublets), dt (doublet of triplets), td (triplet of doublets), tt (triplet of triplets) and tq (triplet of quadruplets). 2D technique experiments as COSY, HSQC and HMBC were used to determine the structures of the molecules.

Mass spectrometry

High resolution mass spectrometry was performed using an electron impact ionization source on a *Q Exactive Plus Orbitrap* from *Thermo Scientific.*

Irradiation Experiments

Irradiation of the photochemical reaction was carried out using a setup which was designed and manufactured by the University of Regensburg and the workshop of the Institute for Physical Chemistry at KIT. LEDs of the following type were used to irradiate the samples. 405 nm irradiation was performed using a *Nichia NVSU233A* LED. The photoclick-reactions were irradiated from the bottom. The temperature during the reaction time was controlled by using a *LAUDA Alpha R8* thermostat at 25 °C.

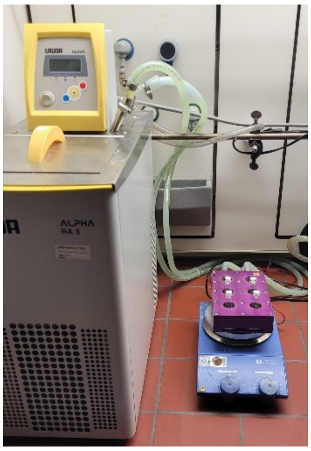

Figure S1: Irradiation set-up with thermostat, magnetic stirrer and vial rack.

2. Synthesis

a) Synthesis of 1 and 2

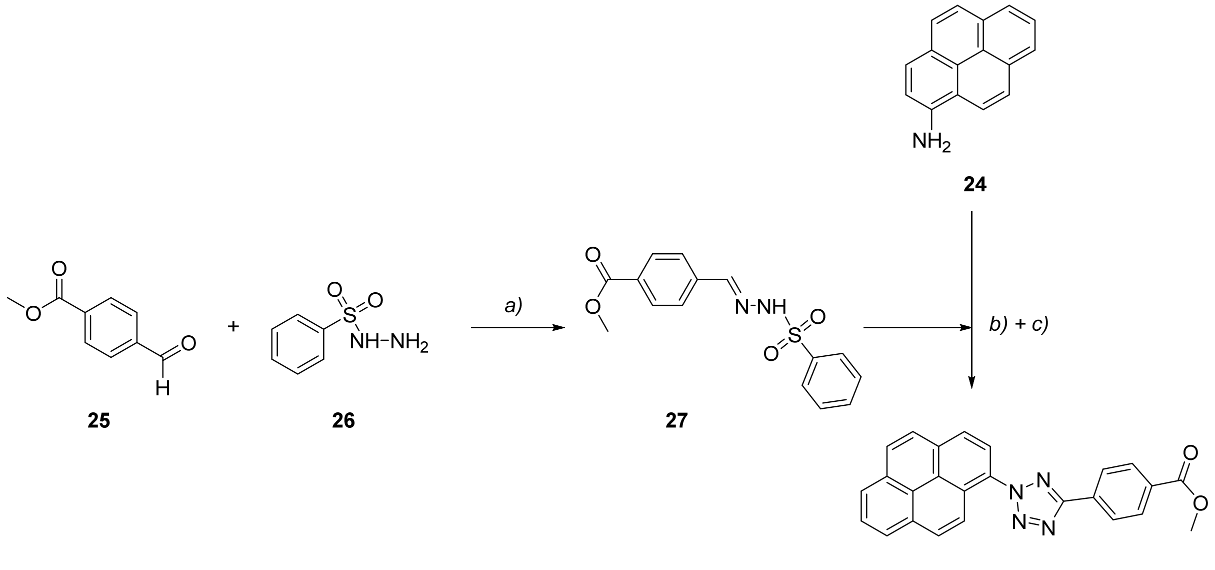

1

Scheme S1: Synthesis of **1**. a) EtOH, 45 °C, 60 min; 95%; b) $NaBF_4$, HBF_4 (50 %), -10 °C, 30 min; c) hydrazone, pyridine, 0 °C, 10 h, 31%.

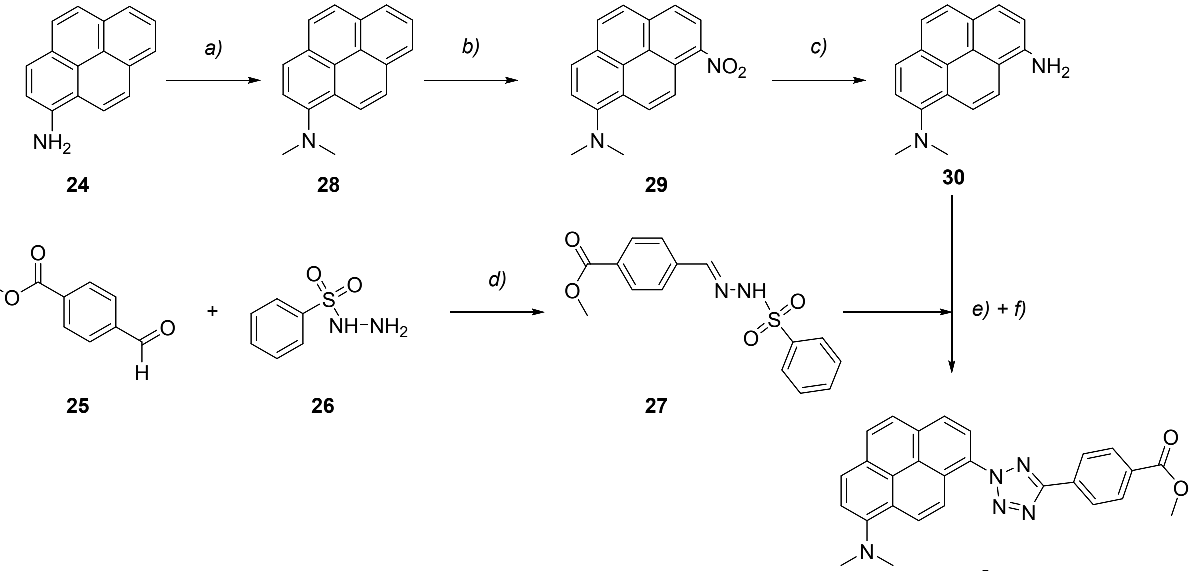

2

Scheme S2: Synthesis of **2**. a) MeI, K_2CO_3, DMF, 120 °C, 2 h, 90%; b) HNO_3, AcOH, Ac_2O, r.t., 3 h, 51%; c) H_2, Pd/C, EtOAc, r.t., 30 min, 91%; d) EtOH, 45 °C, 60 min; 95%; e) HCl, $NaNO_2$, THF, EtOH/H_2O, 0 °C, 10 min; f) hydrazone, pyridine, 0 °C, 10 h, 13%.

Compound 28

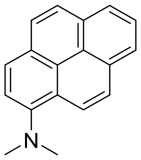

$C_{18}H_{15}N$
245,12045 g·mol^{-1}

28 was synthesized similarly to literature.[1] 2.00 g pyrene-1-amine (**24**) (9.21 mmol, 1.00 equiv.) and 6.37 g K_2CO_3 (46.1 mmol, 5.00 equiv.) were combined in a round bottom flask under argon. Dry DMF (12 mL) and 2.90 mL iodomethane (6.54 g, 46.1 mmol, 5.00 equiv.) were added drop by drop over 10 min, while stirring the reaction solution. The flask was equipped with a balloon filled with argon und heated to 125 °C for 5 min and cooled down. Excessive iodomethane was quenched with 12 mL MeOH and the reaction was stirred for another 10 min. 250 ml EtOAc were added. The mixture was extracted three times with 300 ml water. The combined organic layer was dried over $MgSO_4$, and the solvent evaporated under reduced pressure. The product has been isolated as a brown oil and was used without further purification (2.17 g, 96%). The spectral data is in agreement with literature.[1]

^{1}H NMR (400 MHz, $CDCl_3$) δ(ppm) = 8.49 (d, *J* = 9.2 Hz, 1H, H-Pyrene), 8.16 – 8.08 (m, 5H, H-Pyrene), 8.01 – 7.92 (m, 3H, H-Pyrene), 7.76 (d, *J* = 8.3 Hz, 1H, H-Pyrene), 3.08 (s, 6H, 2xCH_3).

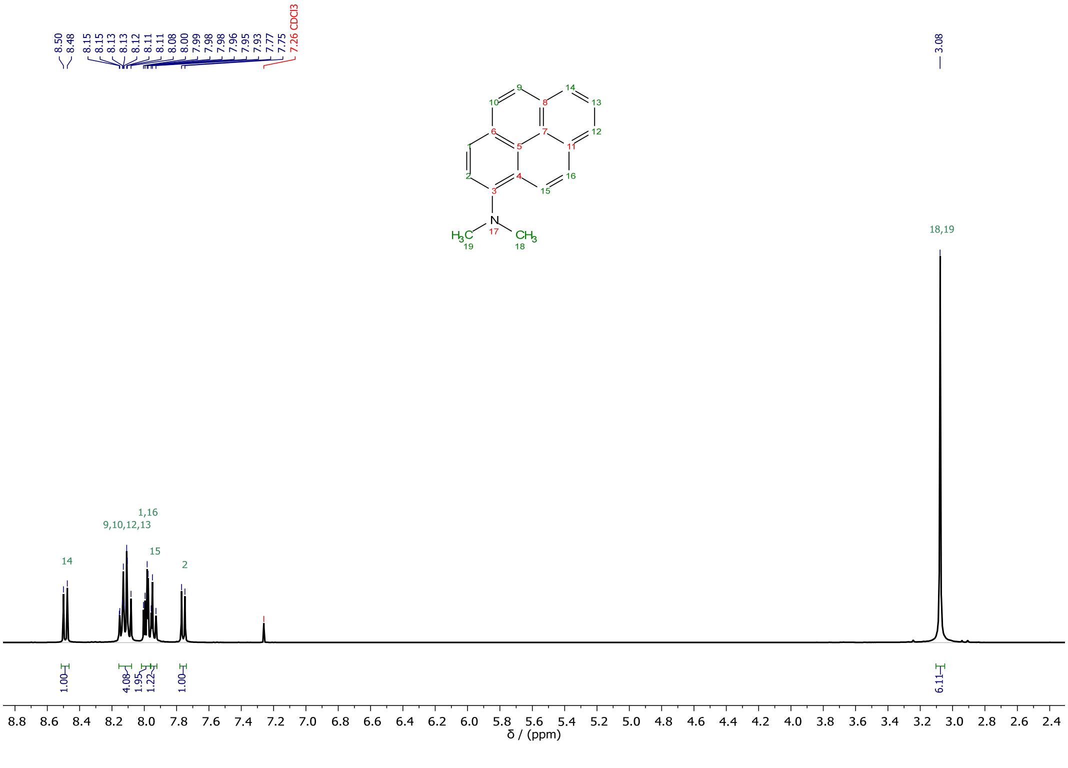

Figure S2: ^{1}H NMR spectrum of **28** ($CDCl_3$, 400 MHz).

Compound 29

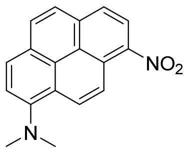

$C_{18}H_{14}N_2O_2$
290,10553 g·mol-1

29 was synthesized similarly to literature[1] 3.81 g **28** (15.5 mmol, 1.00 equiv.) was dissolved in AcOH (200 mL) and Ac_2O (60 mL). While stirring, HNO_3 (1.43 mL, 65%, 2.15 g, 34.2 mmol, 2.20 equiv.) in 10 mL AcoH was added dropwise over 1 h to the reaction solution. According to t.l.c., there was still starting material present in the solution. 0.220 eq. HNO_3 (0.143 ml, 0.250 g, 3.42 mmol, 0.220 equiv.) was added dropwise over 30 min. After 3 h, the reaction mixture got dark red, which indicated the product. The solution was quenched with 150 mL H_2O and extracted four times with 200 mL CH_2Cl_2. The organic layer was washed twice with 300 ml water, dried over $MgSO_4$ and evaporated under reduced pressure. The product was adsorbed onto silica and purified by column chromatography (silica, cyclohexane:EtOAc gradient 25:1-10:1). The product was obtained as a dark red powder (2.34 g, 52%). The spectral data is in agreement with literature.[1]

^{1}H NMR (400 MHz, $CDCl_3$) δ (ppm) = 8.93 (d, *J* = 9.7 Hz, 1H), 8.70 – 8.61 (m, 2H), 8.23 (dd, *J* = 8.4, 1.5 Hz, 1H), 8.15 – 8.10 (m, 1H), 8.04 (d, *J* = 8.5 Hz, 1H), 7.92 (d, *J* = 8.9 Hz, 1H), 7.79 – 7.73 (m, 1H), 3.14(d, *J*.= 1.3 Hz, 6H).

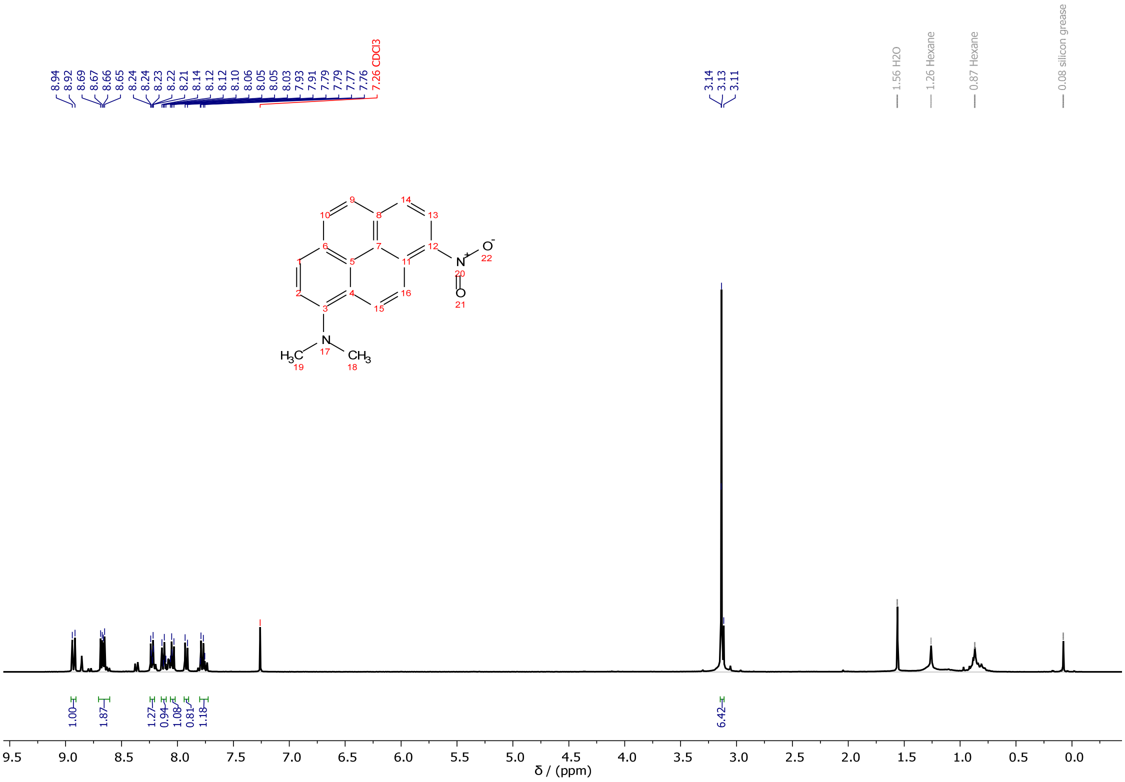

Figure S3: ^{1}H NMR spectrum of **29** ($CDCl_3$, 400 MHz).

Compound 30

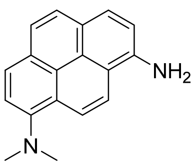

$C_{18}H_{16}N_2$
260,13135 g·mol-1

30 was synthesized similarly to literature.[1] **29** (200 mg, 0.69 mmol, 1.00 equiv.) was dissolved in EtOAc and 44 mg Pd/C (0.41 mmol, 0.6 equiv.) was added. H_2 (approx. 3 L balloon) was bubbled through the suspension for 1 h 30 min. The crude product was filtered over Celite and the solvent was removed under reduced pressure. The product was purified by column chromatography in hexane:EtOAc (99:1->4:1) and obtained as a brown oil (149 mg, 83%). The spectral data is in agreement with literature.[1]

^{1}H NMR (400 MHz, $CDCl_3$) δ (ppm) = 8.39 (d, *J* = 9.5 Hz, 1H), 8.00 (d, *J* = 8.3 Hz, 1H), 7.94 (dd, *J* = 16.9, 8.8 Hz, 2H), 7.77 (t, *J* = 11.3 Hz, 2H), 7.70 (d, *J* = 8.3 Hz, 1H), 4.43 (s, 3H), 3.04 (s, 6H).

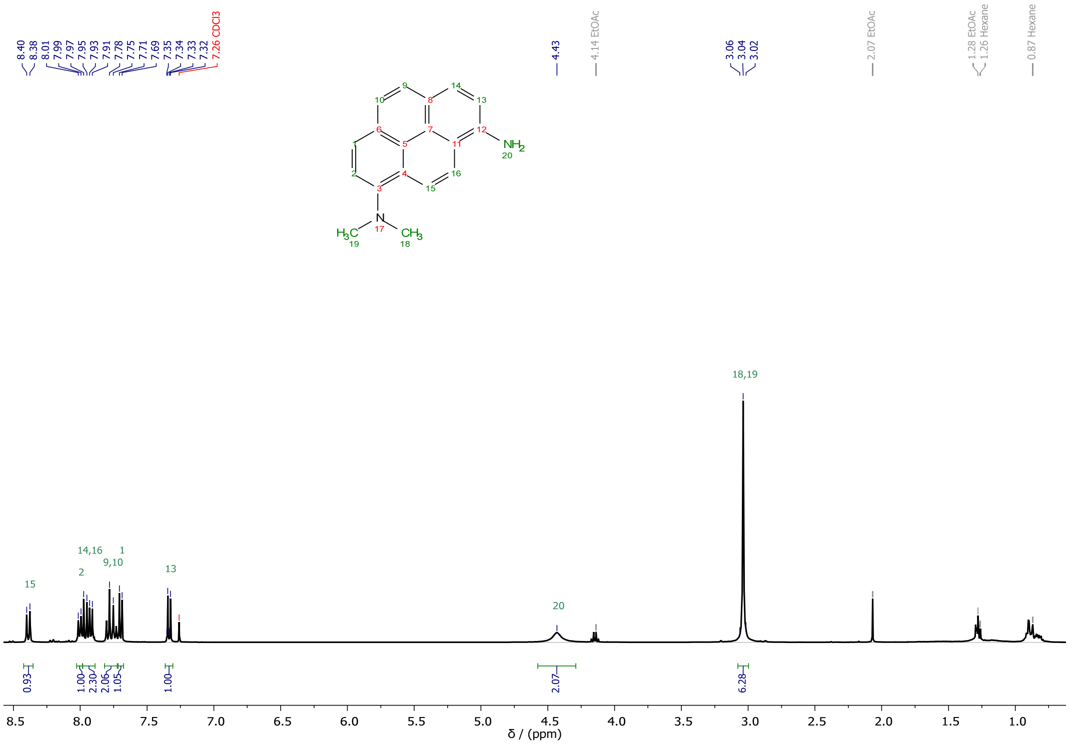

Figure S4: ^{1}H NMR spectrum of **30** ($CDCl_3$, 400 MHz).

Compound 2

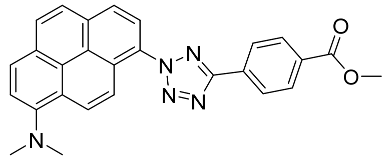

$C_{27}H_{21}N_5O_2$
447,16952 g·mol^{-1}

2 was synthesized similarly to literature.[1] 300 mg **30** (1.15 mmol, 1.00 equiv.) were dissolved in a mixture of EtOH/H_2O (3 mL each) and HCl (0.130 mL, 4.14 mmol, 3.60 equiv.) and cooled to -10 °C in an ice/salt mixture. After 20 min of stirring the aqueous solution, a solution of 95 mg $NaNO_2$ (1.38 mmol, 1.20 equiv.) in 1 mL H_2O was cooled down to 0 °C and added dropwise while stirring. The solution turned from yellow to dark blue and was continuously stirred for 10 min. Concomitantly, a solution of 622 mg **27** (1.96 mmol, 1.70 equiv.) in 5 mL pyridine was prepared and cooled to -10 °C. Subsequently, the blue solution, the diazonium salt, was transferred to the pyridine solution at -10 °C. The round bottom flask was covered with an argon balloon and allowed to warm to r.t. overnight. Afterwards the solution was poured onto 100 mL of 1 M HCl and further stirred for 1 h. 200 mL CH_2Cl_2 were added and the reaction mixture extracted three times. Afterwards the organic layer was washed with 100 mL 1 M HCl and water until the aqueous solution was set to pH 7. The organic layer was dried over $MgSO_4$ and the solvent evaporated under reduced pressure. The product was purified via column chromatography in hexane:EtOAc (10:1). The product is obtained as yellow powder (66 mg, 13%). The spectral data is in agreement with literature.[1]

^{1}H NMR (400 MHz, DMSO) δ (ppm) = 8.55 (d, *J* = 9.6 Hz, 1H), 8.48 – 8.37 (m, 5H), 8.29 (d, *J* = 9.0 Hz, 1H, 1b3-H), 8.25 – 8.20 (m, 2H), 8.16 (d, *J* = 8.9 Hz, 1H), 8.12 (d, *J* = 9.6 Hz, 1H), 7.95 – 7.90 (m, 1H), 3.93 (s, 3H), 3.04 (s, 6H).

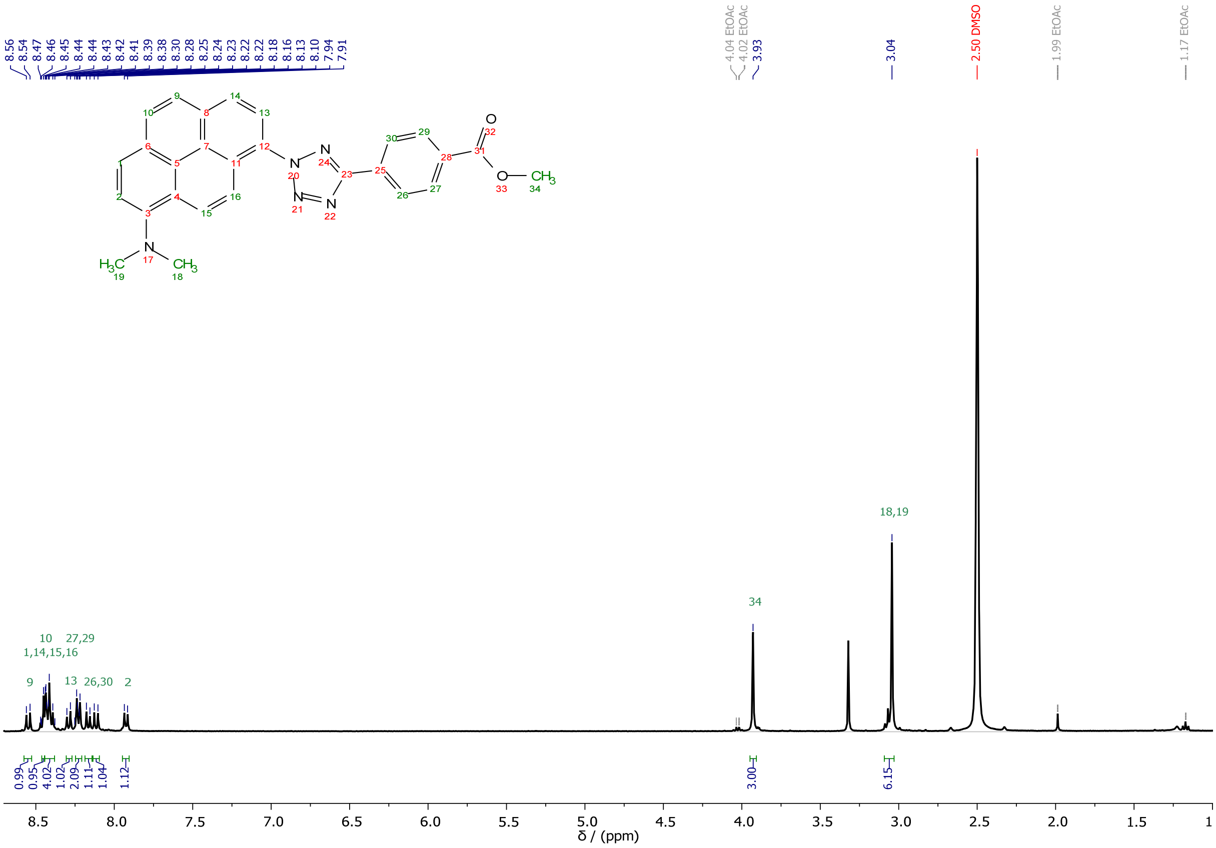

Figure S5: ^{1}H NMR spectrum of **2** (DMSO, 400 MHz).

Compound 27

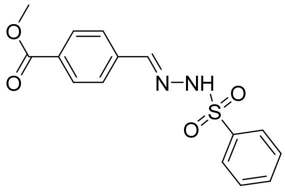

$C_{15}H_{14}N_2O_4S$
318,06743 g·mol^{-1}

27 was synthesized similarly to literature.[2] 1.90 g benzenesulfohydrazide (**25**) (11.0 mmol, 1.00 equiv.) was dissolved in 30 mL ethanol. Concomitantly, 1.70 g methyl-4-formylbenzoate (**26**) (11.0 mmol, 1.00 equiv.) was dissolved in 10 mL EtOH at 55 °C and transferred to the solution with benzenesulfohydrazide. The mixture was stirred at 40 °C for 1 h. The reaction mixture was poured on ice and diluted with 30 mL water. Subsequently the product was filtered and washed two times with cooled (0 °C) EtOH. The product was obtained as white powder (3.25 g, 95%). The spectral data is in agreement with literature.[2]

^{1}H NMR (400 MHz, DMSO) δ (ppm) = 11.81 (s, 1H, NH), 8.00-7.84 (m, 5H), 7.77-7.51 (m, 5H), 3.84 (s, 3H).

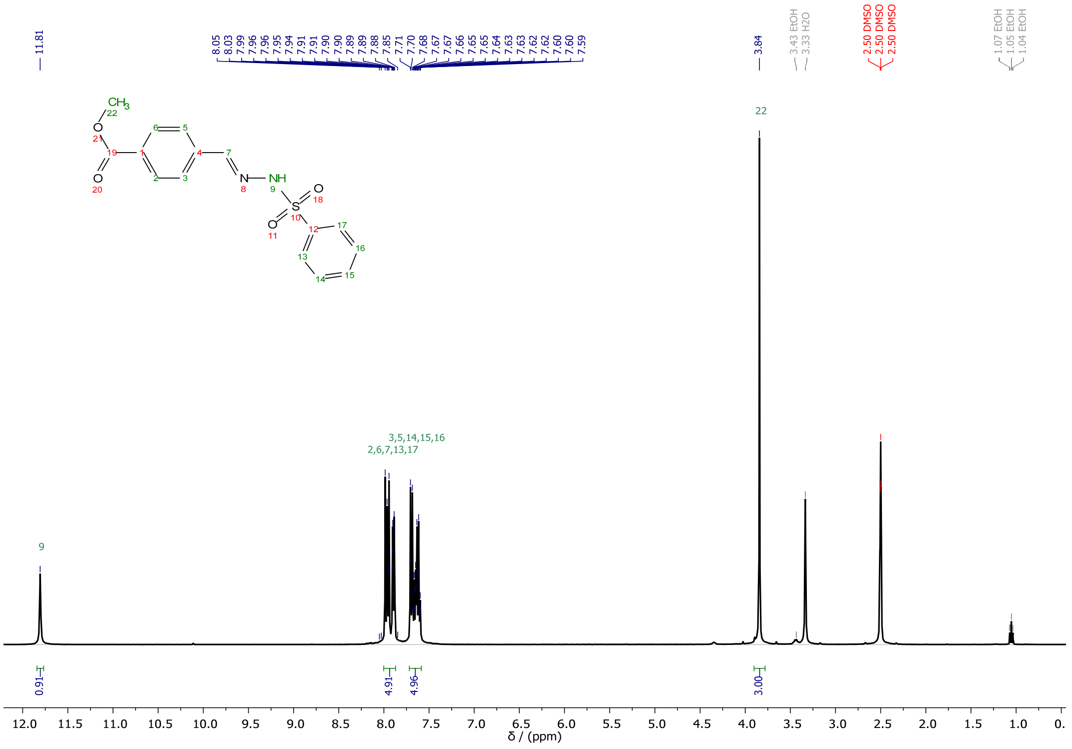

Figure S6: ^{1}H NMR spectrum of **27** (DMSO, 400 MHz).

Compound 1

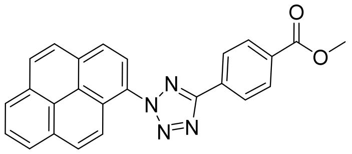

$C_{25}H_{16}N_4O_2$
404,12733 g·mol^{-1}

1.00 g amino-1-pyrene (**24**) (4.60 mmol, 1.00 equiv.) was dissolved in 120 mL THF and cooled with an ice/water mixture to -10 °C. 4.04 g $NaBF_4$ (36.8 mmol, 8.00 equiv.), 40 ml 50 % HBF_4 and 7.5 ml H_2O was added to the solution. After 20 min of stirring the aqueous solution, a solution of 381 mg $NaNO_2$ (1.42 mmol, 1.20 equiv.) in 1 mL H_2O was cooled down to -10 °C in an ice/water mixture and added dropwise while stirring for 2 h. A colour change from yellow to brown was observed. The product was filtered and added to a solution of 1.67 g **5** (5.23 mmol, 1.20 equiv.) in pyridine at -20 °C. The reaction mixture was stirred for 1 h. Afterwards the mixture was added onto a mixture of 250 mL H_2O and 50 mL concentrated HCl. The resulting solid was filtered and washed with 70 °C EtOH (558 mg, 31%).

^{1}H NMR (400 MHz, $CDCl_3$) δ (ppm) = 8.45 (d, *J* = 8.1 Hz, 2H, Pyrene-H), 8.39 (dd, *J* = 8.8, 4.6 Hz, 2H, Pyrene-H), 8.34 – 8.28 (m, 3H, Ph-H), 8.27 – 8.20 (dd, *J* = 8.6, 5.2 Hz, 4H, Pyrene-H), 8.17 (d, *J* = 8.9 Hz, 1H, Pyrene-H), 8.12 (t, *J* = 7.6 Hz, 1H, Phenyl H), 3.99 (s, 3H, Me).

^{13}C NMR (101 MHz, $CDCl_3$) δ (ppm) = 166.57, 164.56, 132.84, 131.94, 131.34, 131.15, 130.60, 130.34, 130.14, 130.09, 129.42, 127.10, 127.02, 126.92, 126.74, 126.35, 125.17, 125.05, 124.82, 124.12, 122.76, 121.43, 52.39.

ESI-HRMS m/z: calculated [M]$^+$: 404.1273; measured [M]$^+$: 404.1428.

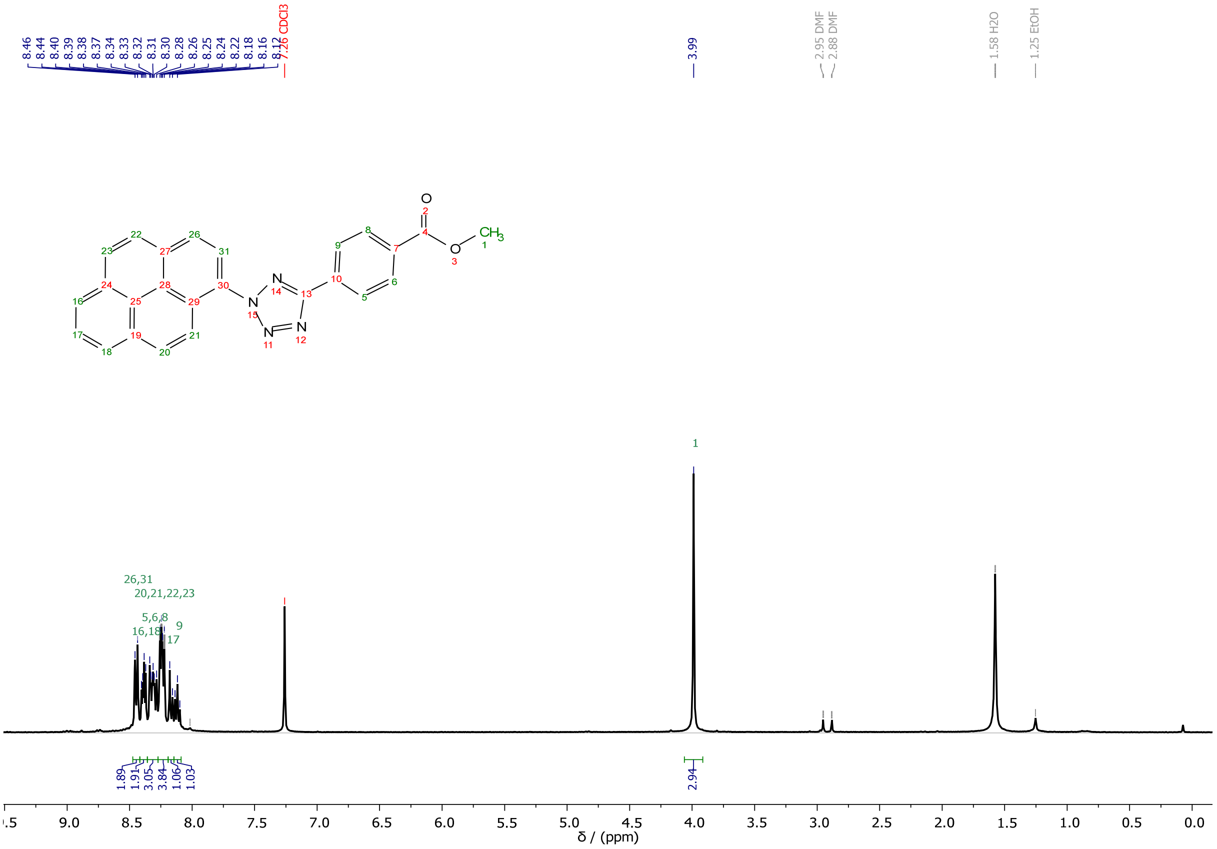

Figure S7: ^{1}H NMR spectrum of **1** ($CDCl_3$, 400 MHz).

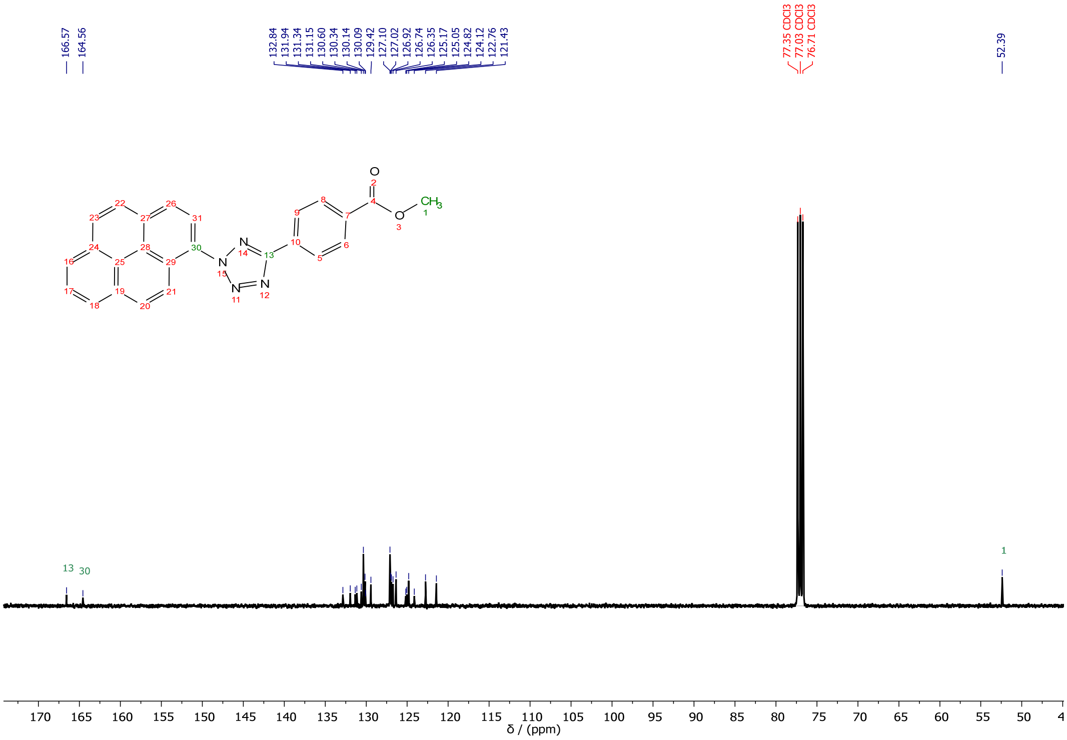

Figure S8: ^{13}C NMR spectrum of **1** (DMSO, 101 MHz).

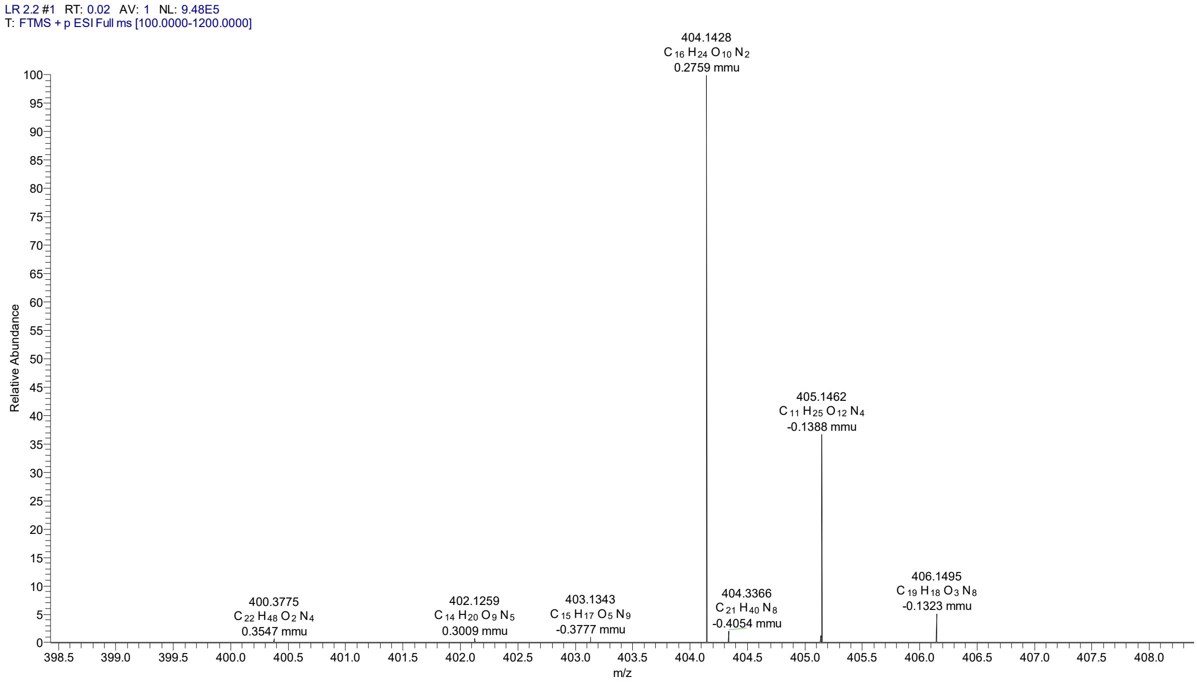

Figure S9: ESI-HRMS analysis of **1**.

b) Synthesis of 12 and 13

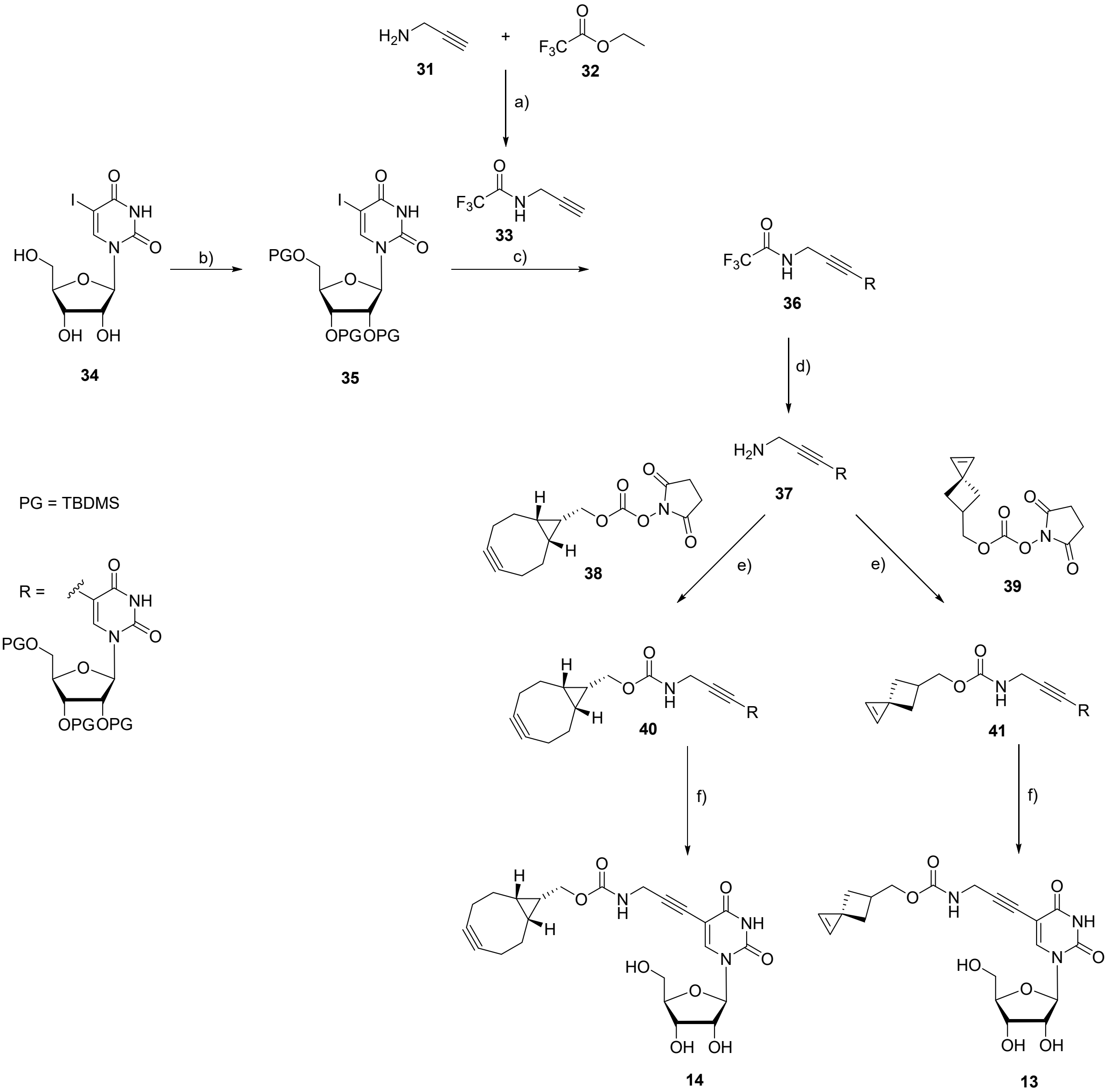

Scheme S3: Synthesis of **12** and **13**. a) MeOH, 0 °C, 30 min, r.t., 18 h, 72%; b) TBDMS-Cl, imidazole, pyridine, 50 °C, 2 h, 74%; c) $Pd(PPh_3)_4$, CuI, Et_3N, THF, 50 °C, 3 h, 74%; d) NaOH, MeOH, r.t., 2 h, quant.%; e) BCN-NHS ester (**38**) or **39**, Et_3N, THF, r.t., 19 h, **40**: 73%, **41**: 72%; f) TREAT-HF, THF, r.t., 18 h, **14**: 76%, **13**: 70%.

Compound 33

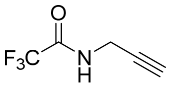

$C_5H_4F_3NO$
151,0882 g/mol

To a solution of 2.00 mL propargylamine (**31**) (1.72 g, 31.2 mmol, 1.00 equiv.) in 30 mL abs MeOH at 0 °C was slowly added 4.46 mL ethyl trifluoroacetate (**32**) (5.31 g, 37.4 mmol, 1.20 equiv.). After 30 min, the ice bath was removed, and the mixture was stirred at r.t. for 18 h. The solvent was removed under reduced pressure and the residue was dissolved in 50 mL of CH_2Cl_2. The solution was washed twice with aq. sat. $NaHCO_3$ solution (50 mL). The aqueous phase was extracted twice with 30 mL of CH_2Cl_2. The combined organic phases were washed with 50 mL of water and dried over $MgSO_4$. The solvent was removed under reduced pressure. The crude product was purified by column chromatography on silica gel (cyclohexane:EtOAc 5:1). The product **15** was obtained as a colorless oily liquid (2.67 g, 72%).

^{1}H-NMR (400 MHz, $CDCl_3$): δ (ppm) = 6.53 (br. s, 1H), 4.16 (dd, J = 5.4, 2.6 Hz, 2H), 2.35-2.34 (m, 1H).

ESI-HRMS m/z: calculated $[M]^+$: 151.02450; measured $[M+H]^+$: 152.03160.

The spectroscopic data agree with the literature.[3]

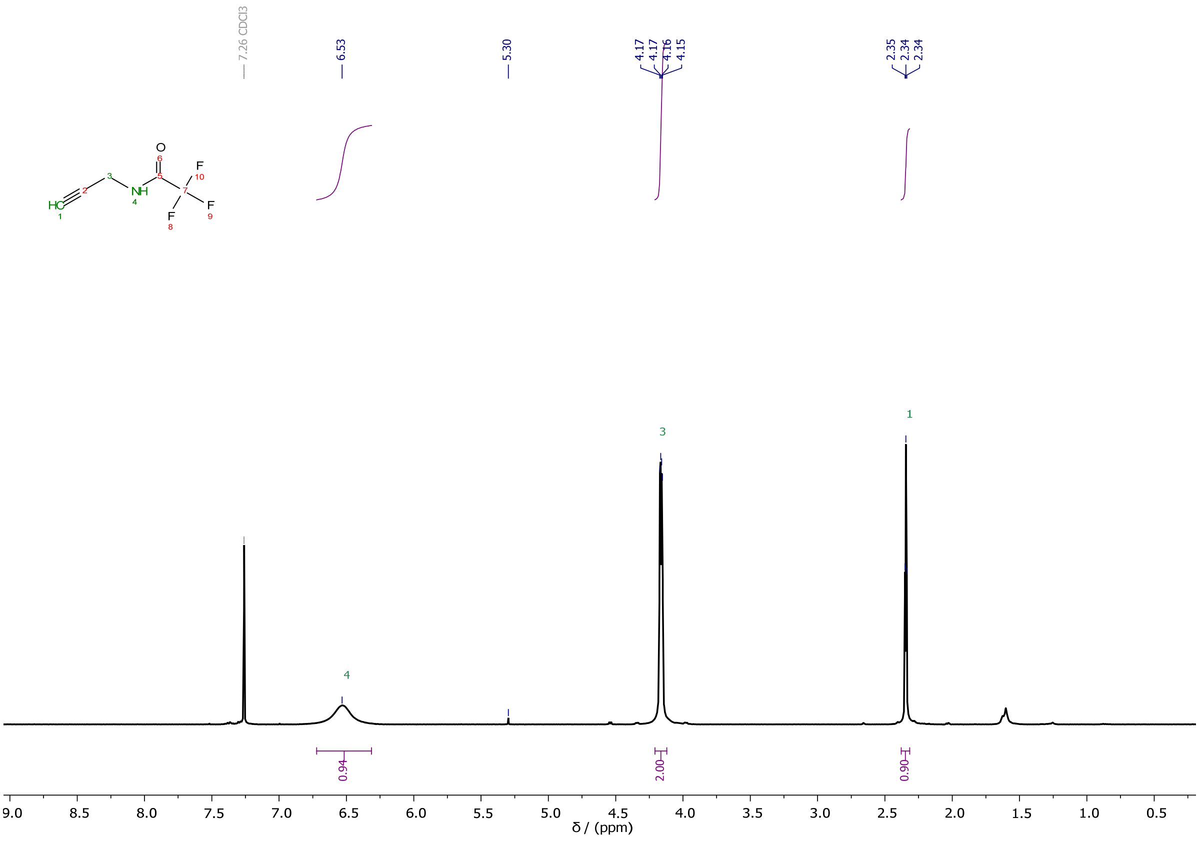

Figure S10: ^{1}H-NMR (400 MHz, $CDCl_3$) of **33.**

Compound 35

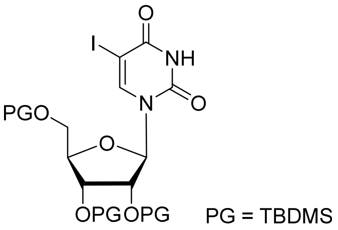

$C_{27}H_{53}IN_2O_6Si_3$
712,8885 g/mol

5-iodouridine (**34**) (1.00 g, 2.70 mmol, 1.00 equiv.), 1.63 g TBDMS-Cl (10.81 mmol, 4.00 equiv.), and 1.10 g of imidazole (16.21 mmol, 6.00 equiv.) were dissolved in 2.5 mL pyridine and then stirred at 50 °C for 2 h under argon. The mixture was cooled to r.t. and quenched by addition of MeOH (2.0 mL) with continuous stirring. After 5 min, the product mixture was dissolved in 20 mL CH_2Cl_2, added to 5% aq. HCl solution (10 mL), and extracted three times with CH_2Cl_2 (30 mL). The combined organic phases were washed with aq. 5% HCl solution (30 mL). The aqueous phases were extracted again with 30 mL CH_2Cl_2. The combined organic phases were washed with aq. sat. $NaHCO_3$ solution (40 mL) and brine (40 mL), and dried over $MgSO_4$. The solvent was removed under reduced pressure. The crude product was purified by column chromatography on silica gel (CH_2Cl_2:EtOAc 20:1). The product was obtained as a white solid (1.42 g, 74%).

^{1}H-NMR (400 MHz, $CDCl_3$): δ (ppm) = 8.21 (br. s, 1H), 8.05 (s, 1H), 6.02 (d, *J* = 6.6 Hz, 1H), 4.13 – 4.09 (m, 1H), 4.07 – 4.03 (m, 2H), 3.90 (dd, *J* = 11.6, 2.0 Hz, 1H), 3.74 (dd, *J* = 11.6, 2.0 Hz, 1H), 0.99 (s, 9H), 0.91 (s, 9H), 0.86 (s, 9H), 0.20 (s, 6H), 0.10 (s, 3H), 0.07 (s, 3H), 0.03 (s, 3H), -0.05 (s, 3H).

ESI-HRMS m/z: calculated $[M]^+$: 712.22561; measured $[M+H]^+$: 713.23203.

The spectroscopic data agree with the literature.[4]

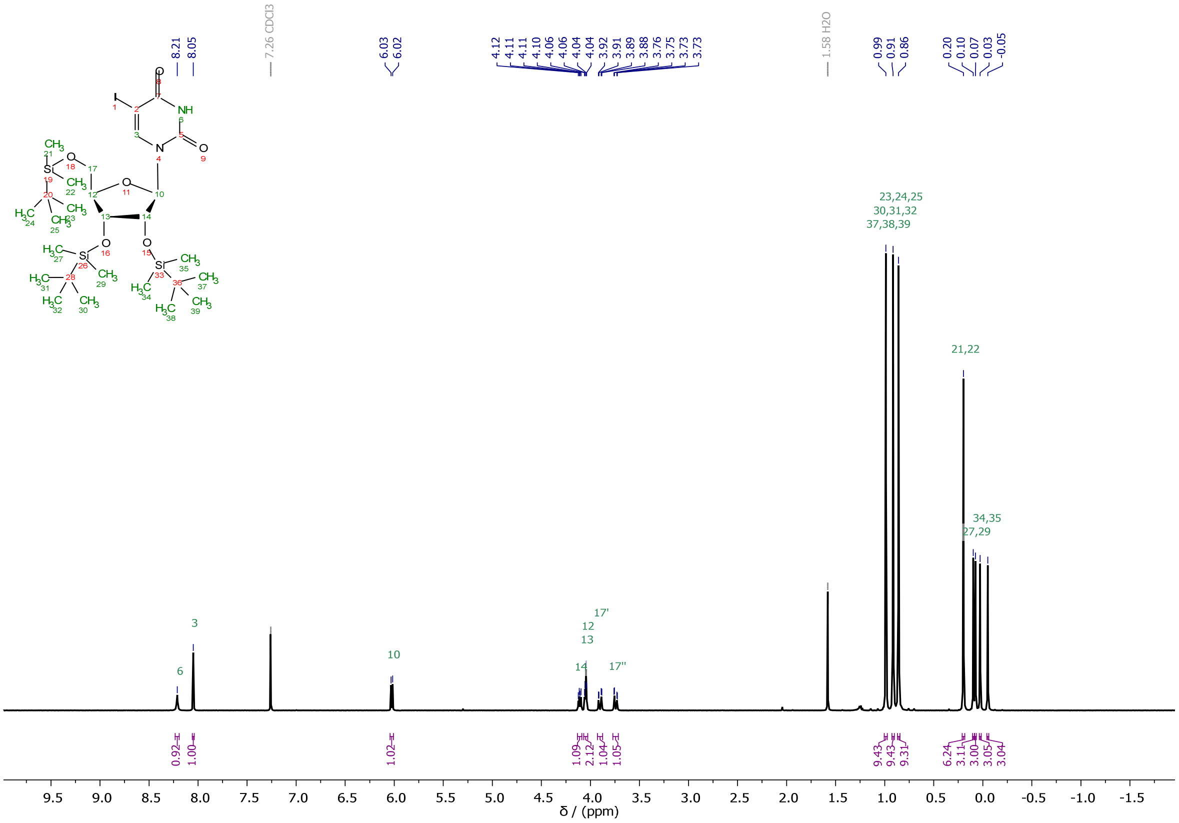

Figure S11: ^{1}H-NMR (400 MHz, $CDCl_3$) of **35**.

Compound 36

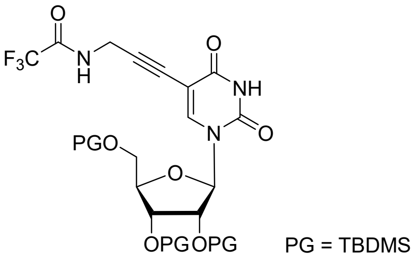

$C_{32}H_{56}F_3N_3O_7Si_3$
736,0642 g/mol

35 (0.500 g, 701 µmol, 1.00 equiv.), 27.0 mg CuI (140 µmol, 0.20 equiv.), and 81.0 mg $Pd(PPh_3)_4$ (70.1 µmol, 0.10 equiv.) were placed under an argon atmosphere and dissolved the starting in 5.00 mL of dry THF. 0.195 g **33** (1.05 mmol, 1.50 equiv.) and 389 µL Et_3N (0.284 g, 2.81 mmol, 4.00 equiv.) were added and the reaction mixture was stirred at 50 °C for 3 h. After removing the solvent under reduced pressure, the crude product was dissolved in EtOAc (50 mL), washed with aq. 5% HCl solution (40 mL), aq. sat. $NaHCO_3$ (40 mL) and brine (40 mL). The organic phase was dried over $MgSO_4$, filtered and the filtrate was concentrated under reduced pressure. The crude product was purified by column chromatography on silica gel (CH_2Cl_2:EtOAc 20:1). The product was obtained as a yellow solid (0.381 g, 74%).

^{1}H-NMR (400 MHz, $CDCl_3$): δ (ppm) = 8.13 (br. s, 1H), 8.10 (s, 1H), 5.99 (d, *J* = 5.8 Hz, 1H), 4.36 (d, *J* = 5.2 Hz, 2H), 4.13 – 4.09 (m, 1H), 4.05 (dd, *J* = 4.6, 1.7 Hz, 2H), 3.94 (dd, *J* = 11.6, 2.1 Hz, 1H), 3.75 (dd, *J* = 11.6, 1.6 Hz, 1H), 0.97 (s, 9H), 0.91 (s, 9H), 0.87 (s, 9H), 0.17 (d, *J* = 3.2 Hz, 6H), 0.09 (d, *J* = 7.3 Hz, 6H), 0.04 (s, 3H), -0.02 (s, 3H).

^{13}C-NMR (101 MHz, $CDCl_3$): δ (ppm) = 143.6, 88.4, 86.1, 76.3, 72.0, 62.7, 30.6, 26.2, 25.8, 25.7.

ESI-HRMS m/z: calculated [M]$^+$: 735.33782; measured [M+H]$^+$: 736.34470.

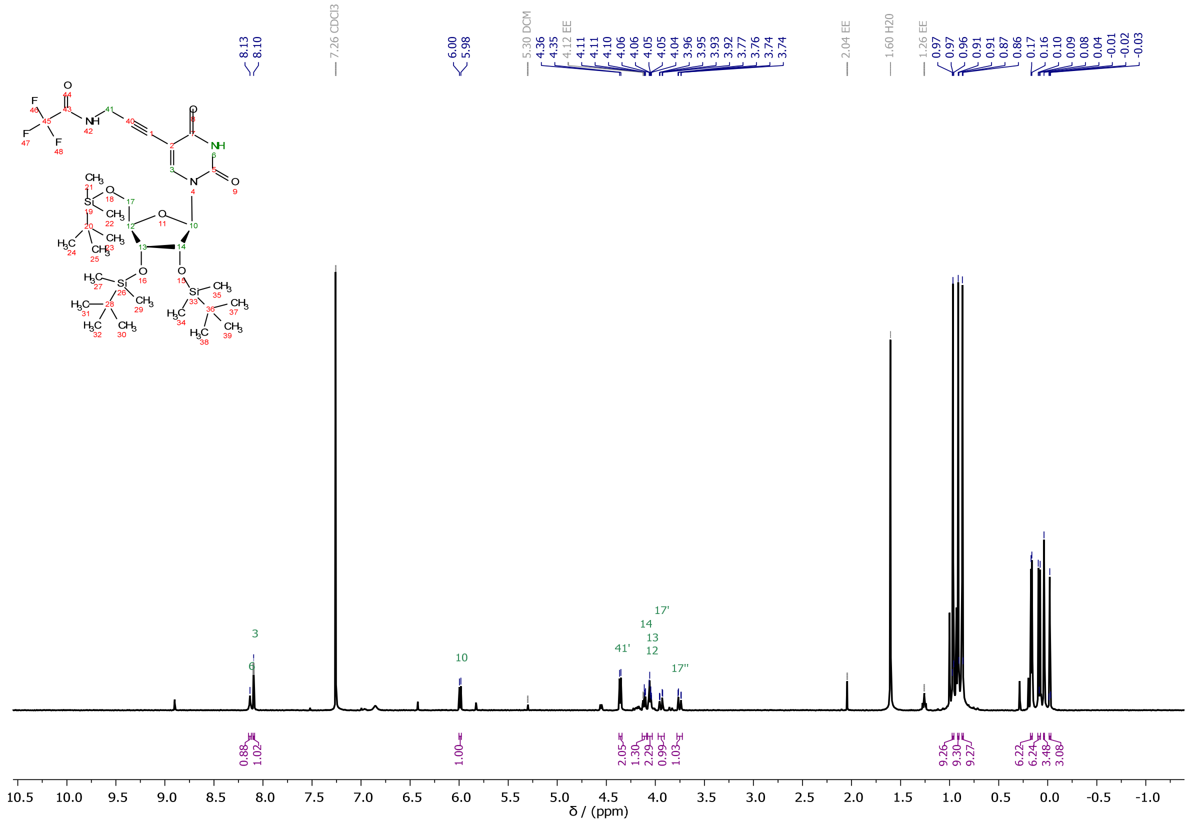

Figure S12: ^{1}H-NMR (400 MHz, $CDCl_3$) of **36**.

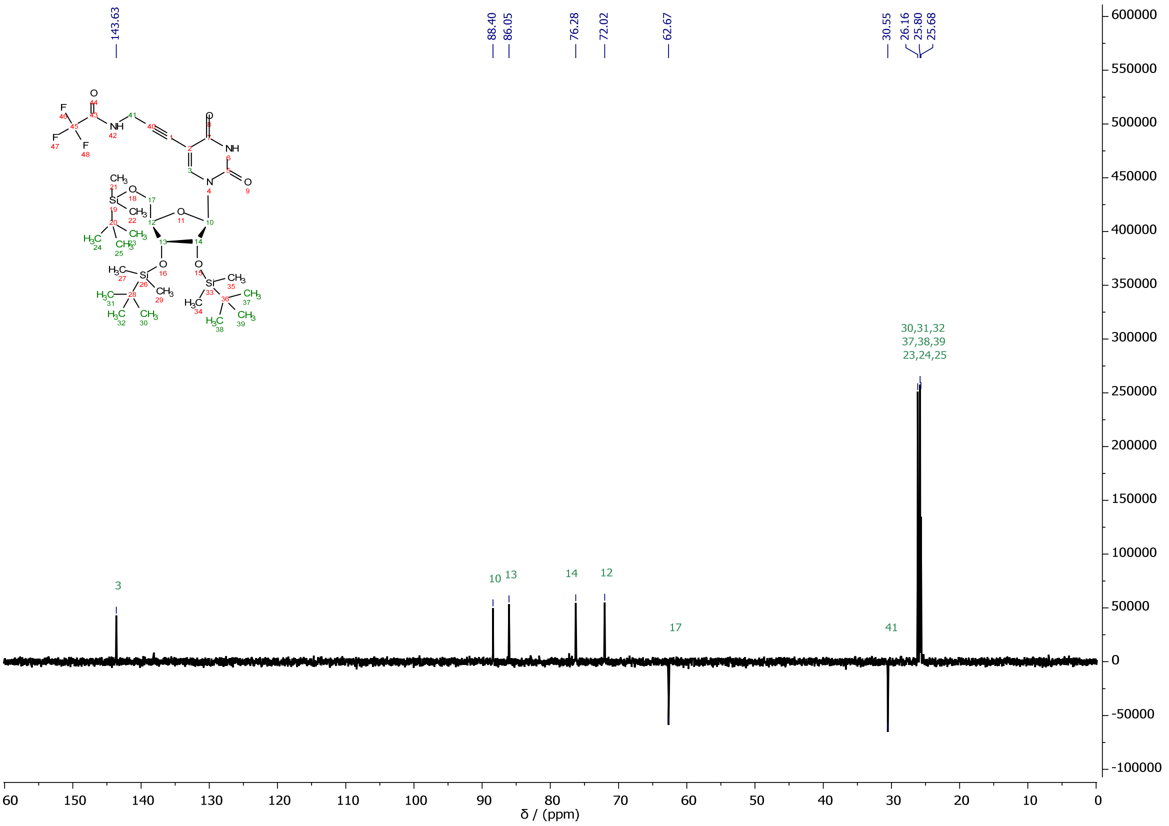

Figure S13: ^{13}C-NMR DEPT 135 (101 MHz, $CDCl_3$) of **36**.

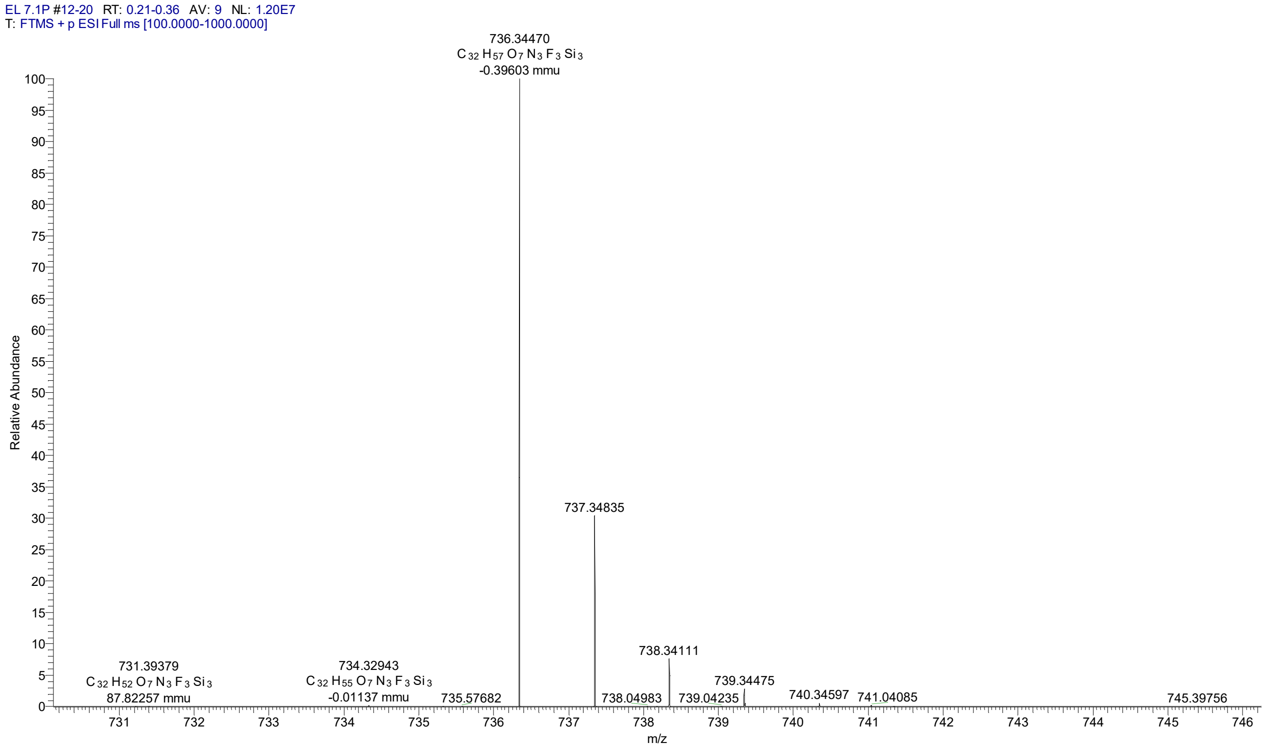

Figure S14: HR ESI-MS analysis of **36**.

Compound 37

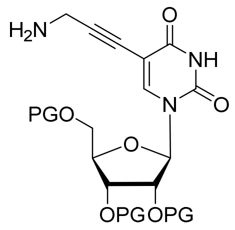

PG = TBDMS

$C_{30}H_{57}N_3O_6Si_3$
640,0560 g/mol

36 (0.400 g, 0,543 mmol, 1.00 equiv.) was dissolved in 2.80 mL of MeOH. NaOH (0.130 g, 2.36 mmol, 6.00 equiv.) dissolved in 280 µL H_2O were added dropwise. The reaction mixture was stirred at r.t. for 2 h. The solvent was removed under reduced pressure and the residue was redissolved in 40 mL CH_2Cl_2. It was washed twice with brine (30 mL), dried over $MgSO_4$, filtered and the filtrate was concentrated under reduced pressure. The crude product was purified by column chromatography on silica gel (CH_2Cl_2:EtOAc 8:2 to CH_2Cl_2:MeOH 5%). The product was obtained as a pale brown solid (348 mg, quant.).

^{1}H-NMR (400 MHz, $CDCl_3$): δ (ppm) = 7.99 (s, 1H), 5.99 (d, *J* = 5.8 Hz, 1H), 4.14 – 4.09 (m, 1H), 4.07 – 4.02 (m, 2H), 3.94 (dd, *J* = 11.7, 1.8 Hz, 1H), 3.75 (dd, *J* = 11.6, 1.6 Hz, 1H), 3.59 (s, 2H), 0.98 (s, 9H), 0.91 (s, 9H), 0.87 (s, 9H), 0.18 (d, *J* = 4.4 Hz, 6H), 0.09 (d, *J* = 7.1 Hz, 6H), 0.03 (s, 3H), -0.02 (s, 3H).

^{13}C-NMR (101 MHz, $CDCl_3$): δ (ppm) = 161.4, 149.2, 142.5, 100.3, 95.3, 88.3, 86.2, 76.2, 73.5, 72.4, 63.0, 32.5, 26.4, 26.0, 25.8, 18.7, 18.2, 18.1, -4.25, -4.45, -4.59, -4.61, -5.20, -5.22.

ESI-HRMS m/z: calculated [M]$^+$: 639.35552; measured [M+H]$^+$: 640.36273.

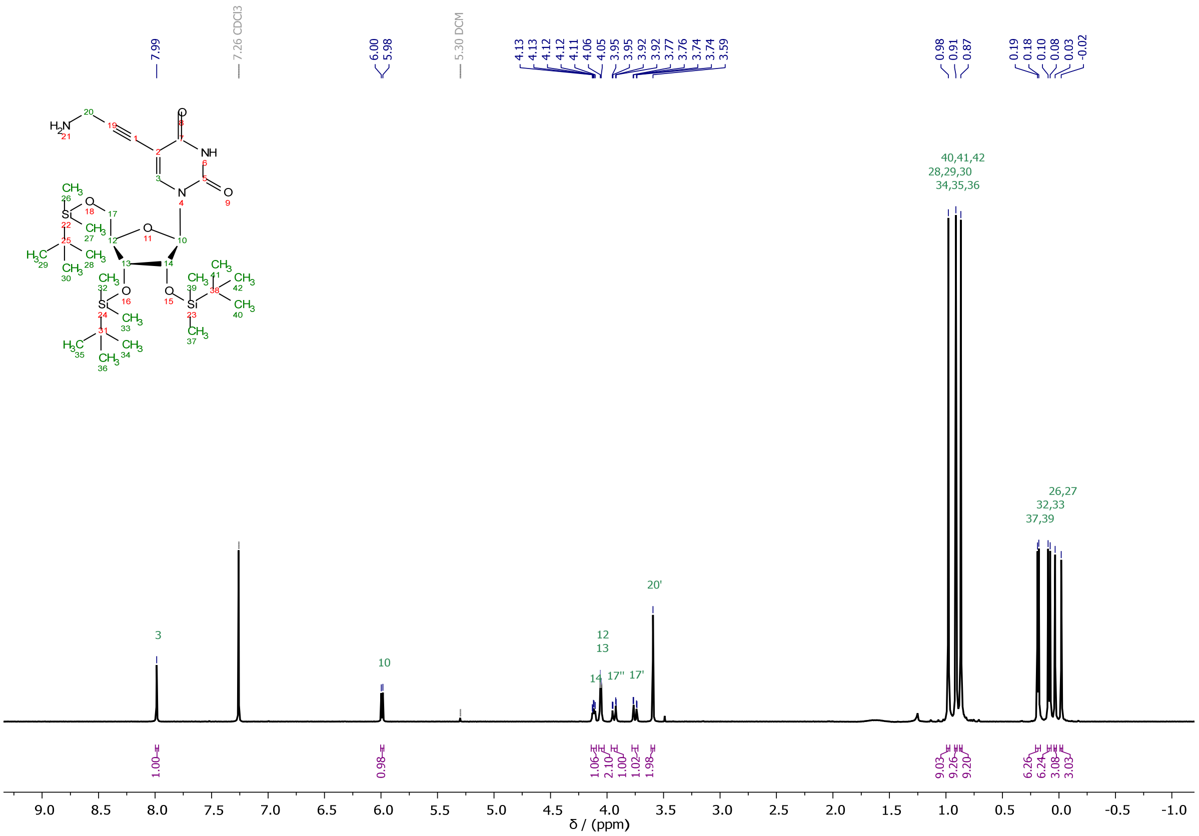

Figure S15: ^{1}H-NMR (400 MHz, $CDCl_3$) of **37**.

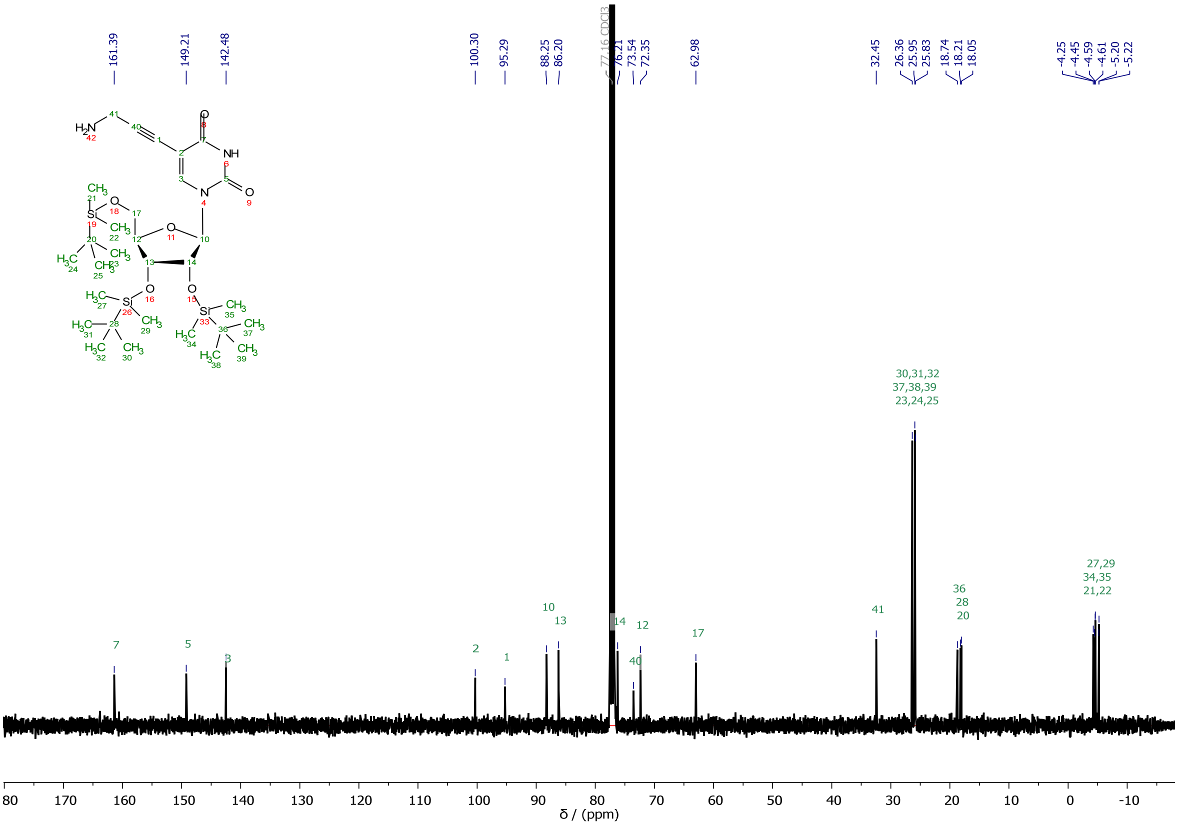

Figure S16: ^{13}C-NMR (101 MHz, $CDCl_3$) of **37**.

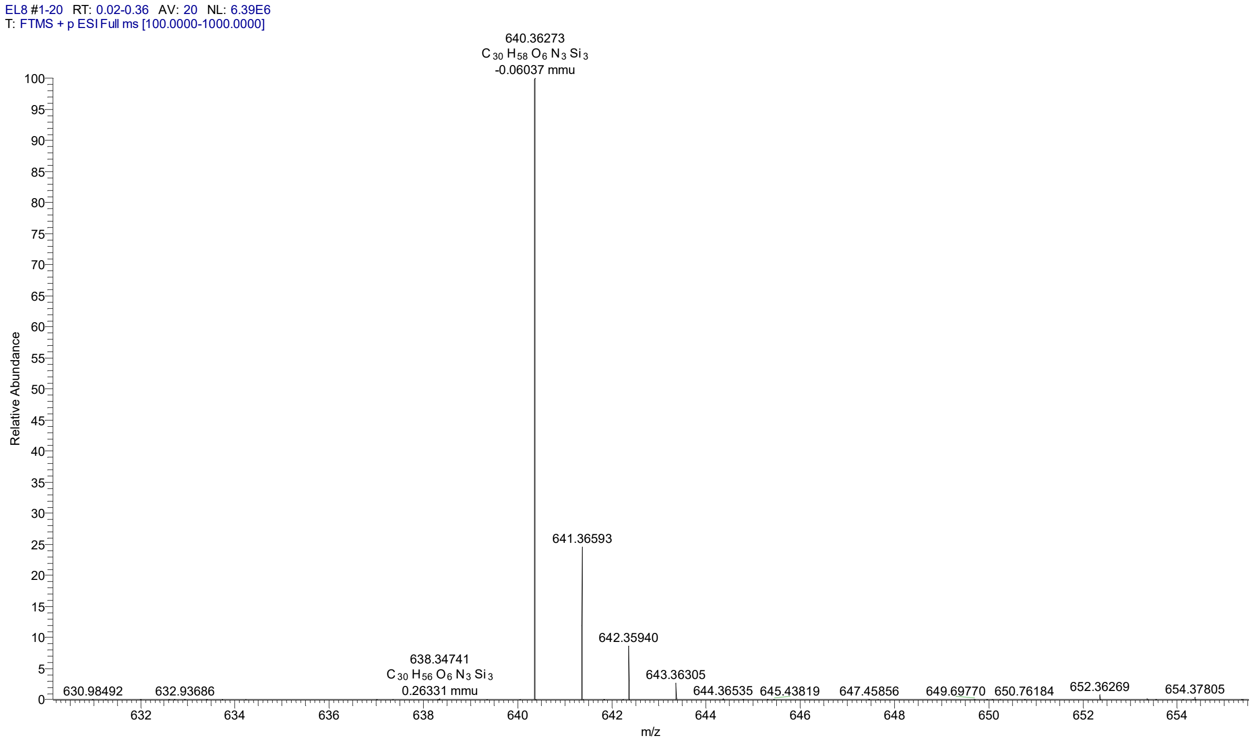

Figure S17: HR ESI-MS analysis of **37**.

Compound 39

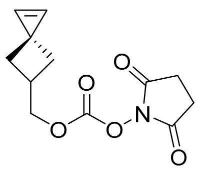

Chemical Formula: $C_{12}H_{13}NO_5$
Exact Mass: 251,07937

The precursor[5] (240 mg, 2.18 mmol, 1.00 eqiv.) was dissolved in anhydrous MeCN (5 mL). Et_3N (0.65 mL, 4.68 mmol, 2.15 equiv.) and disuccinimidyl carbonate (838 mg, 3.27 mmol, 1.50 equiv.) were added at 0°C. The reaction mixture was stirred at r.t. overnight. The solution was concentrated under reduced pressure and the crude mixture was purified by column chromatography on silica gel (hexane:EtOAc 2:1). The product was obtained as a white solid (203 mg, 37%). The spectral data is in agreement with literature.[5]

^{1}H NMR (400 MHz, $CDCl_3$) δ (ppm) = 7.41-7.40 (m, 2H, C**H**), 4.40 (d, J = 7.4 Hz, 1H, O-C**H₂**), 2.84 (s, 4H, **CH₂**), 2.66-2.58 (m, 1H, CH₂-C**H**), 2.33-2.27 (m, 2H, CH-C**H₂**-C), 1.96-1.91 (m, 2H, CH-C**H₂**-C).

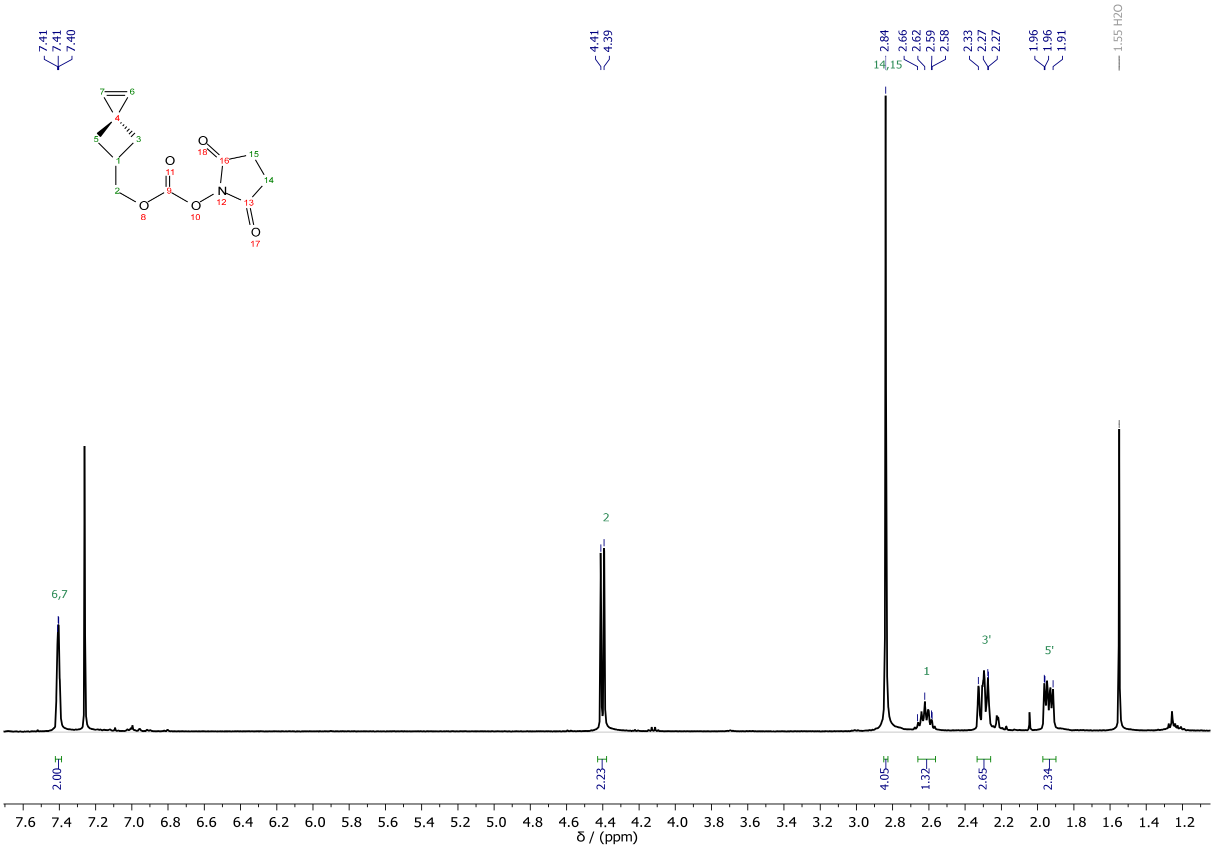

Figure S18: ^{1}H NMR (400 MHz, $CDCl_3$) spectrum of **39**.

Compound 41

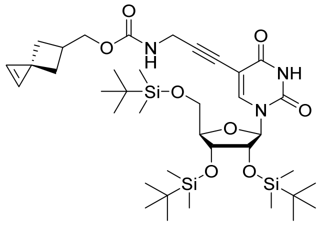

37 (170 mg, 0.266 mmol, 1.00 equiv.) and Et_3N (147 µL, 1.06 mmol, 4.00 equiv.) were dissolved in anhydrous DMF. **39** (100 mg, 0.398 mmol, 1.50 equiv.) was added and the reaction was stirred for 16 h at r.t. The solvent was removed under reduced pressure and the crude product was redissolved in MeOH (30 mL). The solution was treated with Amberlite IRA-402 bicarbonate form for 30 min. After filtration, the solvent was evaporated under reduced pressure. The crude mixture was purified by column chromatography on silica gel (CH_2Cl_2:MeOH 50:1). The product was obtained pale yellow solid (148 mg, 72%).

^{1}H NMR (400 MHz, $CDCl_3$) δ (ppm) = 8.03 (s, 1H, N**H**), 7.99 (s, 1H, **H**-6), 7.40 (dd, *J* = 8.5, 1.3 Hz, 2H, C**H**), 5.99 (d, *J* = 5.9 Hz, 1H, 1'-**H**), 4.88 (s, 1H, N**H**), 4.15-4.10 (m, 3H, O-C**H$_2$**, 2'-**H**), 4.05 (s, 2H, 3'-**H**, 4'-**H**), 3.94-3.91 (m, 1H, 5'-**H$_a$**), 3.76-3.73 (m, 1H, 5'-**H$_b$**), 2.96 (s, 1H, NH-**CH$_{2a}$**), 2.88 (s, 1H, NH-**CH$_{2b}$**), 2.47 (s, 1H, O-CH_2-C**H**), 2.25-2.19 (m, 2H, CH-C**H$_2$**), 1.89-1.84 (m, 2H, CH-C**H$_2$**), 0.97 (s, 9H, **CH$_3$**), 0.91 (s, 9H, **CH$_3$**), 0.86 (s, 9H, **CH$_3$**), 0.17 (d, *J* = 4.1 Hz, 6H, C**H$_3$**), 0.08 (d, (d, *J* = 7.4 Hz, 6H, C**H$_3$**), 0.03 (s, 3H, CH_3),-0.03 (s, 3H, C**H$_3$**).

^{13}C NMR (400 MHz, $CDCl_3$) δ (ppm) = 162.58, 161.30, 156.21, 149.07, 142.96, 121.70, 121.40, 99.58, 89.89, 88.21, 86.09, 77.35, 77.03, 76.71, 76.13, 74.57, 72.19, 69.75, 62.78, 38.26, 36.50, 31.69, 27.01, 26.20, 25.81, 25.70, 21.14, 18.60, 18.08, 17.91, -4.38, -4.60, -4.73, -5.37.

ESI-HRMS m/z: calculated [M]$^+$:775.40795; measured [M+H]$^+$:776.41435.

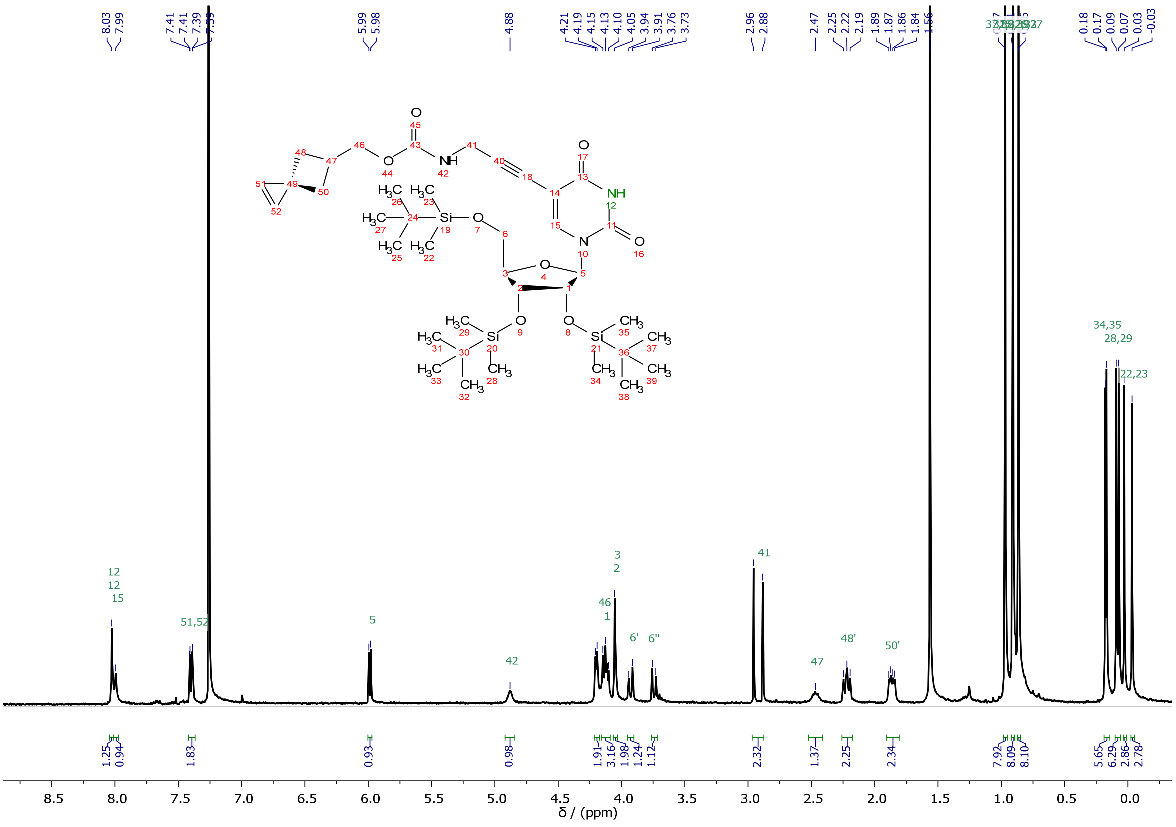

Figure S19: ^{1}H NMR (400 MHz, $CDCl_3$) spectrum of **41**.

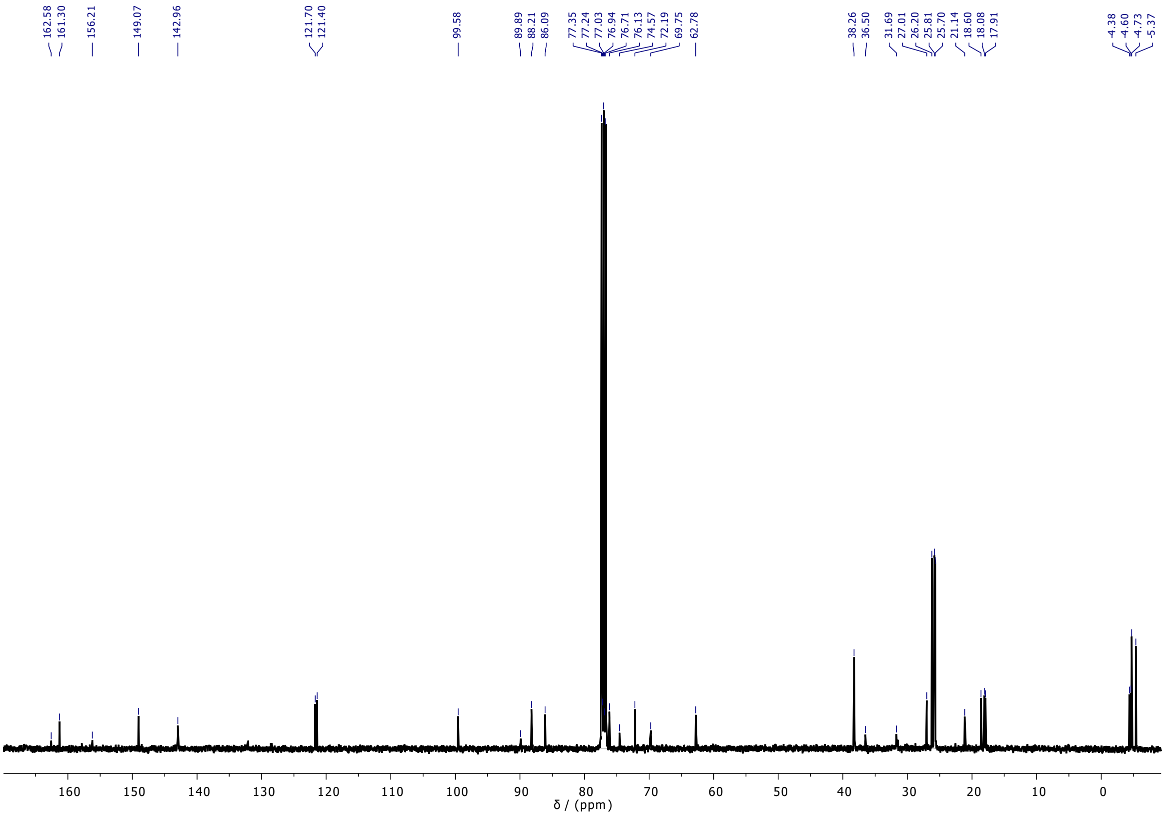

Figure S20: ^{13}C NMR (400 MHz, $CDCl_3$) spectrum of **41**.

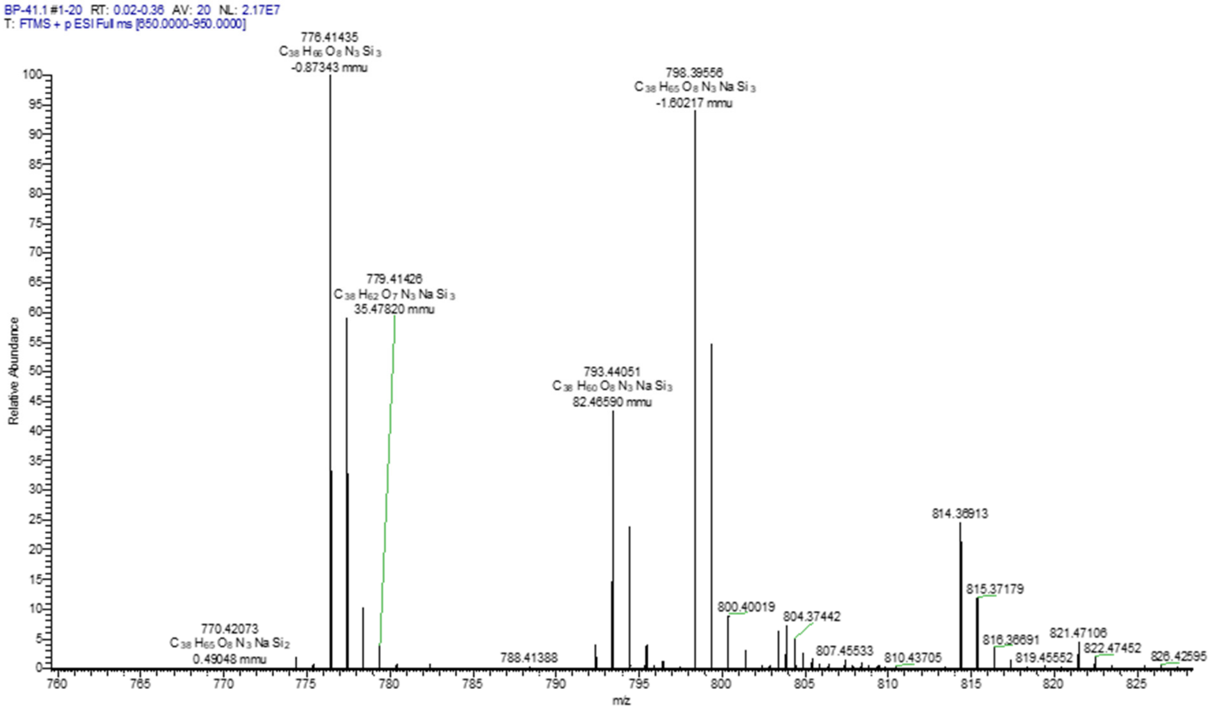

Figure S21: ESI-HRMS spectrum of **41**.

Compound 13

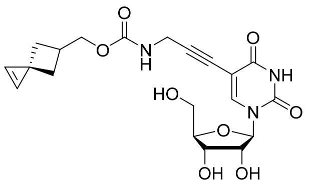

41 (148 mg, 0.190 mmol, 1.00 equiv.) and Et_3N trihydrofluoride (335 µL, 1.90 mmol, 10.0 equiv.) were dissolved in anhydrous THF. The reaction mixture was stirred for 16 h at r.t. The solvent was removed under reduced pressure and the crude product was purified by column chromatography on silica gel (CH_2Cl_2/MeOH 6:1). The product was obtained as a white solid (58 mg, 70%).

^{1}H NMR (400 MHz, DMSO) δ (ppm) = 11.63 (s, 1H, N**H**), 8.20 (s, 1H, **H**-6), 7.74 (s, 2H, C**H**), 7.61-7.63 (m, 1H, N**H**-CH_2), 5.75 (d, *J* = 5.1 Hz, 1H, 1'-**H**), 5.39 (d, *J* = 5.5 Hz, 1H, 2'-O**H**), 5.17 (t, *J* = 4.7 Hz, 1H, 5'-O**H**), 5.06 (d, *J* = 5.2 Hz, 1H, 3'-O**H**), 4.05-4.03 (m, 3H, O-C**H₂**, 2'-**H**), 3.99-3.94 (m, 3H, NH-C**H₂**, 3'-**H**), 3.85-3.84 (m, 1H, 4'-**H**), 3.67-3.63 (m, 1H, 5'-**H**$_a$), 3.58-3.54 (m, 1H, 5'-**H**$_b$), 2.43 (m, 1H, C**H**), 2.19-2.14 (m, 2H, C**H₂**), 1.83-1.79 (m, 2H, C**H₂**).

^{13}C NMR (500 MHz, DMSO) δ (ppm) = 162.03, 156.71, 150.19, 144.24, 122.30, 121.86, 98.73, 90.50, 88.59, 85.44, 74.65, 74.23, 70.08, 69.97, 60.99, 46.20, 38.52, 31.03, 27.21, 20.56.

ESI-HRMS m/z: calculated [M]$^+$: 433.1485; measured [M+Na]$^+$: 456.13704.

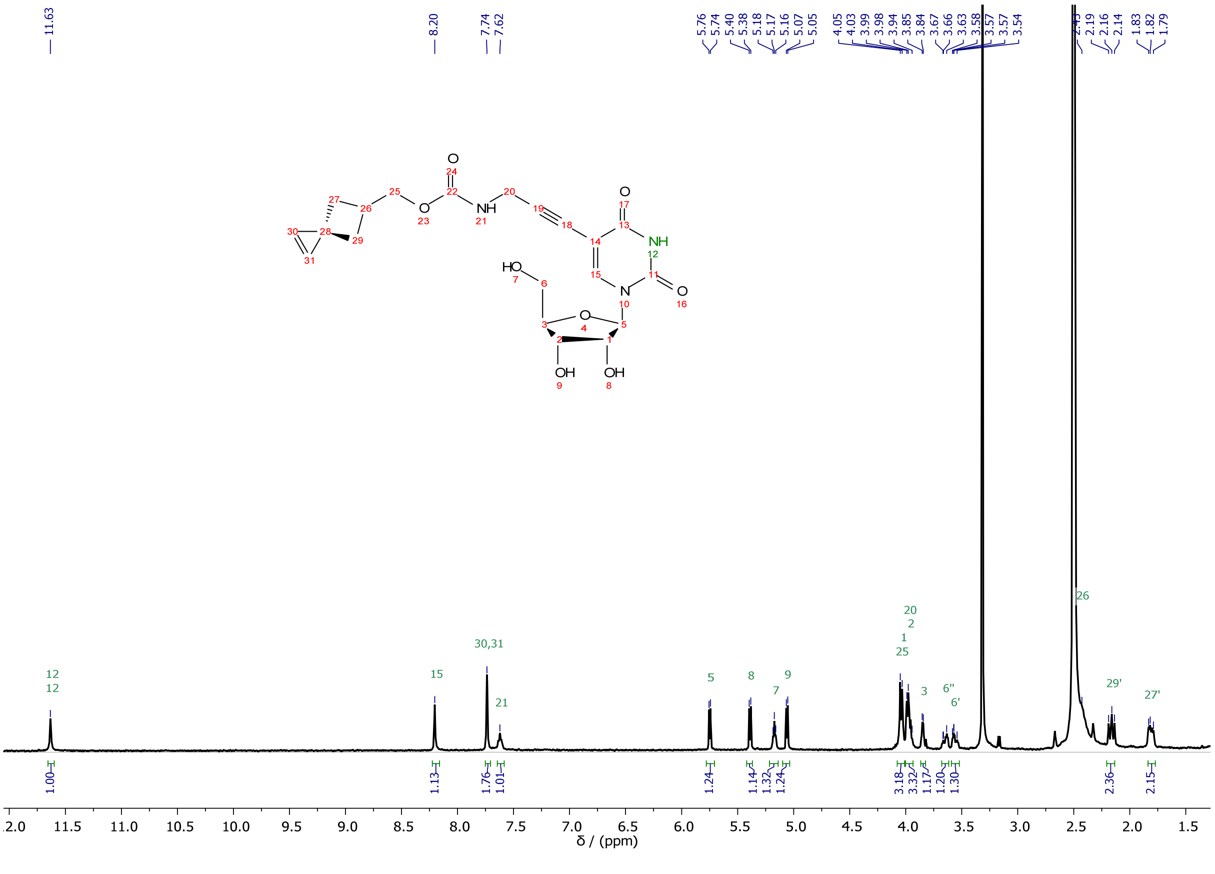

Figure S22: ^{1}H NMR (400 MHz, DMSO) spectrum of **13.**

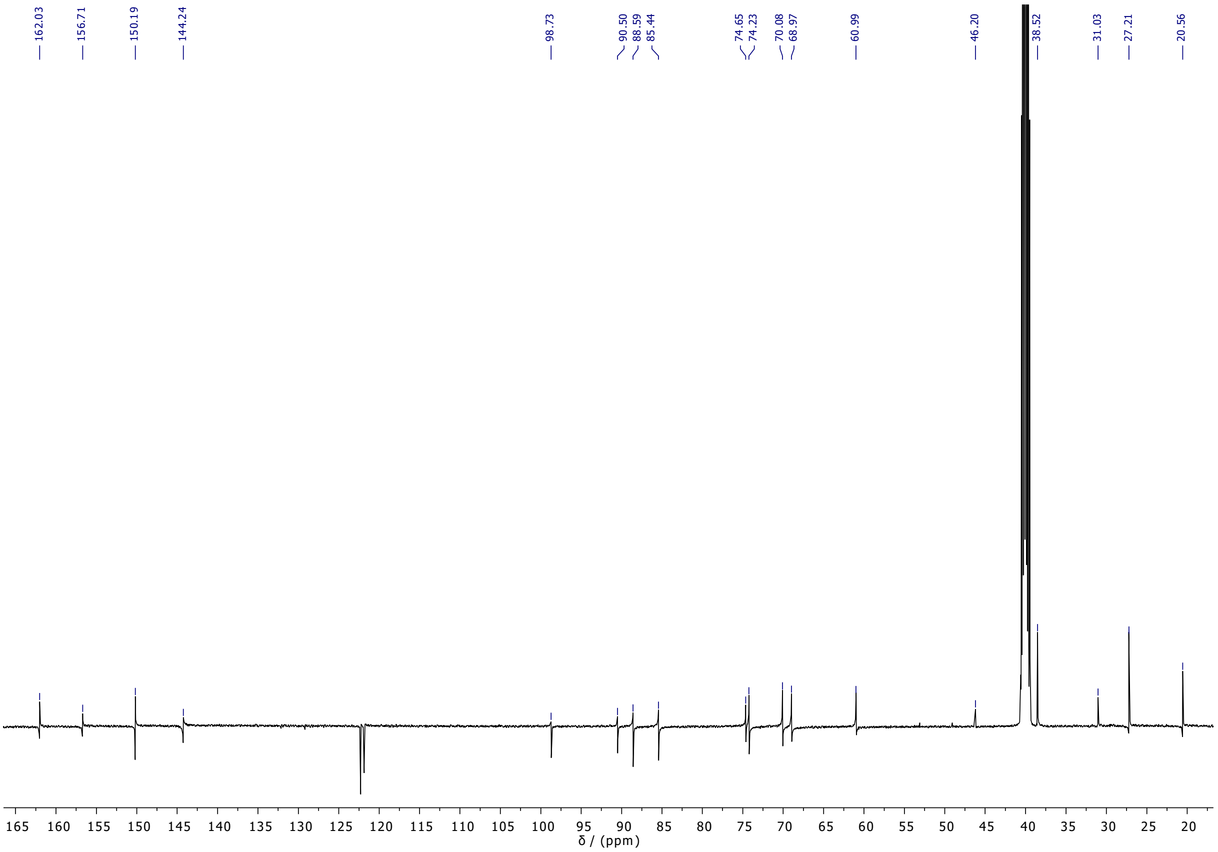

Figure S23: ^{13}C NMR (101 MHz, DMSO) spectrum of **13.**

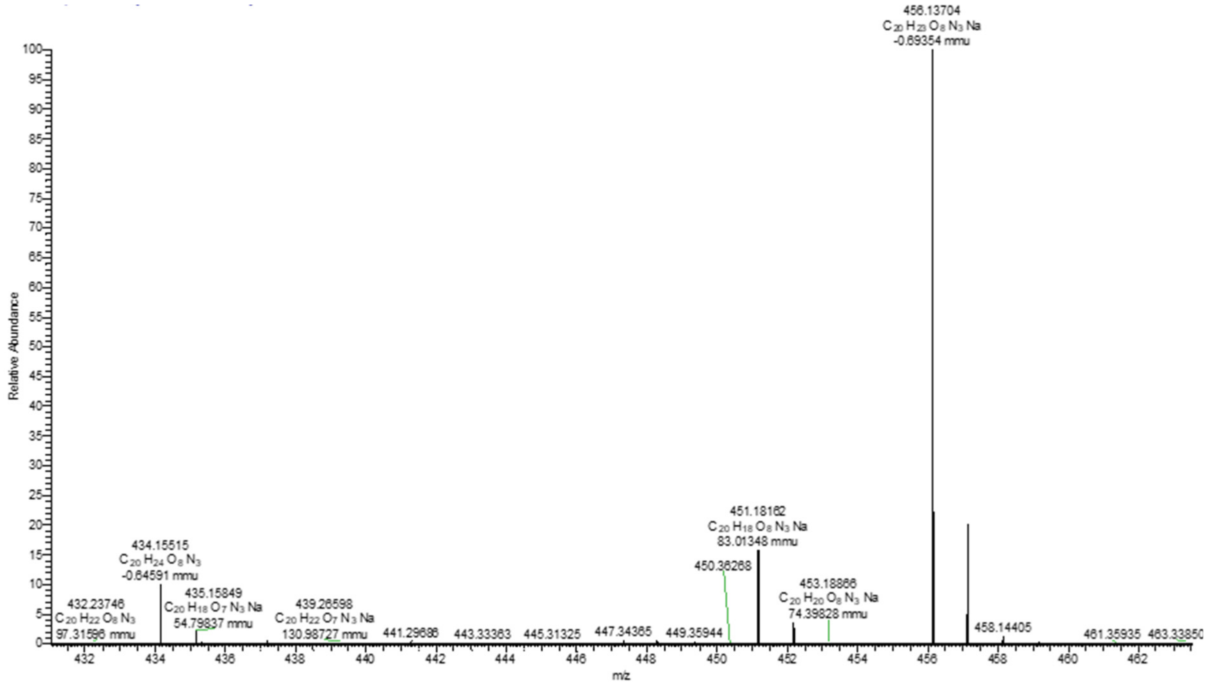

Figure S24: ESI-HRMS spectrum of **13**.

Compound 40

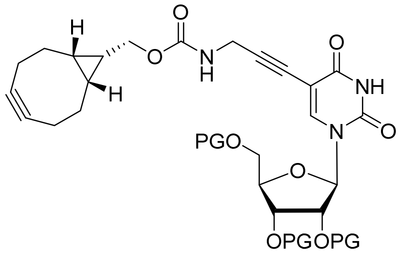

PG = TBDMS

$C_{41}H_{69}N_3O_8Si_3$
816,2710 g/mol

37 (0.104 g, 0.163 mmol, 0.95 equiv.) was dissolved in 2.00 mL dry THF. To this, 50.0 mg BCN-NHS ester (**38**) (0.172 µmol, 1.00 equiv.) and 95.0 µL Et_3N (70.0 mg, 0.687 mmol, 4.00 equiv.) were added. The reaction mixture was allowed to stir at r.t. for 19 h. The solvent was removed under reduced pressure and the residue was redissolved in CH_2Cl_2 (30 mL). It was washed with water (30 mL), aq. $NaHCO_3$ solution (2x30 mL), and brine (30 mL). The organic layer was dried over $MgSO_4$, filtered and the filtrate was removed under reduced pressure. The crude product was purified by column chromatography on silica gel (CH_2Cl_2:EtOAc 4:1). The product was obtained as a pale-yellow solid (0.195 mg, 73%).

^{1}H NMR (400 MHz, $CDCl_3$) δ (ppm) = 8.16 (s, 1H), 8.03 (s, 1H), 5.98 (d, *J* = 5.9 Hz, 1H), 4.91 (s, 1H), 4.23 – 4.14 (m, 4H), 4.14 – 4.09 (m, 1H), 4.07 – 4.03 (m, 2H), 3.93 (dd, *J* = 11.6, 1.9 Hz, 1H), 3.75 (dd, *J* = 11.6, 1.6 Hz, 1H), 2.34 – 2.16 (m, 6H), 1.66 – 1.50 (m, 4H), 1.42 – 1.31 (m, 1H), 0.97 (s, 9H), 0.91 (s, 9H), 0.87 (s, 9H), 0.18 (d, *J* = 4.1 Hz, 6H), 0.08 (d, *J* = 7.3 Hz, 6H), 0.03 (s, 3H), -0.03 (s, 3H).

^{13}C NMR (101 MHz, $CDCl_3$) δ (ppm) = 161.3, 151.2, 149.1, 143.2, 99.7, 99.0, 90.0, 88.3, 86.3, 76.3, 74.8, 72.4, 63.0, 31.9, 29.2, 26.3, 25.9, 25.8, 21.6, 20.3, 18.7, 18.2, 18.0, 17.9, -4.25, -4.45, -4.59, -4.62, -5.24.

ESI-HRMS m/z: calculated [M] $^+$: 815.43925; measured [M+H]$^+$: 816.44682.

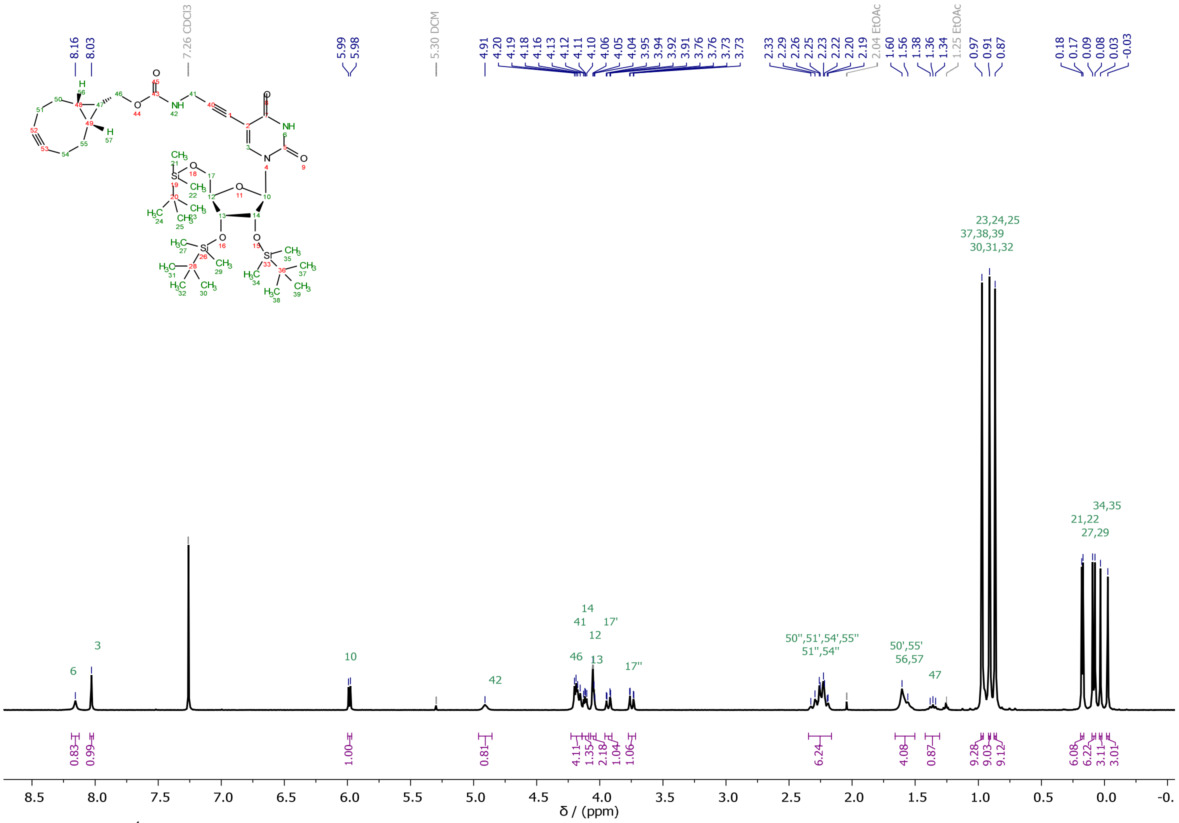

Figure S25: ^{1}H-NMR (400 MHz, $CDCl_3$) of **40**.

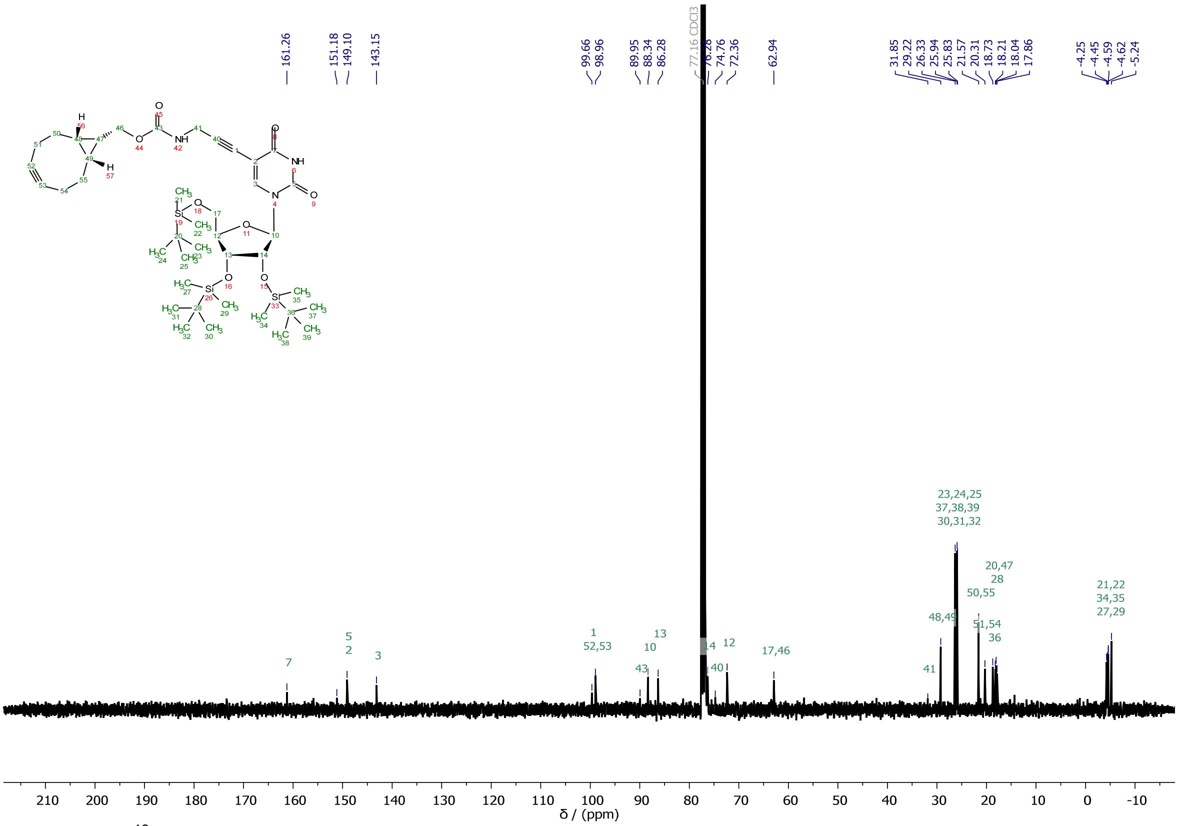

Figure S26: ^{13}C-NMR (101 MHz, $CDCl_3$) of **40**.

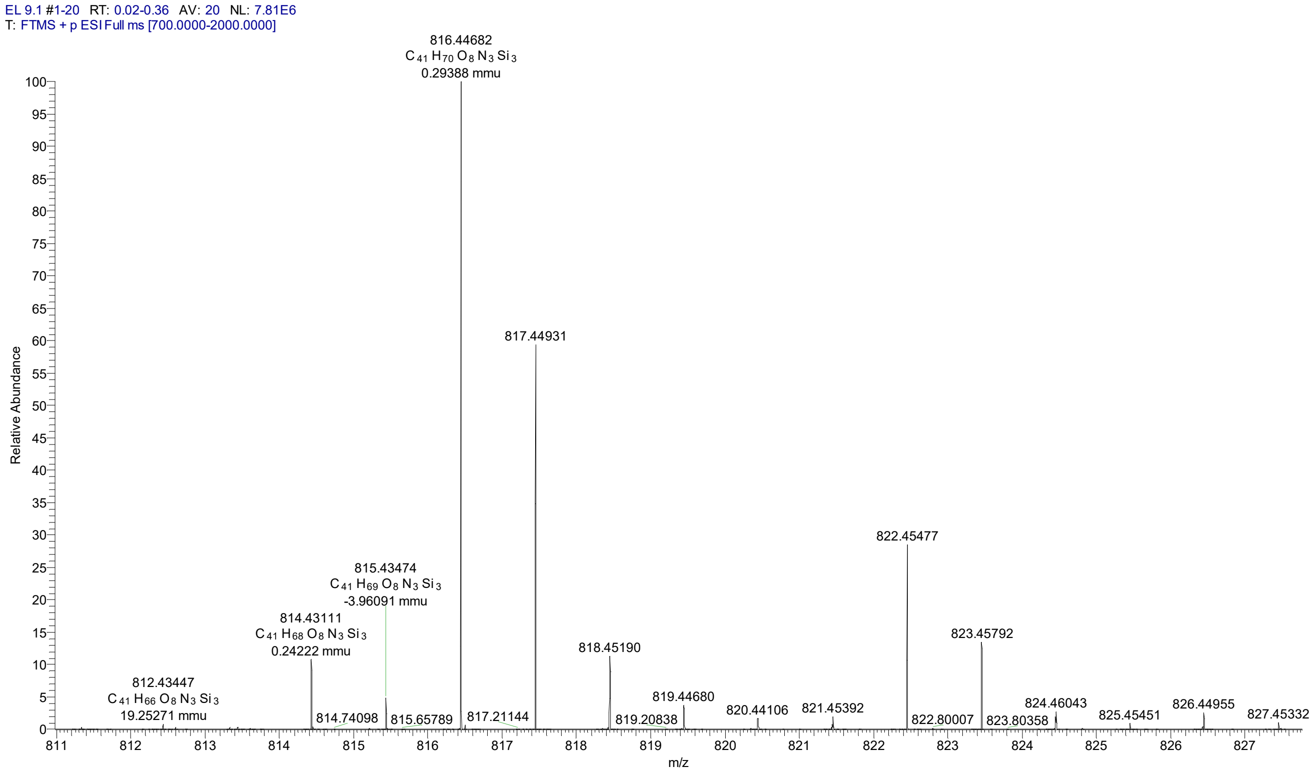

Figure S27: HR ESI-MS analysis of **40**.

Compound 14

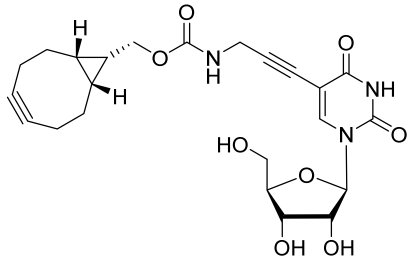

$C_{23}H_{27}N_3O_8$
473,4820 g/mol

To a solution of 0.100 g **40** (0.123 mmol, 1.00 equiv.) in 2 mL dry THF, 0.160 mL Et_3N-trihydrofluoride (TREAT-HF) (0.158 mmol, 0.980 mmol, 8.00 equiv.) were added. The reaction mixture was stirred at 50 °C for 16 h. The solvent was removed under reduced pressure and the crude product was purified by column chromatography on silica gel (CH_2Cl_2/MeOH 9:1). The product was obtained as an off-white solid (0.044 mg, 76%).

^{1}H-NMR (400 MHz, MeOD): δ (ppm) = 8.30 (s, 1H), 5.84 (d, *J* = 4.0 Hz, 1H), 4.19 – 4.09 (m, 4H), 4.06 – 4.01 (m, 2H), 4.01 – 3.96 (m, 1H), 3.84 (dd, *J* = 12.3, 2.6 Hz, 1H), 3.71 (dd, *J* = 12.3, 2.9 Hz, 1H), 2.26 – 2.08 (m, 6H), 1.63 – 1.50 (m, 2H), 1.40 – 1.28 (m, 1H), 0.96 – 0.85 (m, 2H).

^{13}C-NMR (101 MHz, MeOD): δ (ppm): 164.5, 158.8, 151.4, 145.5, 100.1, 99.5, 91.0, 90.7, 86.4, 76.0, 74.9, 71.0, 64.1, 61.9, 31.9, 30.2, 21.9, 21.4, 18.9.

ESI-HRMS m/z: calculated $[M]^+$: 473.4820; measured $[M+H]^+$: 474.18738.

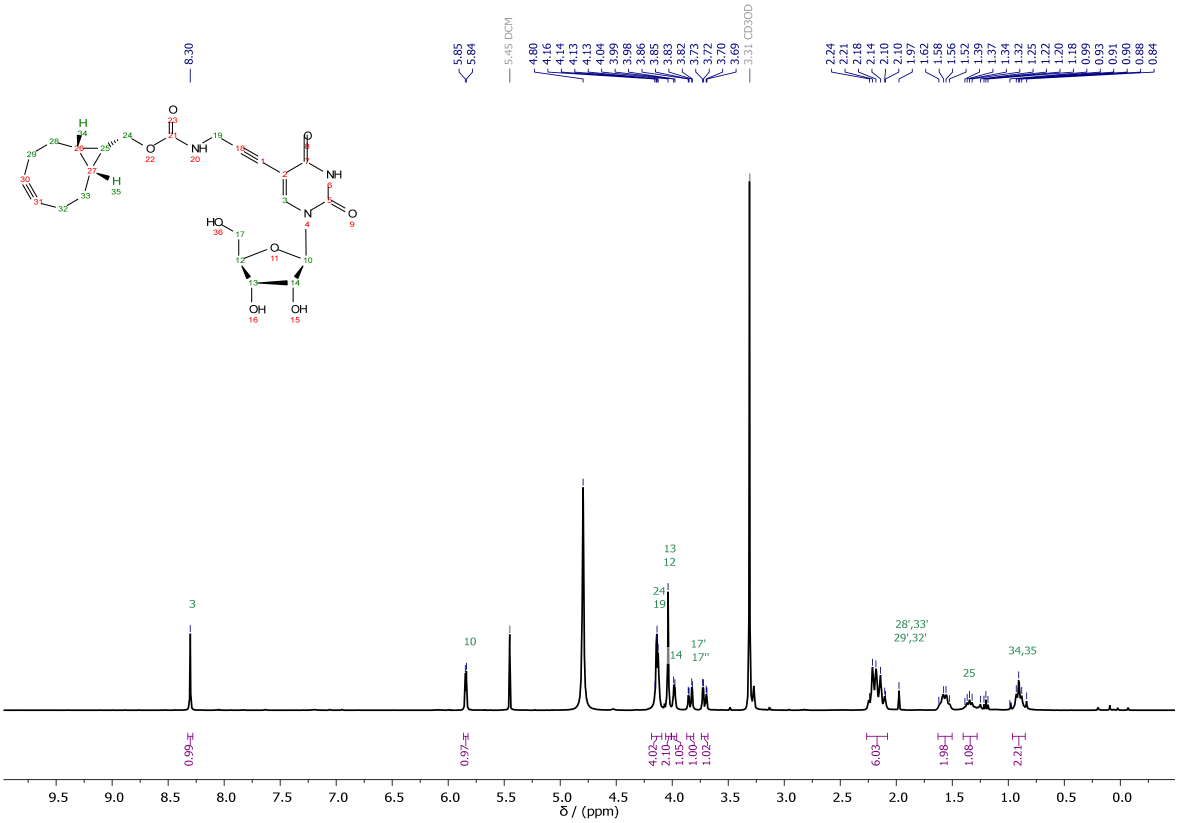

Figure S28: ^{1}H-NMR (400 MHz, MeOD) of **14**.

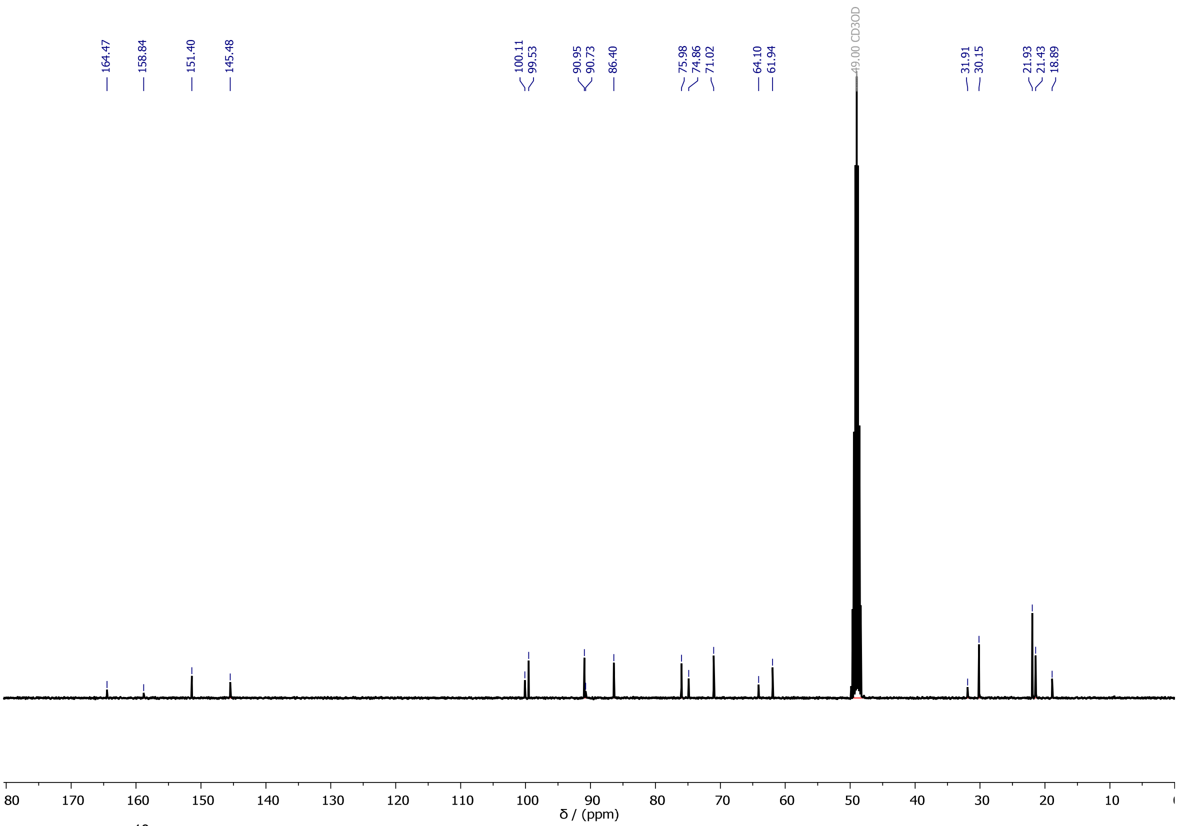

Figure S29: ^{13}C-NMR (101 MHz, MeOD) of **14.**

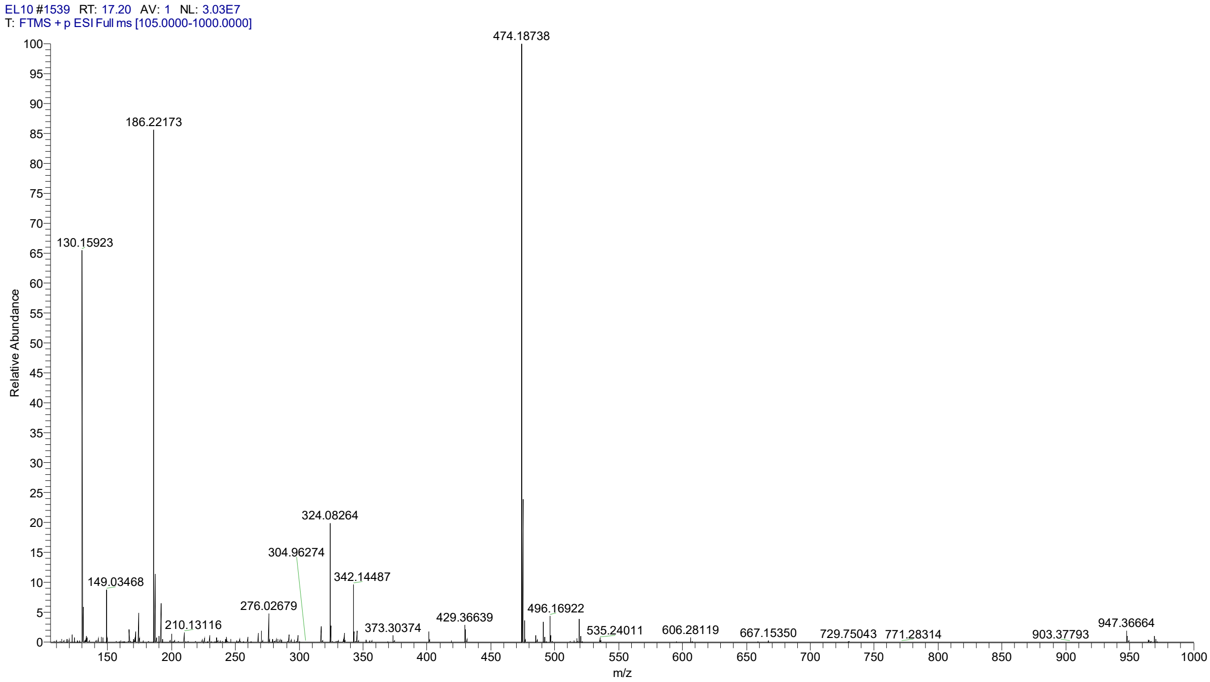

Figure S30: HR ESI-MS analysis of **14**.

c) „Photoclick“ reactions

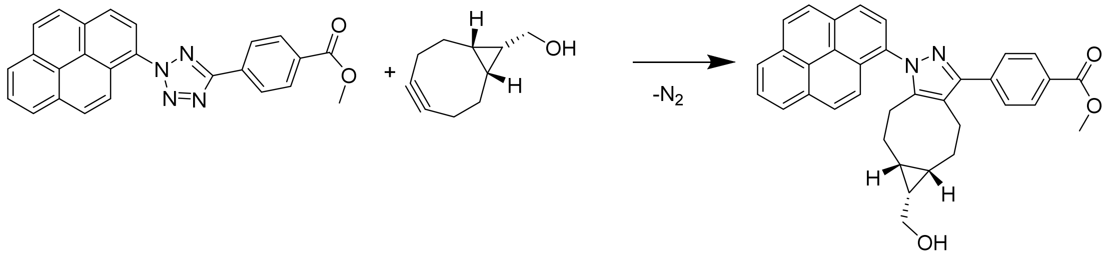

$C_{35}H_{30}N_2O_3$
526,22564

1 **11** **17**

Scheme S4: “Photoclick” reaction of **1** and **9** to **14**.

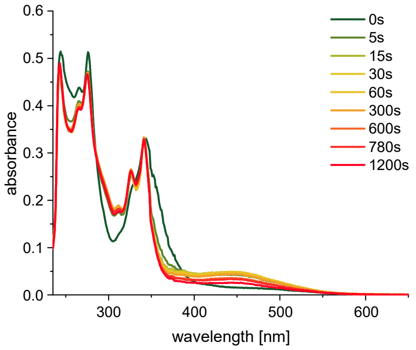

Figure S31: Time-dependent UV/Vis absorbance of the “photoclick” reaction of **1** (25 µM) and **11** (250 µM) in MeCN.

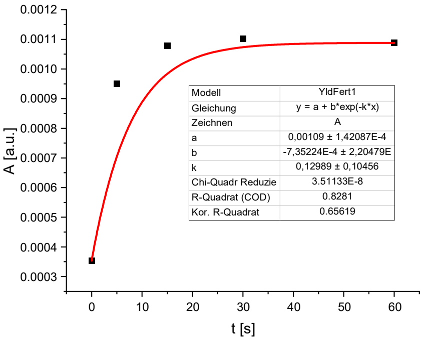

Figure S32: Second order reaction constant calculation for **1**+**11**.

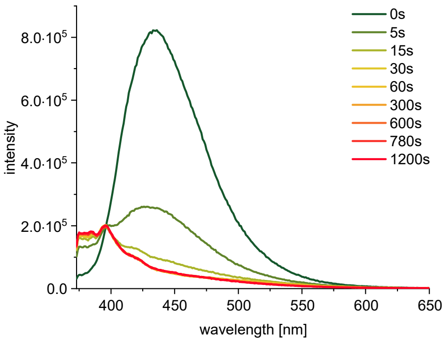

Figure S33: Time-dependent fluorescence of the “photoclick” reaction of **1** (25 µM) and **11** (250 µM) in MeCN.

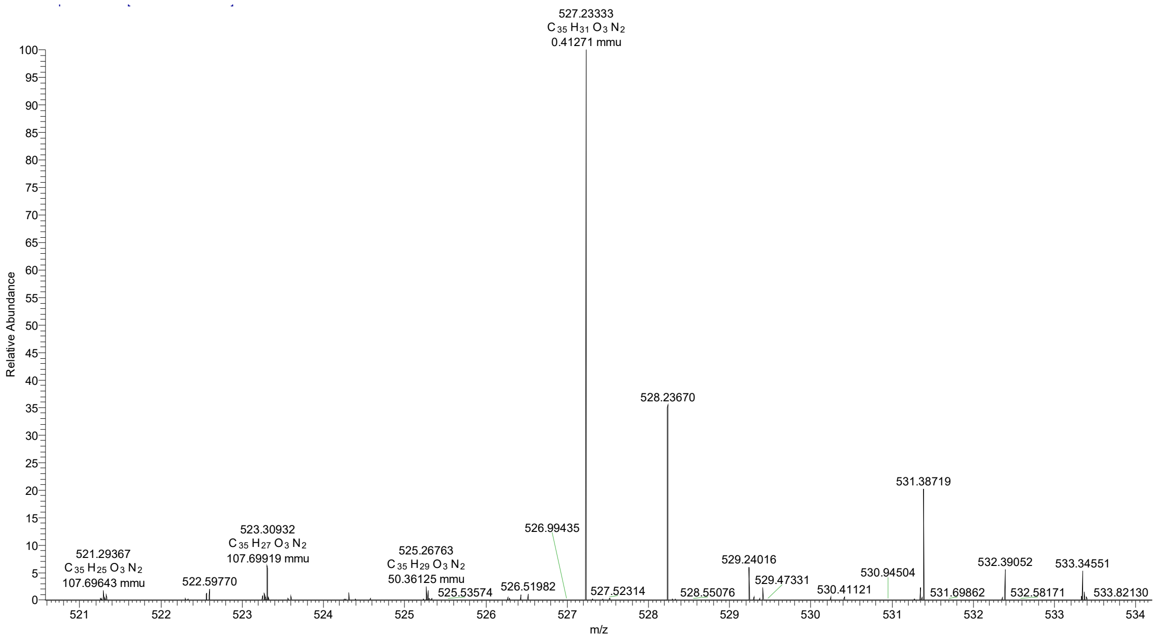

Figure S34: HR-MS (ESI) of product **17**; calculated: $C_{35}H_{30}N_2O_3$ $[M]^+$: 526.22564, measured $[M+H]^+$: 527.23333.

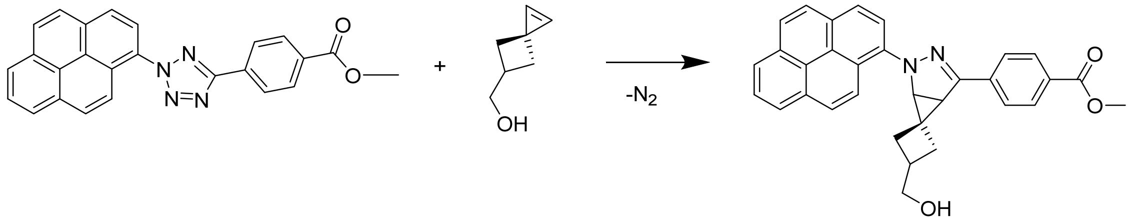

1 **10** **16**

Scheme S5: “Photoclick” reaction of **1** and **10** to **16**.

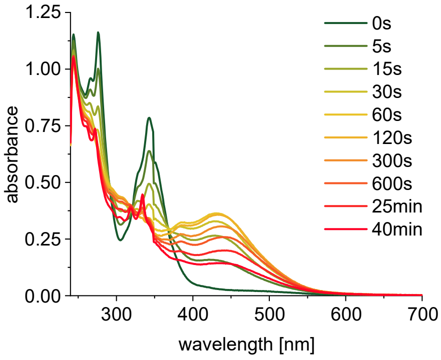

Figure S35: Time-dependent UV/Vis absorbance of the “photoclick” reaction between **1** (25 µM) and **10** (250 µM) in MeCN.

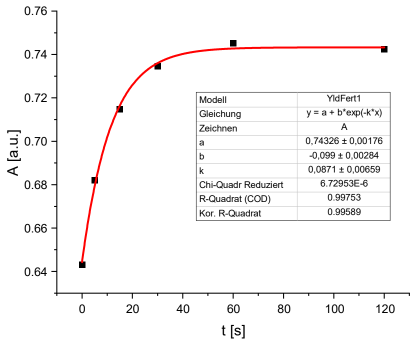

Figure S36: Second order reaction constant calculation for **1**+**10**.

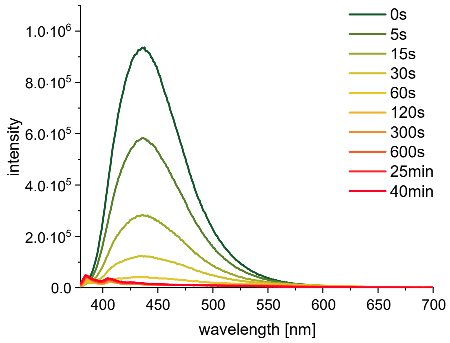

Figure S37: Time-dependent fluorescence of the “photoclick” reaction of **1** (25 µM) and **10** (250 µM) in MeCN.

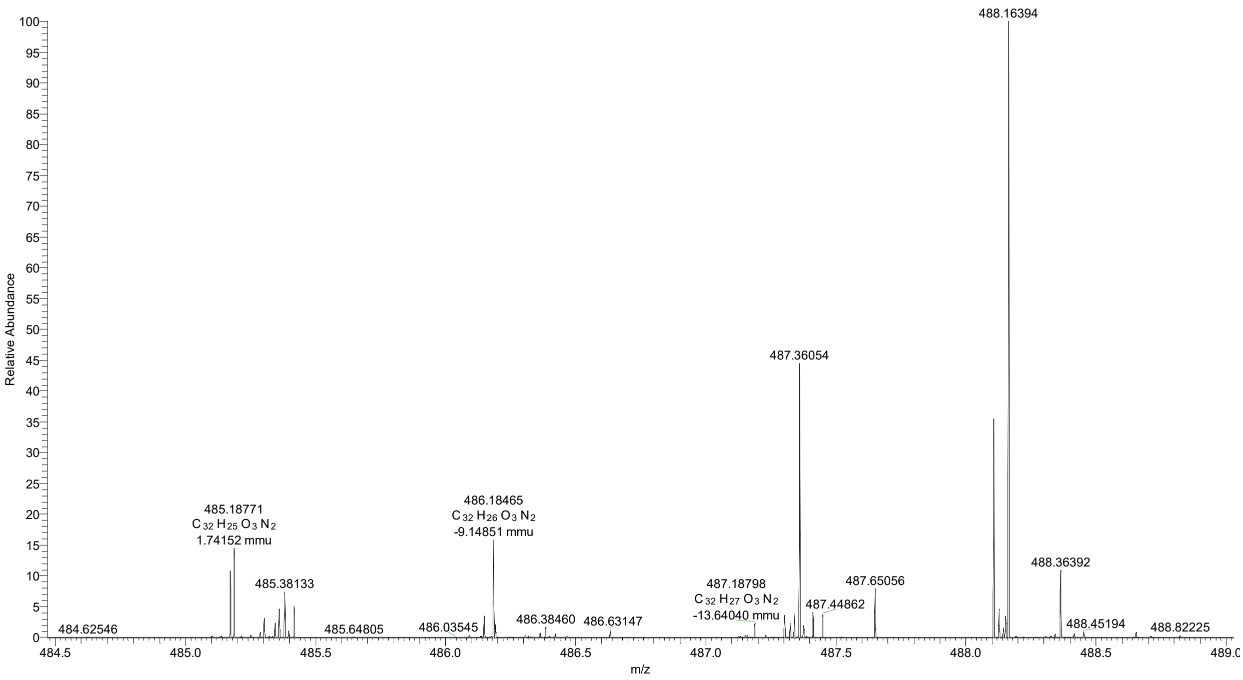

Figure S38: HR-MS (ESI) of the product **16**; calculated: $C_{32}H_{26}N_2O_3$ $[M]^+$: 486.19434, measured $[M-H]^+$: 485.18771.

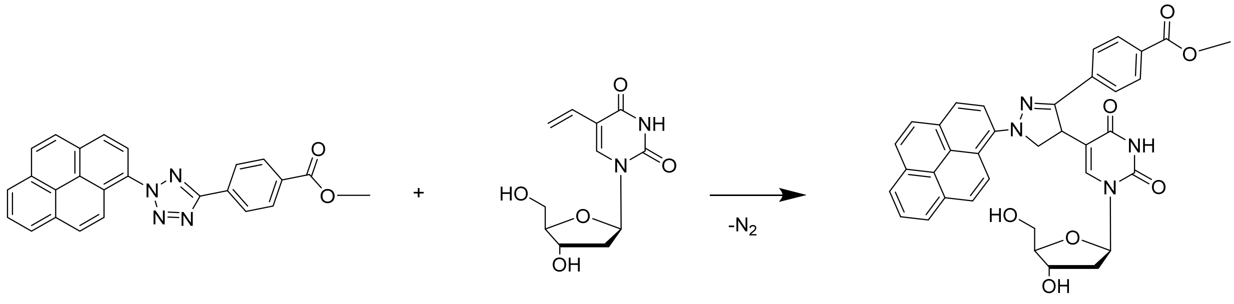

$C_{36}H_{30}N_4O_7$
630,21145

1 **12** **18**

Scheme S6: "Photoclick" reaction of **1** and **12** to **18**.

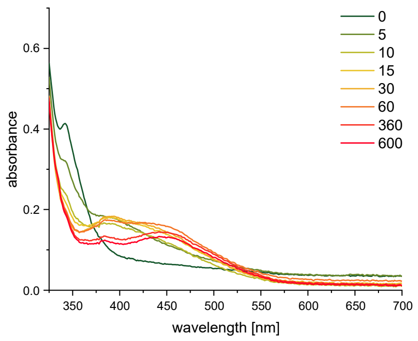

Figure S39: Time-dependent UV/Vis absorbance of the "photoclick" reaction of **1** (25 µM) and **12** (250µM) in MeCN.

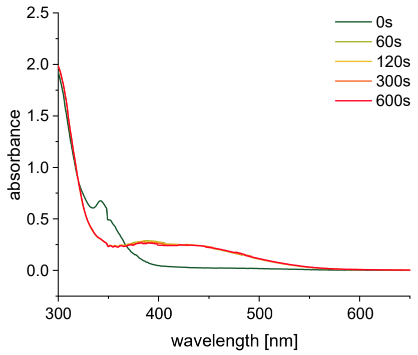

Figure S40: Time-dependent UV/Vis absorbance of the "photoclick" reaction of **1** (25 µM) and **12** (250µM) in MeCN. Irradiation for only 60 s.

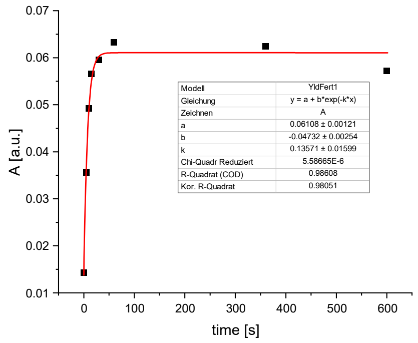

Figure S41: Second order reaction constant calculation for **1+12**.

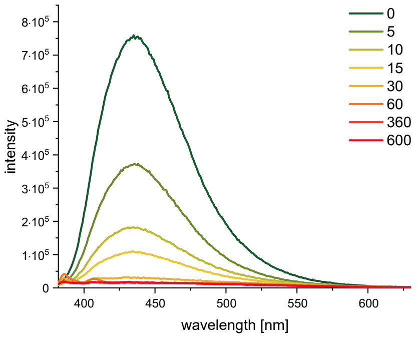

Figure S42: Time-dependent fluorescence of the “photoclick” reaction of **1** (25 µM) and **12** (250 µM), λ_{exc}=355 nm.

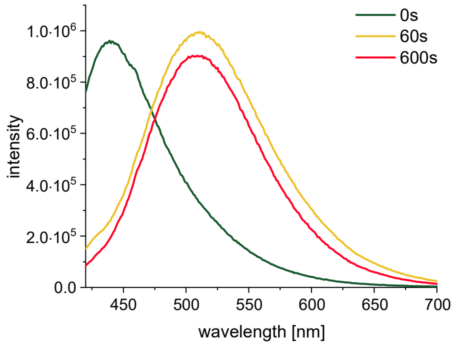

Figure S43: Time-dependent fluorecence of the “photoclick” reaction of **1** (25 µM) and **12** (250 µM), λ_{exc}=405 nm.

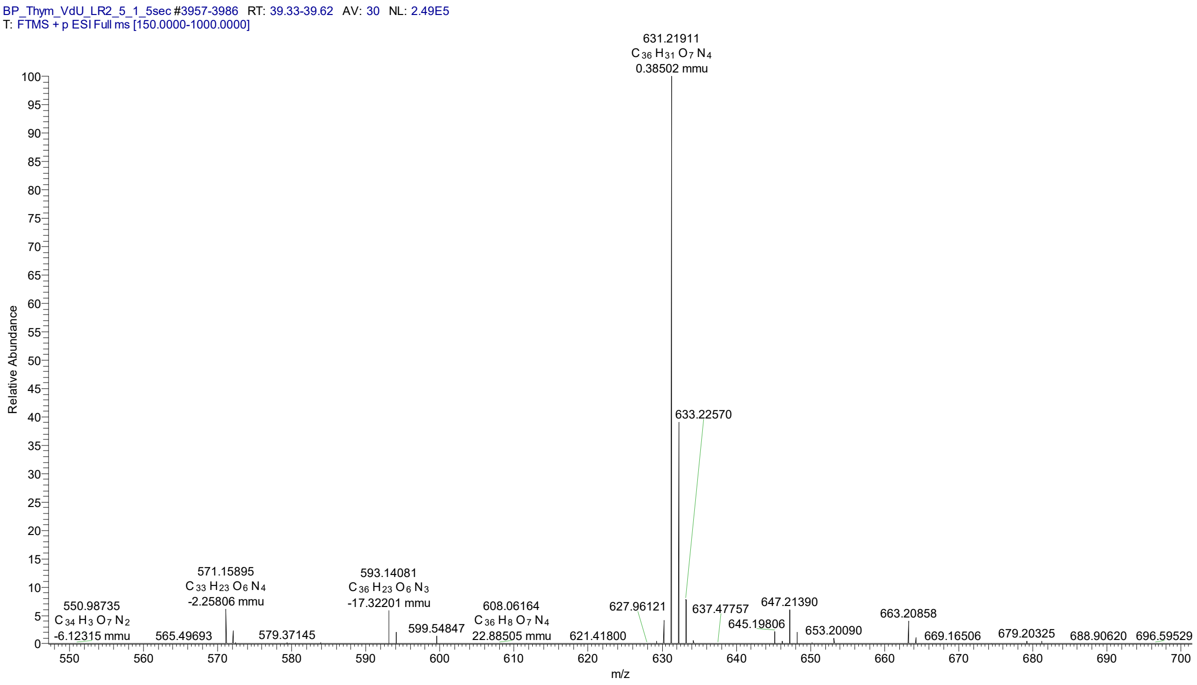

Figure S44: HR-MS (ESI) of photoclick-product **18**; calculated: $C_{36}H_{30}N_4O_7$ $[M]^+$: 630.21145; measured $[M+H]^+$: 631.21911.

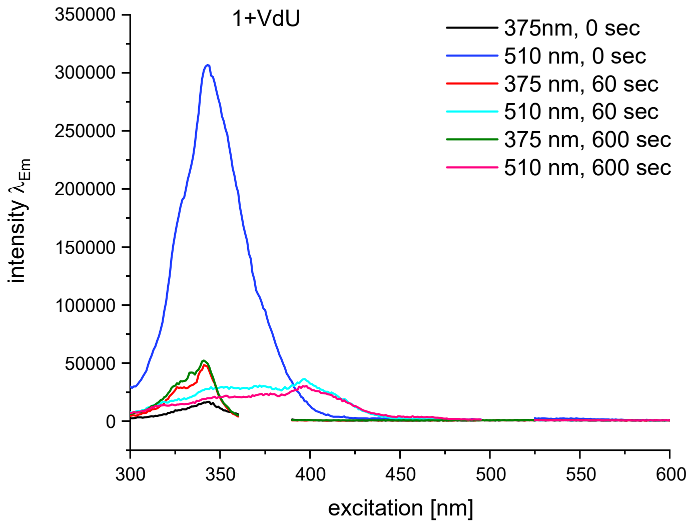

Figure S45: Time-dependent excitation spectra of the "photoclick" reaction of **1** (25 µM) and **12** (250 µM) in MeCN, λ_{em}=375 and 510 nm.

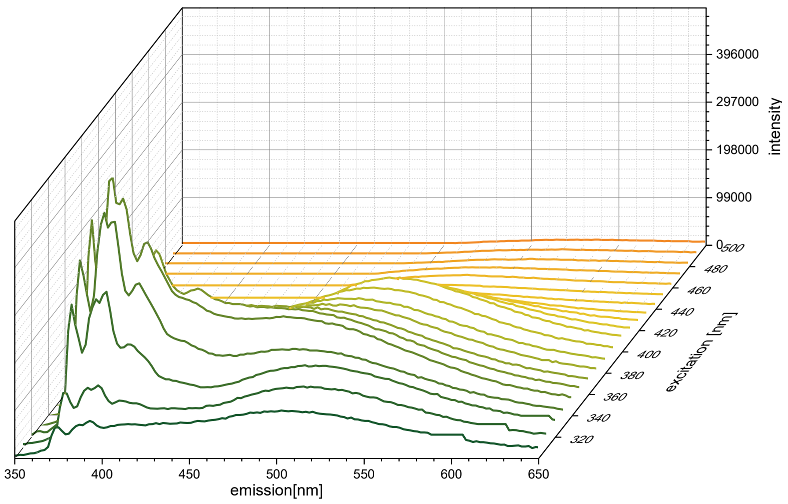

Figure S46: 3D fluorescence after the "photoclick" reaction of **1** (25 µM) and **12** (250 µM) in MeCN.

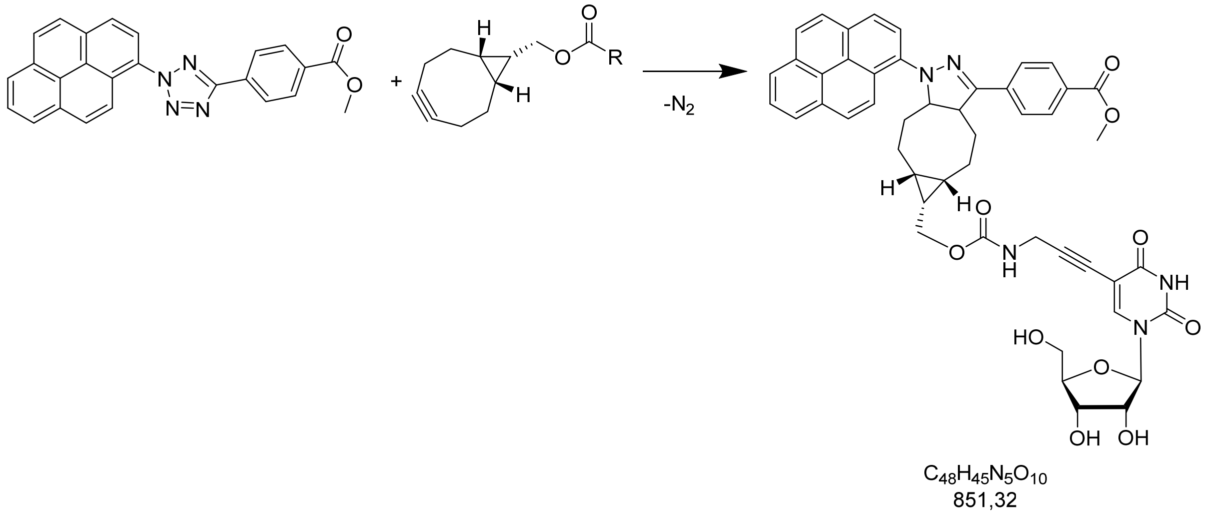

1 **14** **20**

Scheme S7: "Photoclick" reaction of **1** and **14** to **20**.

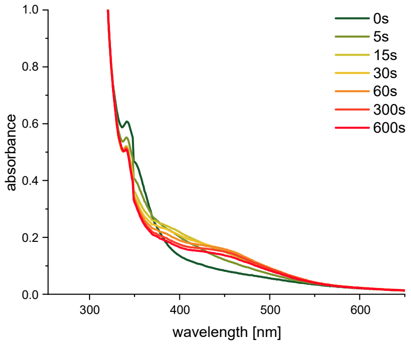

Figure S47: Time-dependent UV/Vis absorbance of the "photoclick" reaction of **1** (25 µM) and **14** (250 µM) in MeCN.

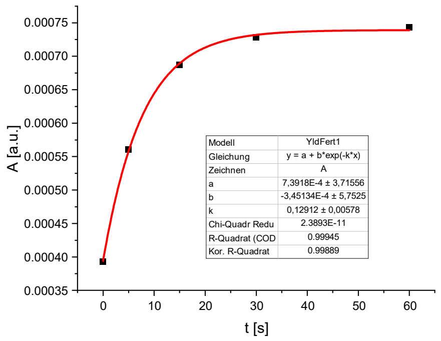

Figure S48: Second order reaction constant calculation for **1**+**14**.

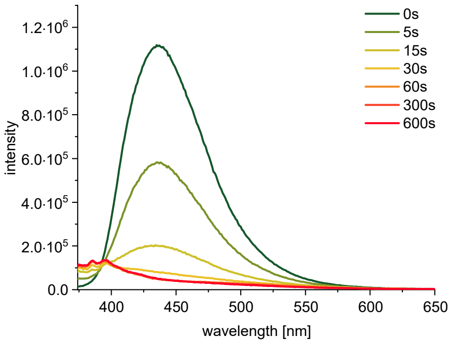

Figure S49: Time-dependent fluorescence of the “photoclick” reaction of **1** (25 µM) and **14** (250 µM) in MeCN.

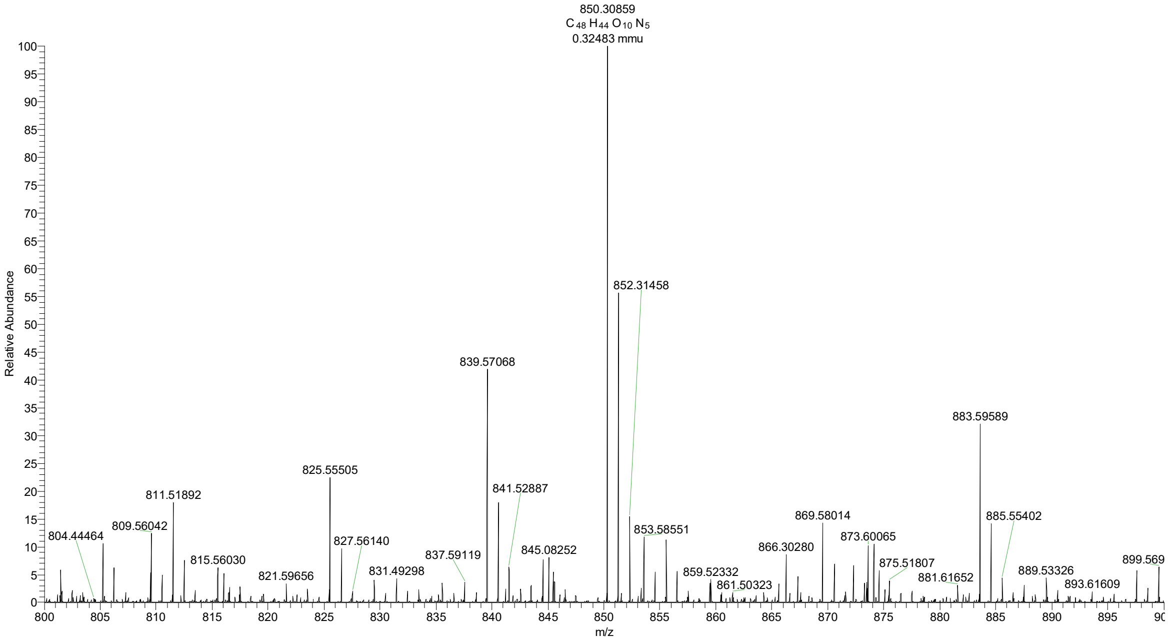

Figure S50: HR-MS (ESI) of the product **20**; calculated: $C_{48}H_{45}N_5O_{10}$ [M]$^+$: 851.31664; measured [M-H]$^+$: 850.30859.

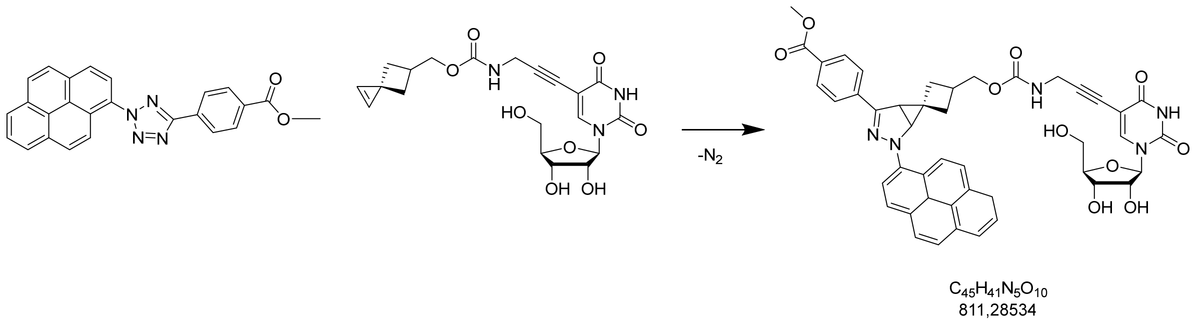

1 **13** **18**

Scheme S8: "Photoclick" reaction of **1** and **13** to **18**.

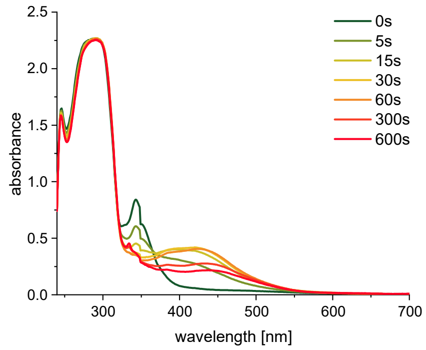

Figure S51: Time-dependent UV/Vis absorbance "photoclick" reaction of **1** (25 µM) and **13** (250 µM) in MeCN.

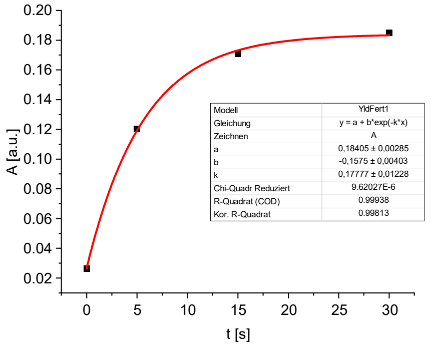

Modell	YldFert1
Gleichung	y = a + b*exp(-k*x)
Zeichnen	A
a	0,18405 ± 0,00285
b	-0,1575 ± 0,00403
k	0,17777 ± 0,01228
Chi-Quadr Reduziert	9.62027E-6
R-Quadrat (COD)	0.99938
Kor. R-Quadrat	0.99813

Figure S52: Second order reaction constant calculation for **1+13**.

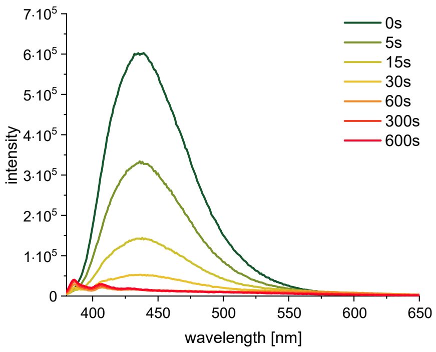

Figure S53: Time-dependent fluorescence of the "photoclick" reaction of **1** (25 µM) and **13** (250 µM) in MeCN.

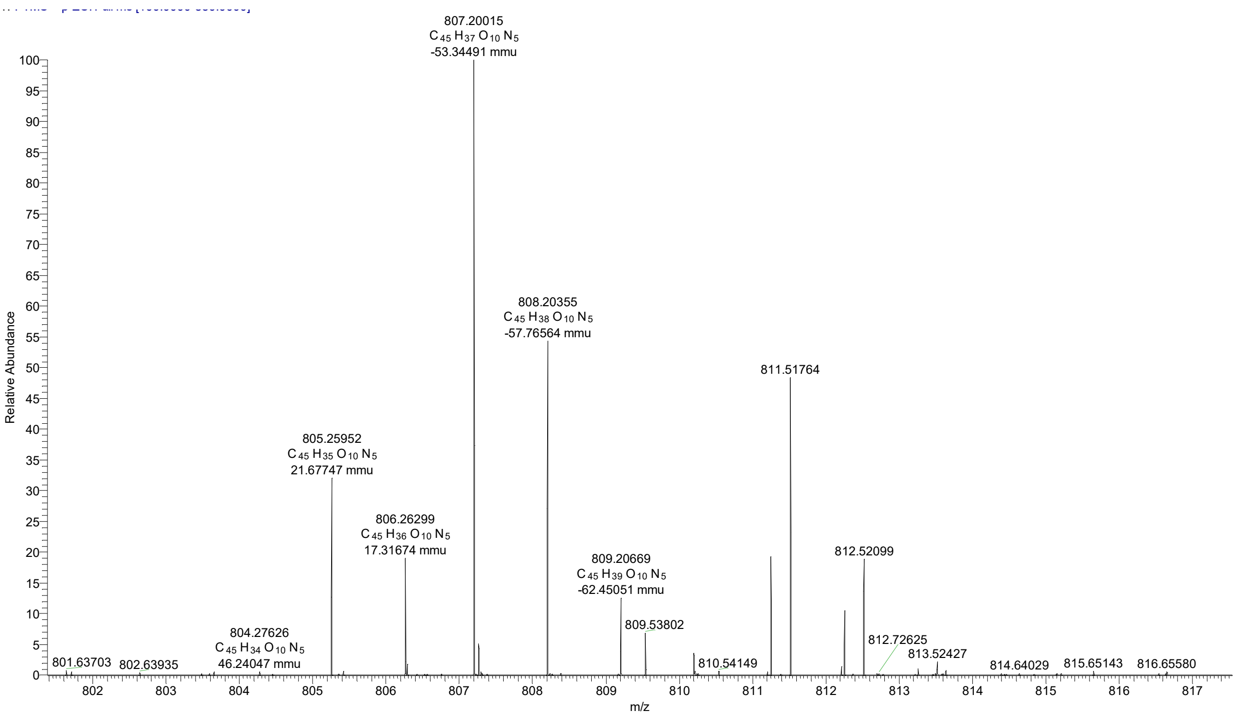

Figure S54: HR-MS (ESI) of the product **19**; calculated: $C_{45}H_{41}N_5O_{10}$ $[M]^+$: 811.28534, measured $[M]^+$: 811.51764.

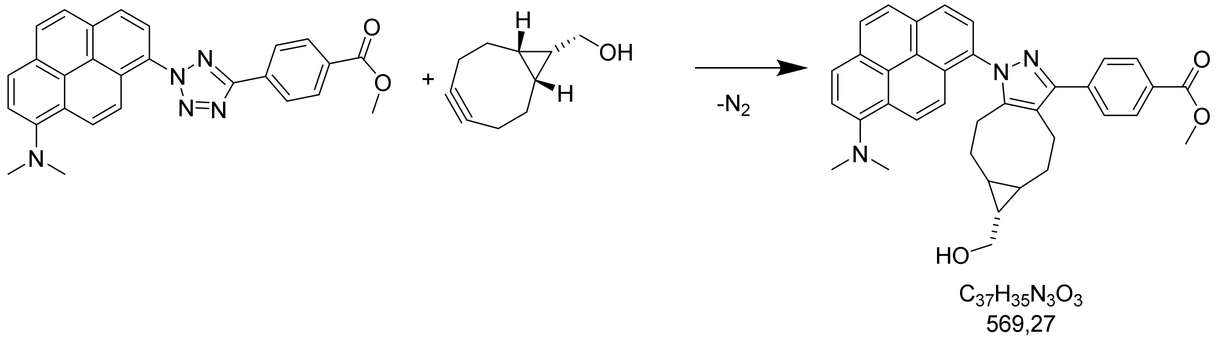

2 **11** **23**

Scheme S9: **"**Photoclick" reaction of **2** and **11** to **23**.

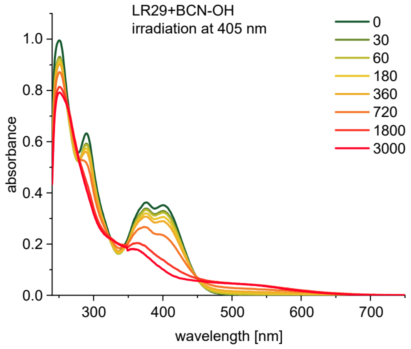

Figure S55: Time-dependent UV/Vis absorbance of the "photoclick" reaction of **2** (25 µM) and **11** (250 µM) in MeCN.

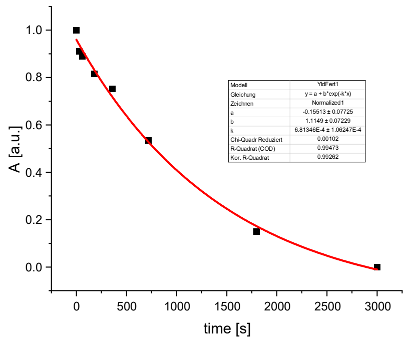

Figure S56: Second order reaction constant calculation for **2**+**11**.

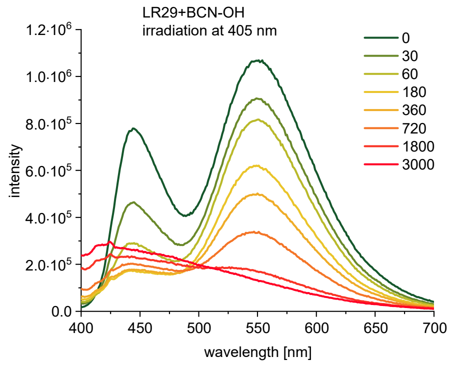

Figure S57: Time-dependent fluorescence of the "photoclick" reaction of **2** (25 µM) and **11** (250 µM) in MeCN, λ_{exc}=355 nm.

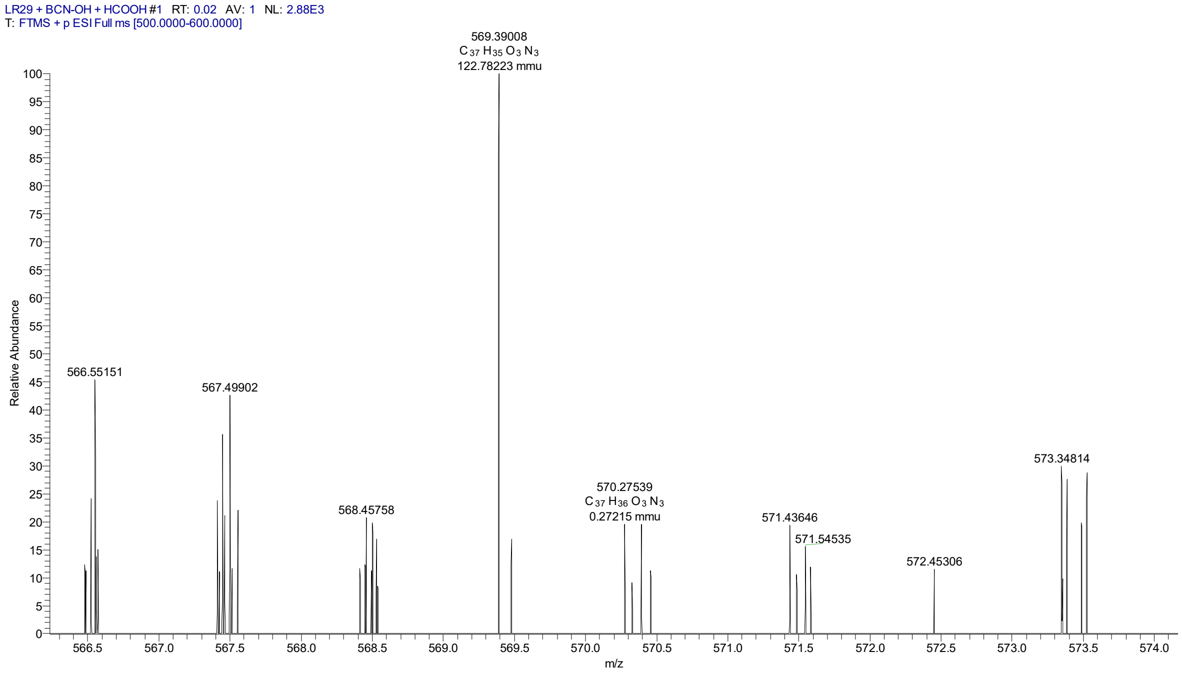

Figure S58: HR-MS (ESI) of product **23**; calculated: $C_{34}H_{32}N_3O_3$ [M]$^+$: 569.26784; measured [M+H]$^+$: 570.27539.

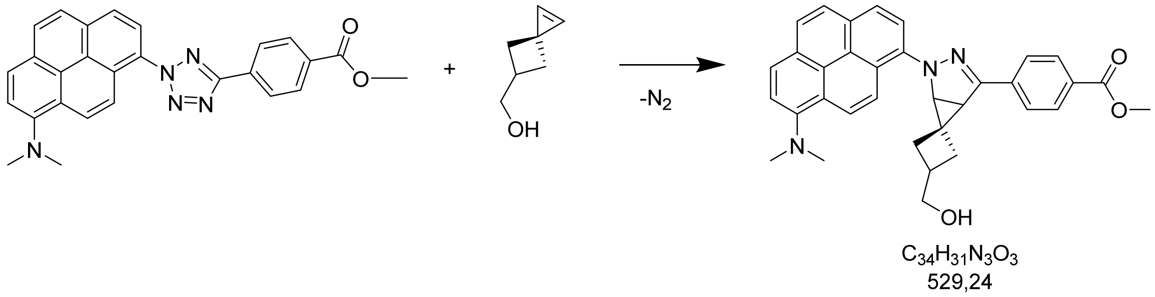

2 **10** **22**

Scheme S10: “Photoclick” reaction of **2** and **10** to **20**.

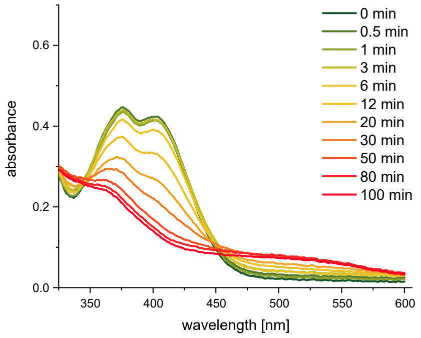

Figure S59: Time-dependent UV/Vis absorbance of the “photoclick” reaction of **2** (25 µM) and **10** (250 µM) in MeCN.

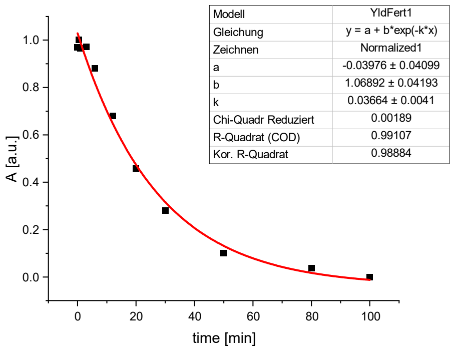

Figure S60: Second order reaction constant calculation for **2**+**10**.

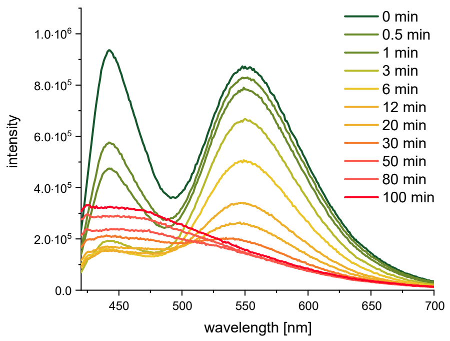

Figure S61: Time-dependent fluorescence of the “photoclick” reaction of **2** (25 µM) and **10** (250 µM) in MeCN, λ_{exc}=355 nm.

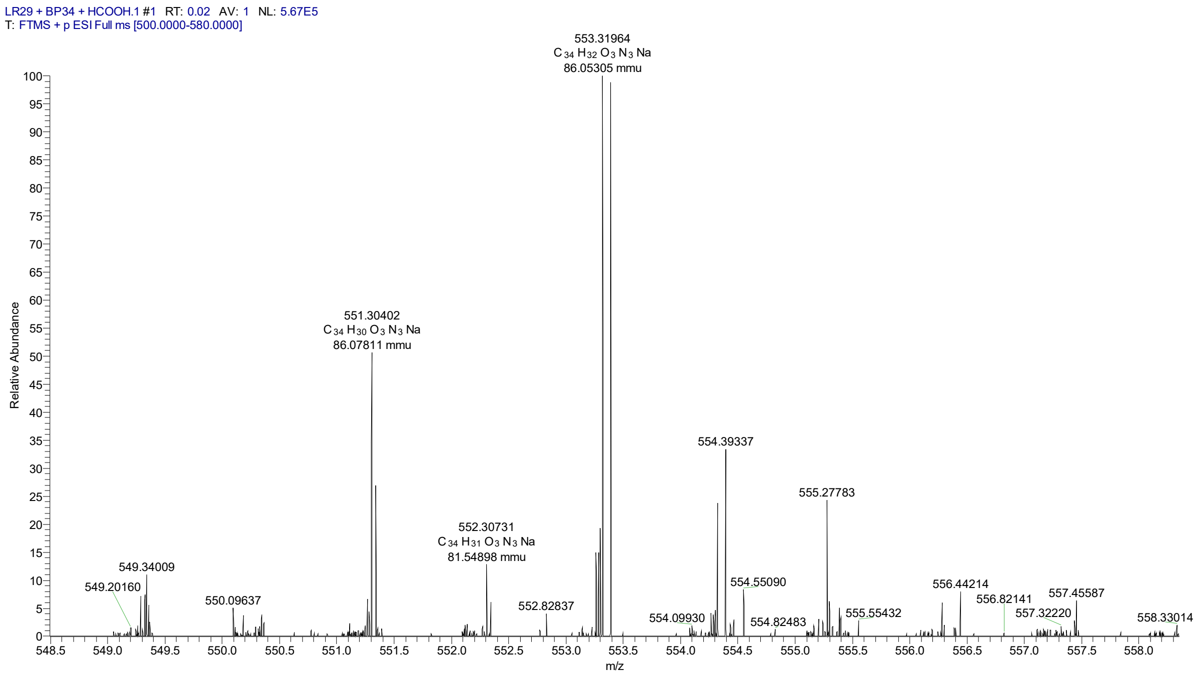

Figure S62: HR-MS (ESI) of product **22**; calculated: $C_{34}H_{32}N_3O_3$ $[M]^+$: 529.23654; measured $[M-H+Na]^+$: 551.30402.

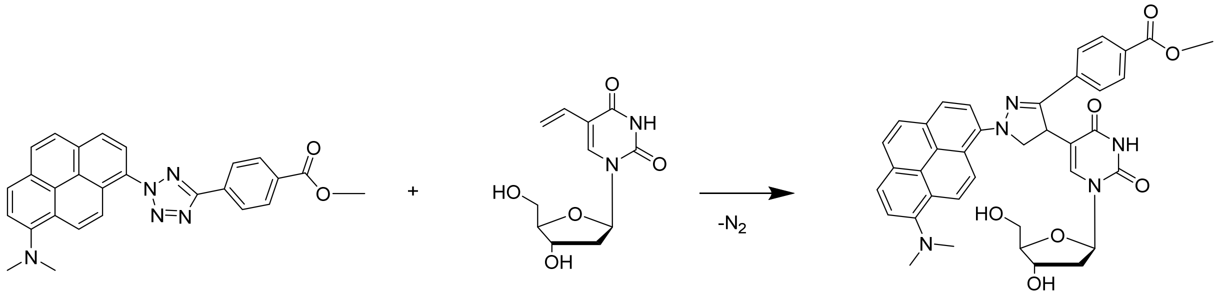

$C_{38}H_{35}N_5O_7$
673,25

2 **12** **24**

Scheme S11: **"**Photoclick" experiment of **2** and **12** to **24**.

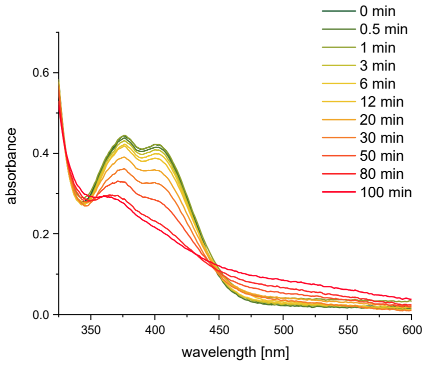

Figure S63: Time-dependent UV/Vis absorbance of the "photoclick" reaction of **2** (25 µM) and **12** (250 µM) in MeCN.

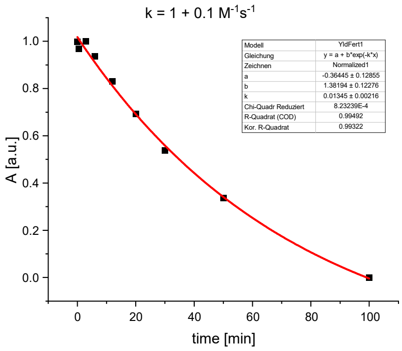

Figure S64: Second order reaction constant calculation for **2**+**12**.

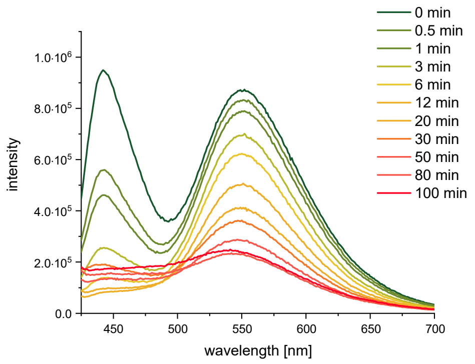

Figure S65: Time-dependent fluorescence of the “photoclick” reaction of **2** (25 µM) and **12** (250 µM) in MeCN, λ_{exc}=405 nm.

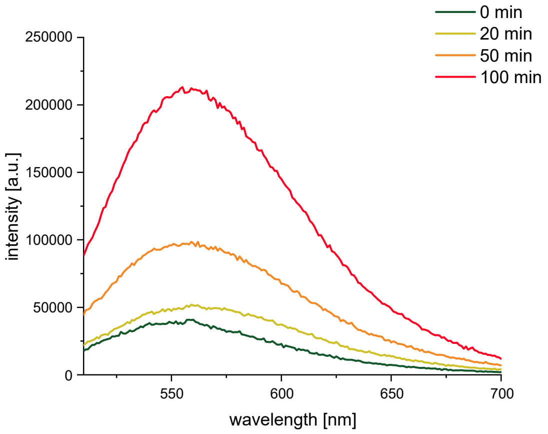

Figure S65b): Time-dependent fluorescence of the “photoclick” reaction of **2** (25 µM) and **12** (250 µM) in MeCN, λ_{exc}=480 nm.

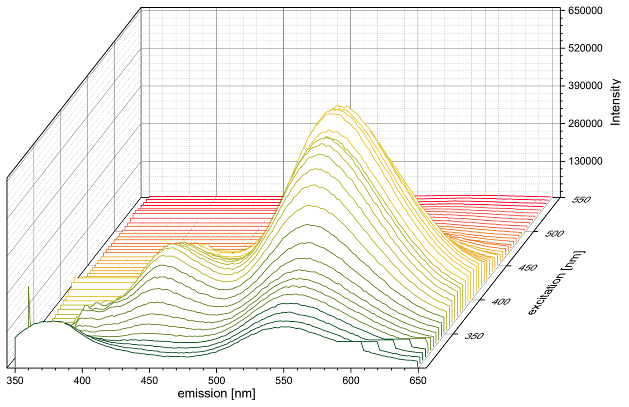

Figure S66: Time-dependent 3D fluorescence before the “photoclick” reaction of **2** (25 µM) and **12** (250 µM) in MeCN.

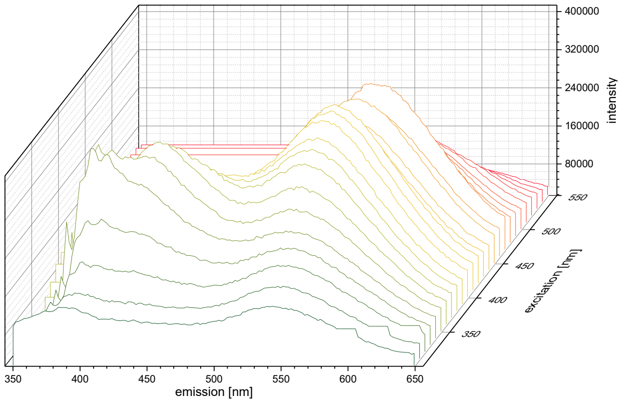

Figure S67: Time-dependent 3D fluorescence after the “photoclick” reaction of **2** (25 µM) and **12** (250 µM) in MeCN

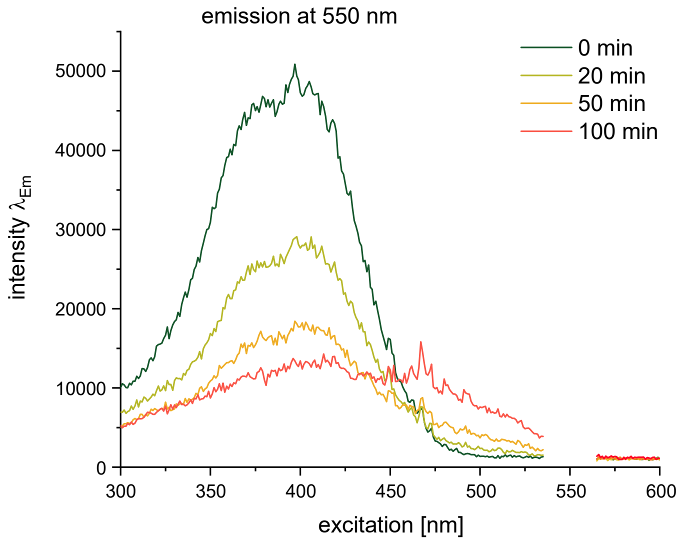

Figure S68: Time-dependent excitation spectra of the “photoclick” reaction of **2** (25 µM) and **12** (250 µM) in MeCN, λ_{em}=550 nm.

.

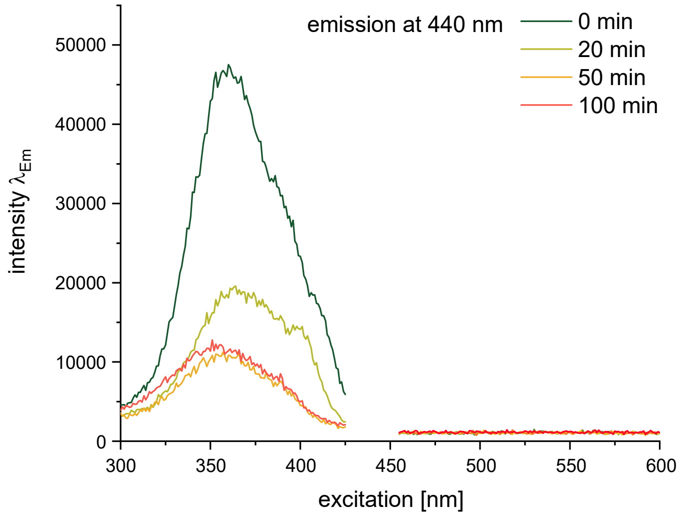

Figure S69: Time-dependent excitation spectra of the “photoclick” reaction of **2** (25 µM) and **12** (250 µM) in MeCN, λ_{em}=440 nm.

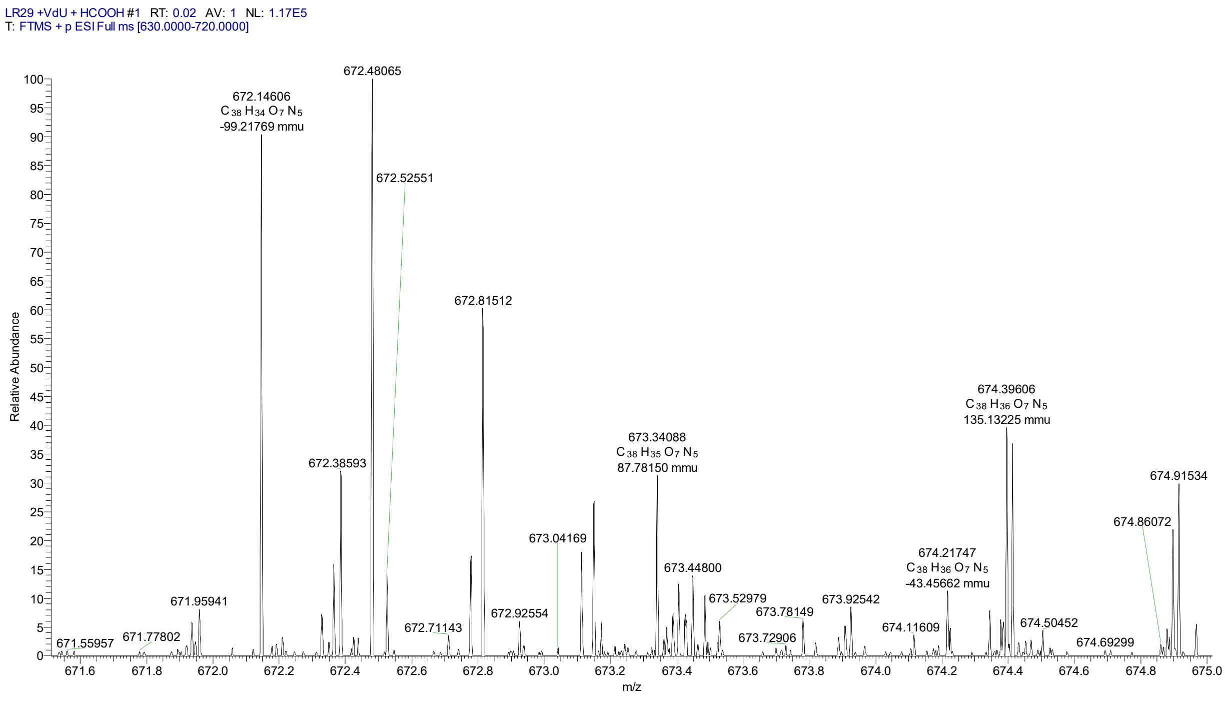

Figure S70: HR-MS (ESI) of product **24**; m/z calculated: $C_{38}H_{34}N_5O_7$ $[M]^+$: 673.25365; measured $[M-H]^+$: 672.14606.

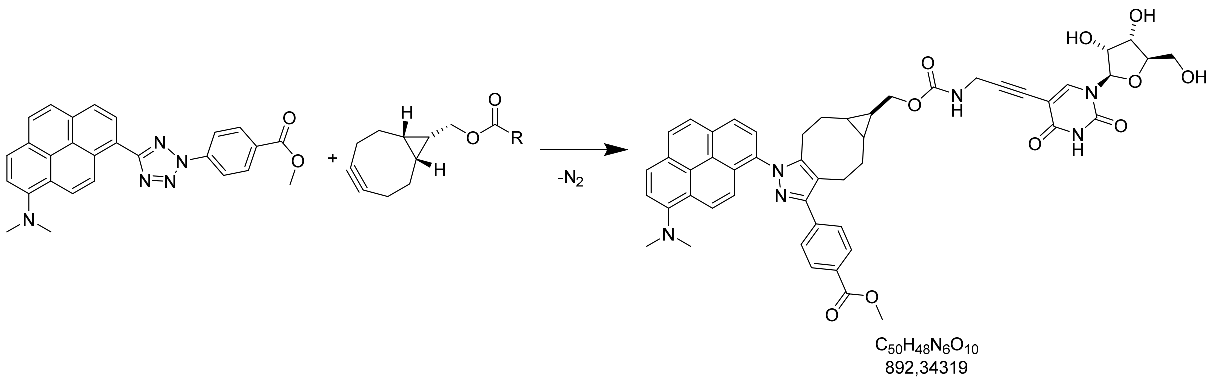

2 **14** **26**

Scheme S12: ""Photoclick" reaction of **2** and **14** to **26**.

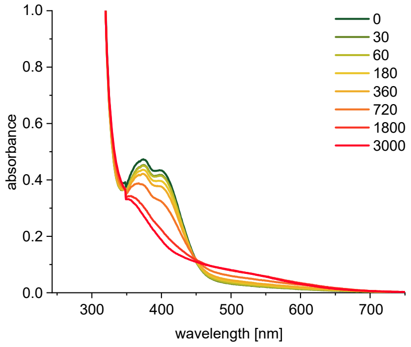

Figure S71: Time-dependent UV/Vis absorbance of the "photoclick" reaction of **2** (25 µM) and **14** (250 µM) in MeCN.

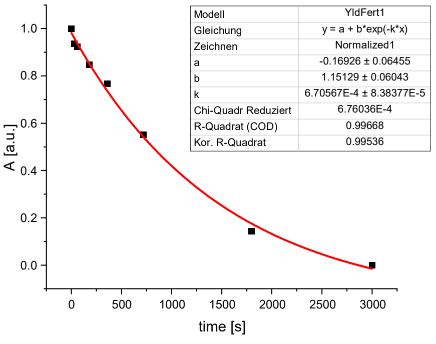

Figure S72: Second order rate constant calculation for **2+14**.

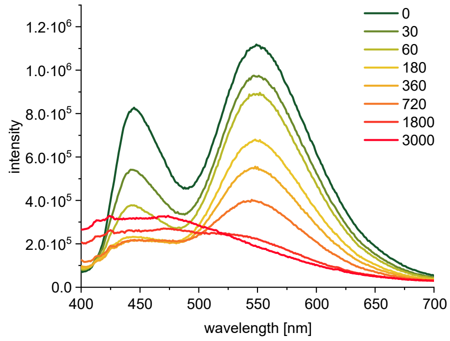

Figure S73: Time-dependent fluorescence of the "photoclick" reaction of **2** (25 µM) and **14** (250 µM) in MeCN, λ_{exc}=355 nm.

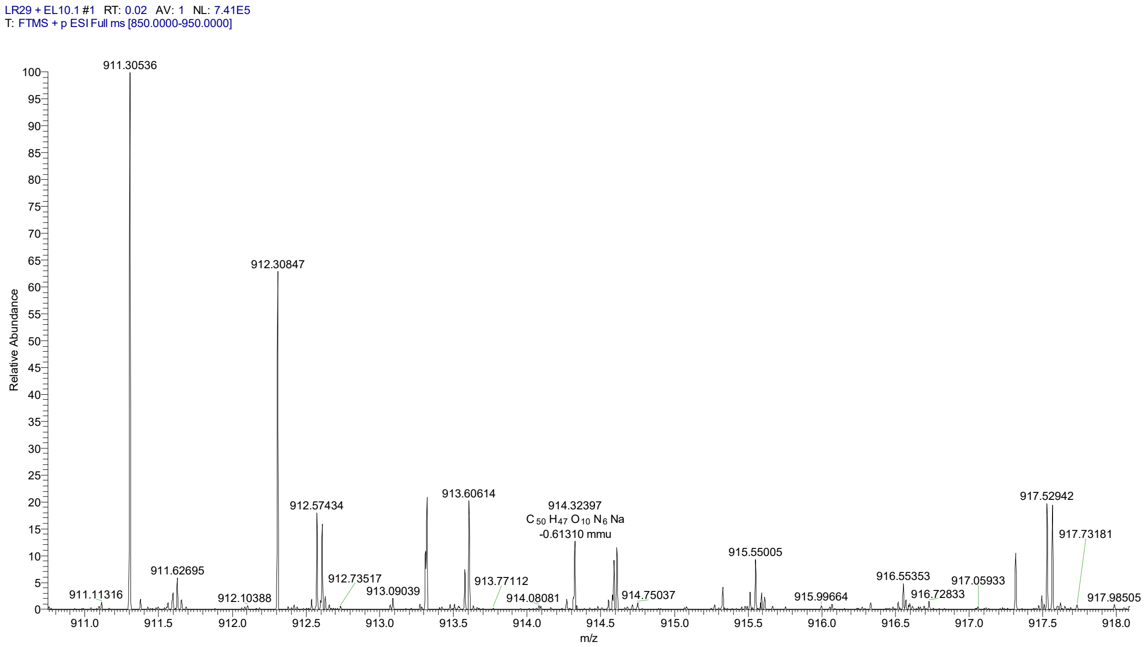

Figure S74: HR-MS (ESI) of product **26**. calculated: $C_{34}H_{32}N_3O_3$ [M]+: 892.34319; measured [M-H+Na]$^+$: 914.32397.

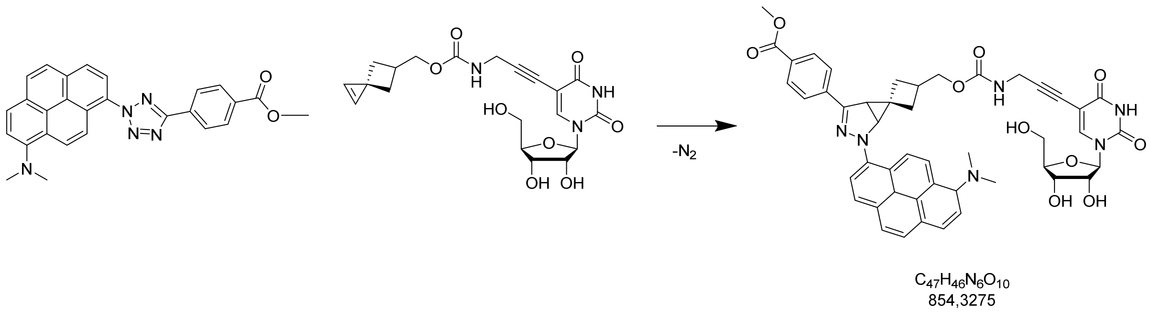

2 **13** **25**

Scheme S13: “Photoclick” reaction of **2** and **13** to product **25**.

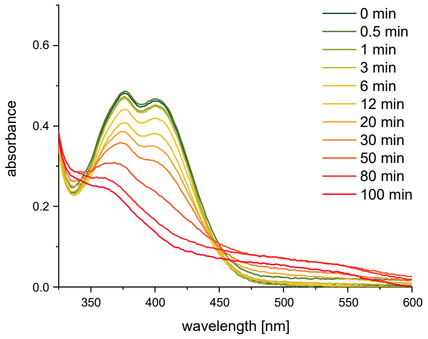

Figure S75: Time-dependent UV/Vis absorbance of the “photoclick” reaction of **2** (25 µM) and **13** (250 µM) in MeCN.

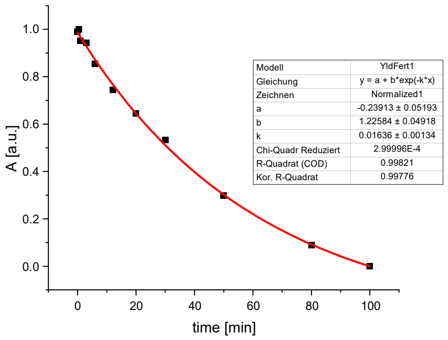

Figure S76: Second order rate constant calculation for **2**+**13**.

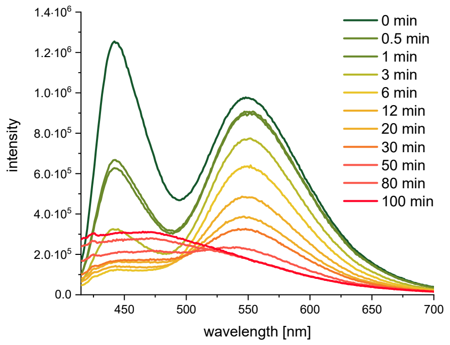

Figure S77: Time-dependent excitation spectra of the “photoclick” reaction of **2** (25 µM) and **13** (250 µM) in MeCN, λ_{exc}=355 nm.

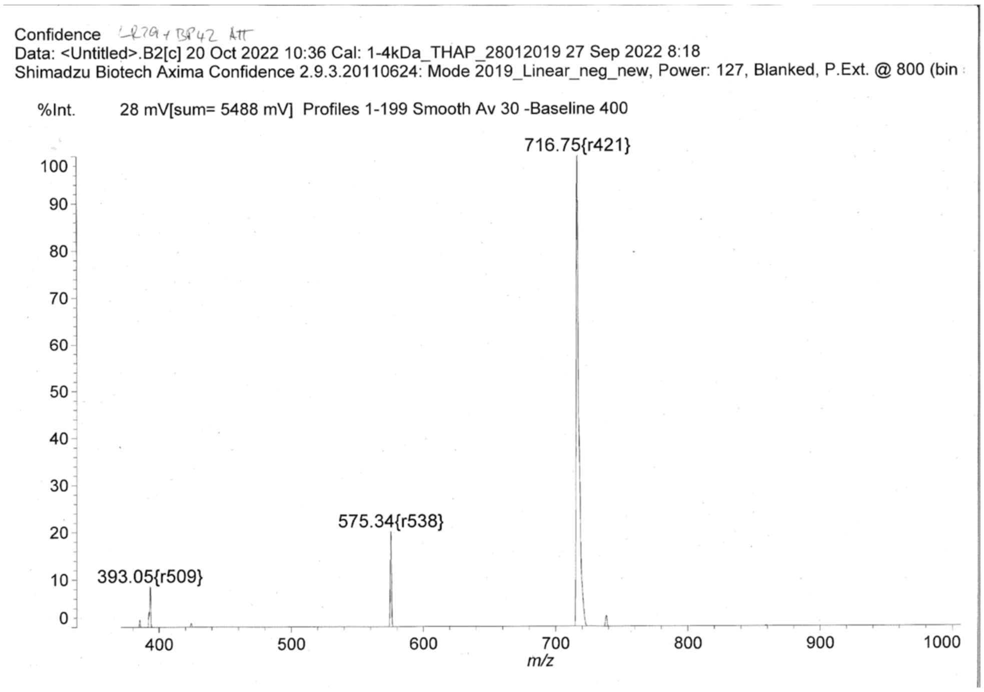

Figure S78: MALDI-TOF-MS of -product **25**; calculated: $C_{47}H_{46}N_6O_{10}$ $[M]^+$: 854.23

calculated: $C_{39}H_{39}N_6O_8$ $[M]^+$: 719.28, measured $[M]^+$: 716.75

calculated: $C_{35}H_{33}N_4O_4$ $[M]^+$: 573.25, measured $[M]^+$: 575.34.

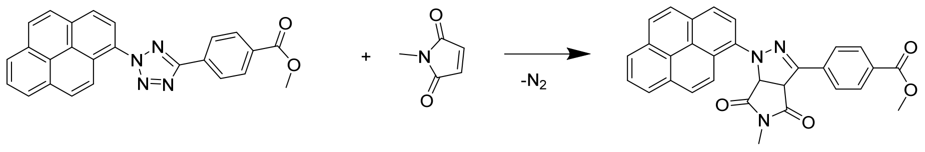

1 **9** **15**

Scheme 14: Photoclick-Experiment of **1+9** to product **15**.

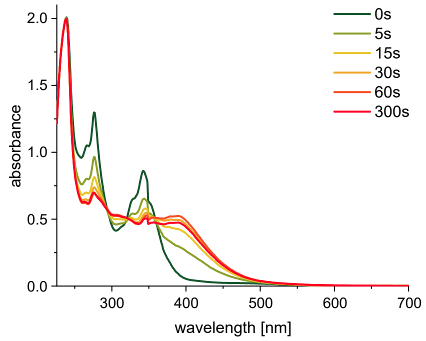

Figure S79. Time-resolved absorbance of the "photoclick" reaction between **1** (25 µM) and **9** (250 µM) in MeCN.

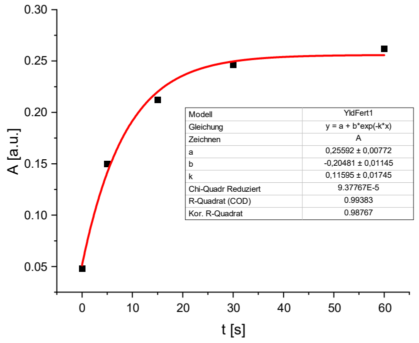

Figure S80. Exponential fit to the absorbance change at λ = 402 nm to calculate the second order reaction constant for **1+9**.

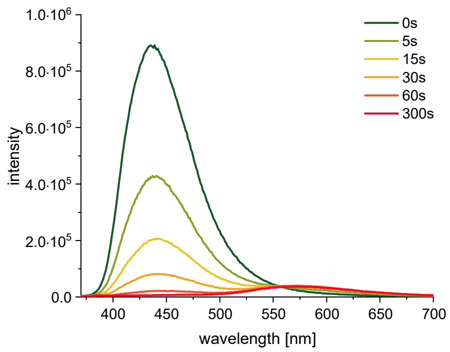

Figure S81. Time-resolved fluorescence of the "photoclick" reaction between **1** (25 µM) and **9** (250 µM) in MeCN

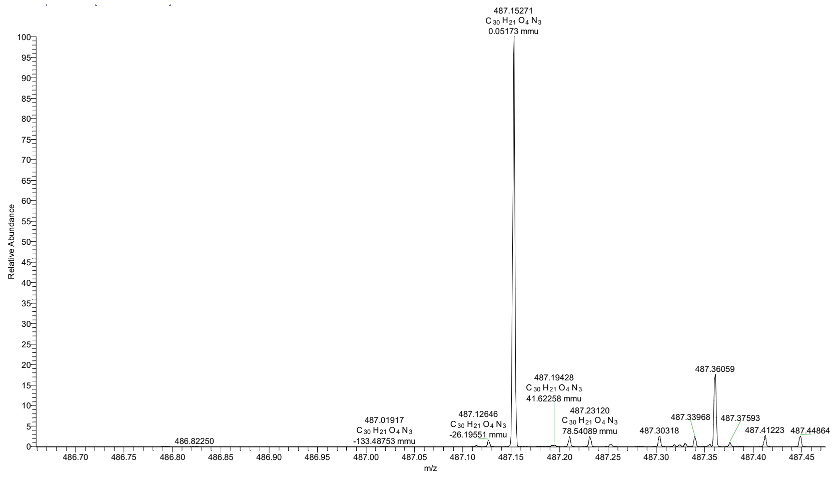

Figure S82. ESI-HRMS analysis of the "photoclick" product **15**; calculated: $C_{30}H_{21}N_3O_4$ $[M]^+$: 487.1532, measured: 487.1527.

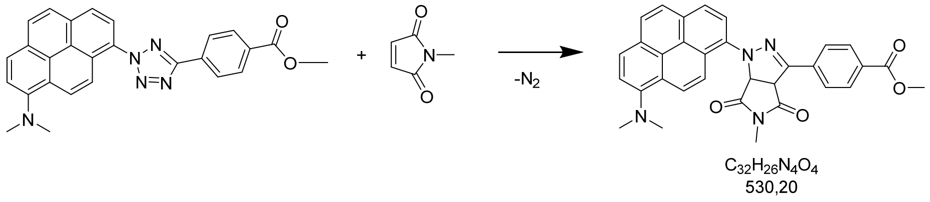

2 **9** **21**

Scheme 15: Photoclick-Experiment of **2+9** to product **21**.

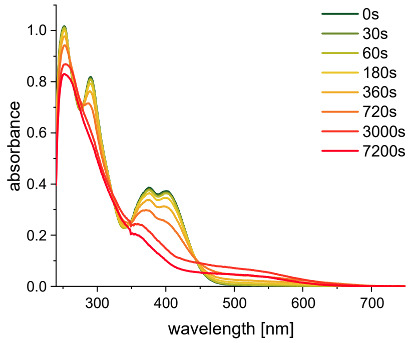

Figure S83. Absorbance spectra of the photoclick-reaction between **2** and **9** in MeCN (25µM 2, 250µM 9).

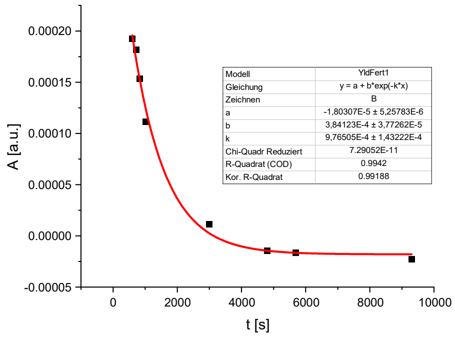

Figure S84. Second order reaction constant calculation for **2**+**9**.

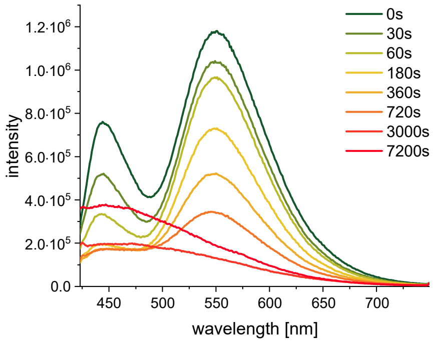

Figure S85. Emission spectrum of photoclick-reaction **21** between **2+9** (25 µM 2, 250 µM 9).

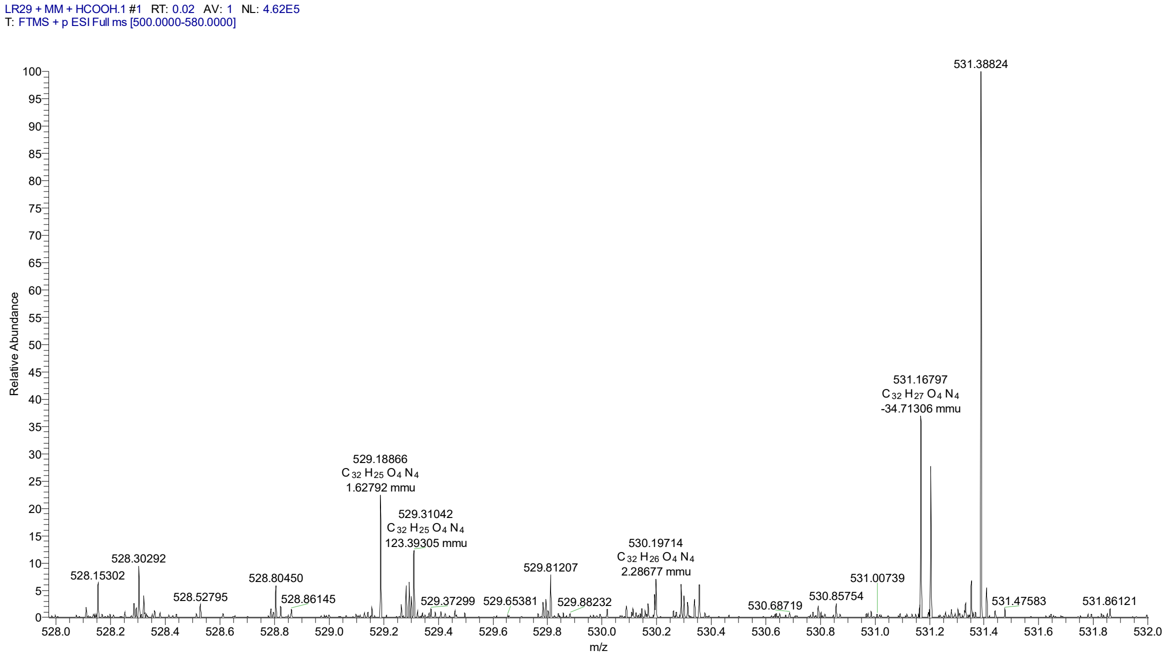

Figure S86. HR-MS (ESI) of photoclick-product **21** between 2+9; calculated: $C_{32}H_{26}N_4O_4$ $[M]^+$: 530.19541; measured $[M\text{-}H]^+$: 529.18866.

d) Irradiation of both tetrazoles without reaction partner

1, irradiated with 405 nm for 600s

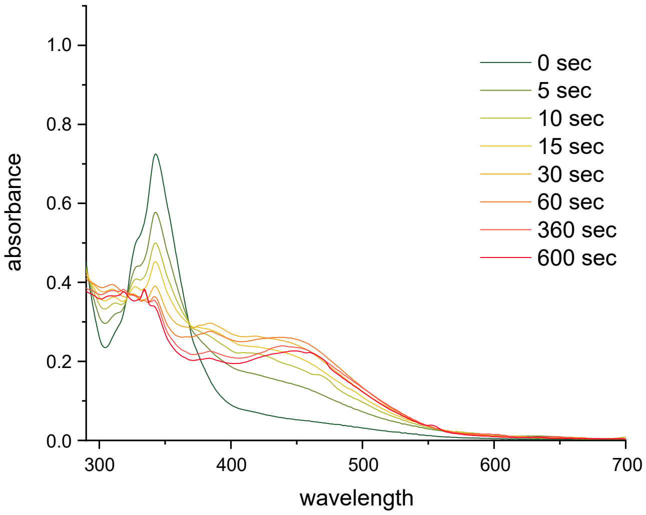

Figure S87a: Irradiation experiment of tetrazole **1** without dipolarophile in a concentration of 25 µM.

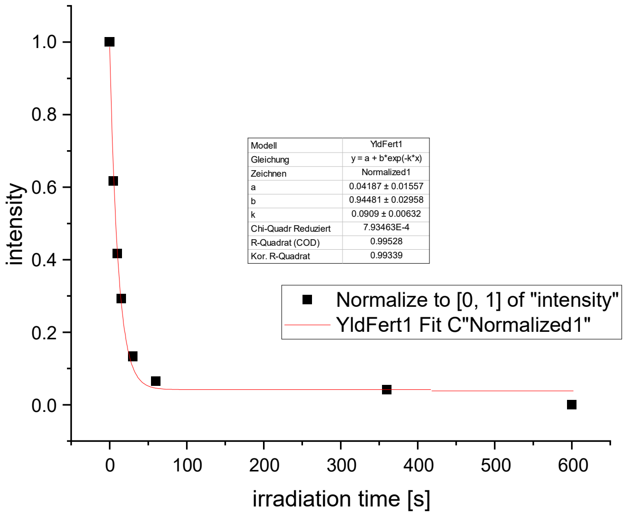

Figure S87b: Second order rate constant calculation for photolysis of **1** (25 µM).

2, irradiated with 405 nm for 80 min

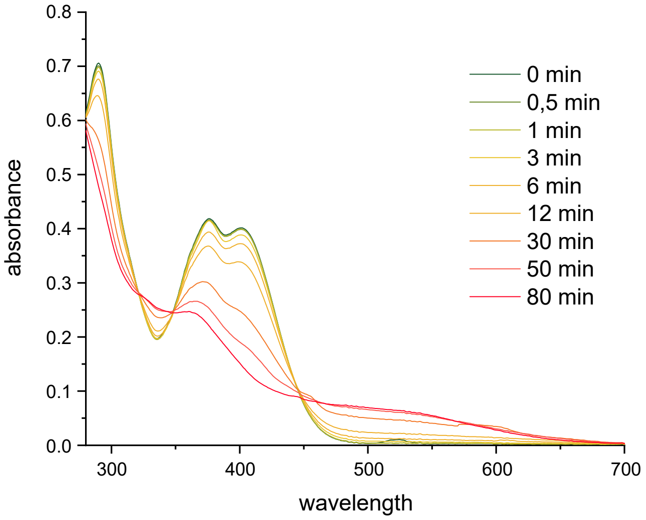

Figure S88a: Irradiation experiment of tetrazole **2** without dipolarophile in a concentration of 25 µM.

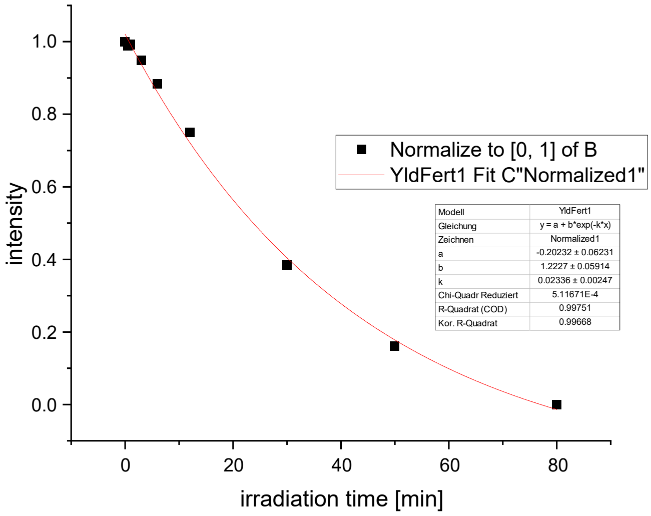

Figure S88b: Second order rate constant calculation for photolysis of **2** (25 µM).

e) RP-HPLC and ESI-HRMS Data

Reversed-phase chromatography (RP-HPLC) measurements were performed with a C18 HPLC column (Phenomenex Luna 5µM, 100 Å, 250 x 2.0 mm) operating at 40 °C. As eluent a gradient from 65 % MeCN/35 % H_2O to 100 % MeCN in 40 min with a flow rate of 0.2 mL·min^{-1} was used.

1 with 9 (MM)

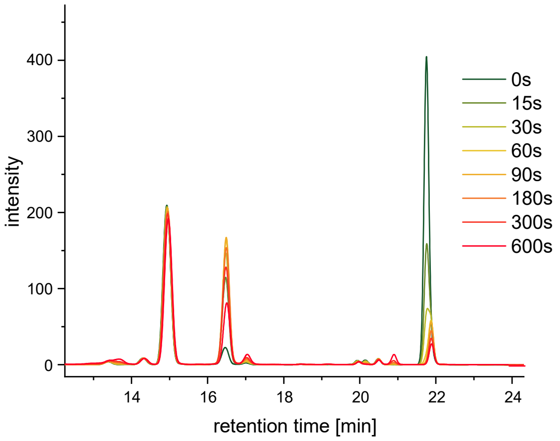

Figure S89: RP-HPLC spectra with 100 µM **1** and 1000 µM **9.**

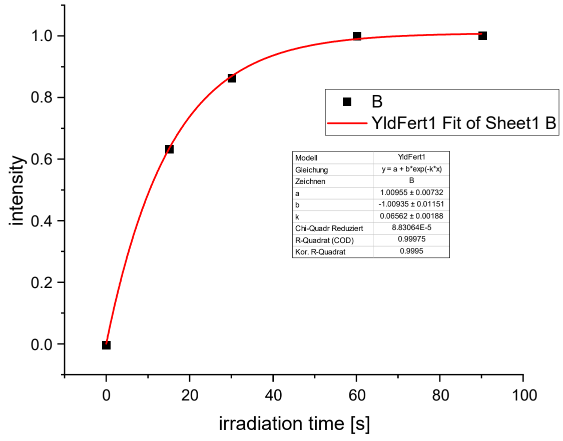

Figure S90a: Second order rate constant calculation for **1+9** product formation.

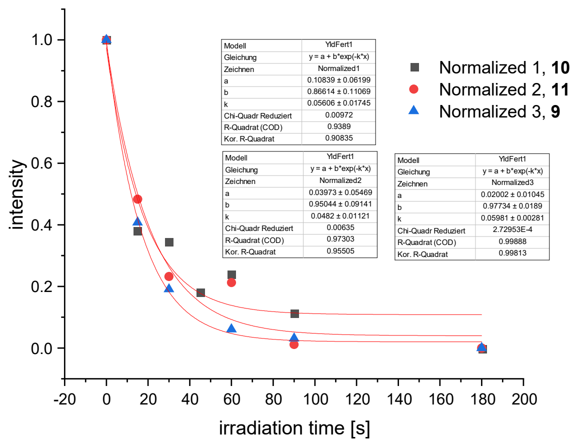

Figure S90b: Second order rate constant calculation for **1**+**9**, **1**+**10**, **1**+**11** photolysis.

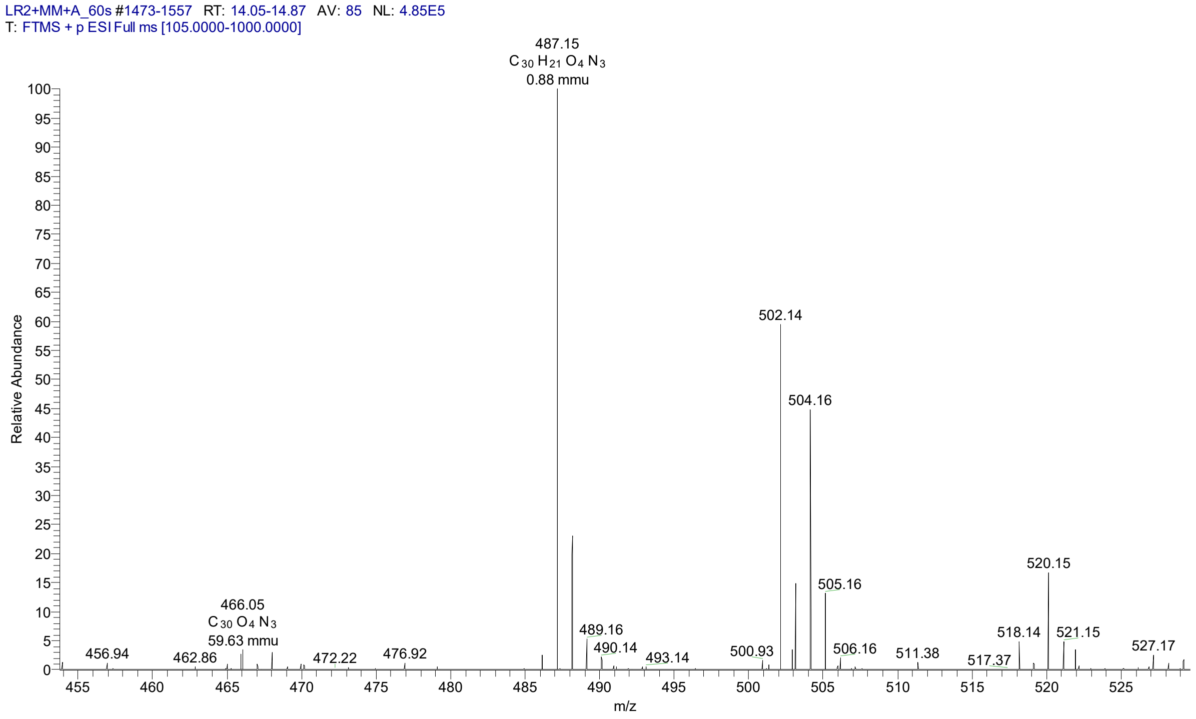

Figure S91: HR-MS (ESI) of product **15**; m/z calculated: $C_{30}H_{21}N_3O_4$ $[M]^+$: 487.15321; measured $[M]^+$: 487.15.

1 with 10 (spirocycle)

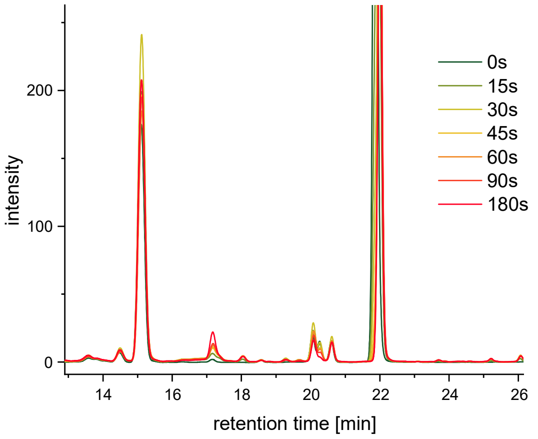

Figure S92: RP-HPLC spectra with 100 µM **1** and 1000 µM **10.**

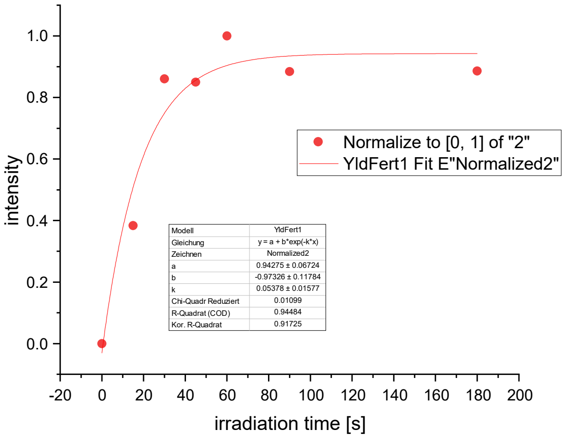

Figure S93: Second order rate constant calculation for **1**+**10** product formation.

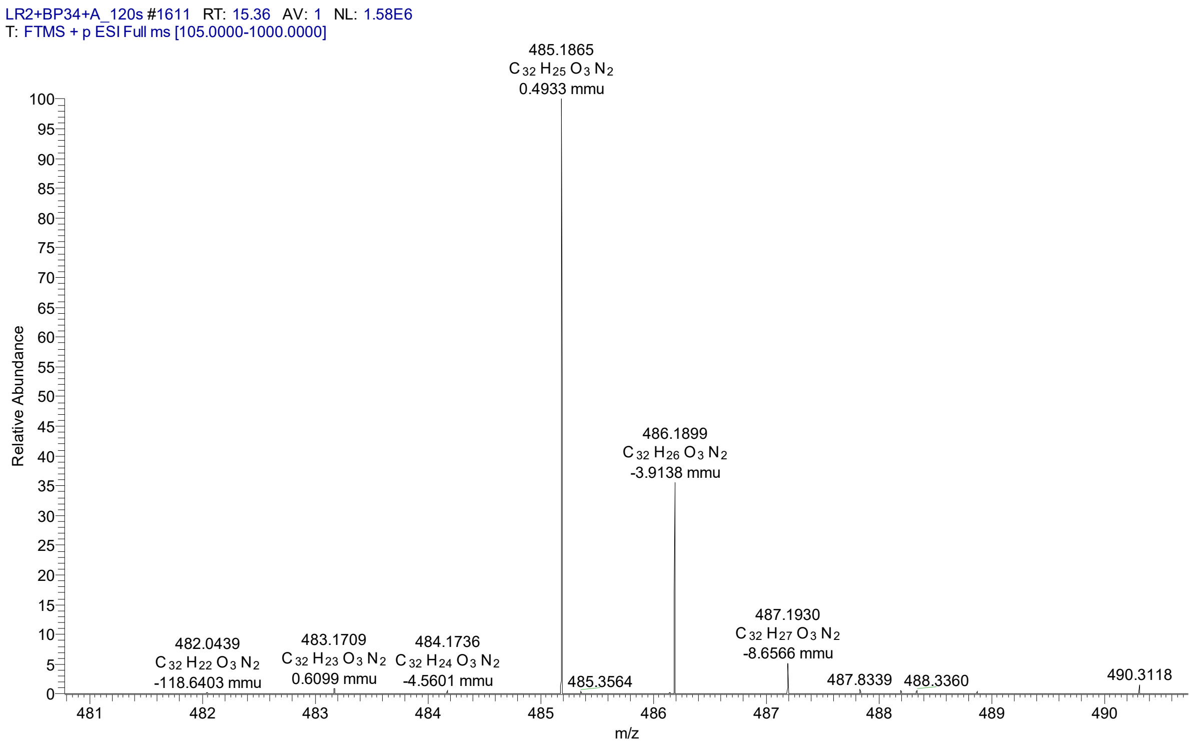

Figure S94: HR-MS (ESI) of product **16**; m/z calculated: $C_{32}H_{26}N_2O_3$ $[M]^+$: 486.19434; measured $[M-H]^+$: 485.1865.

1 with 11 (BCN)

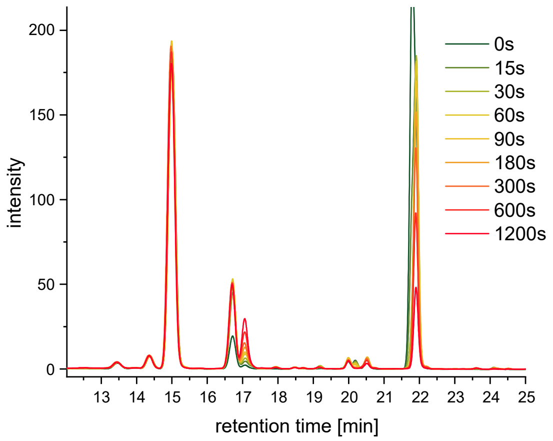

Figure S95: RP-HPLC spectra with 100 µM **1** and 1000 µM **11.**

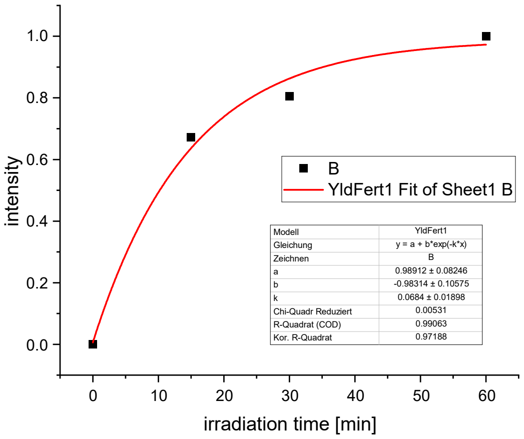

Figure S96: Second order rate constant calculation for **1+11** product formation.

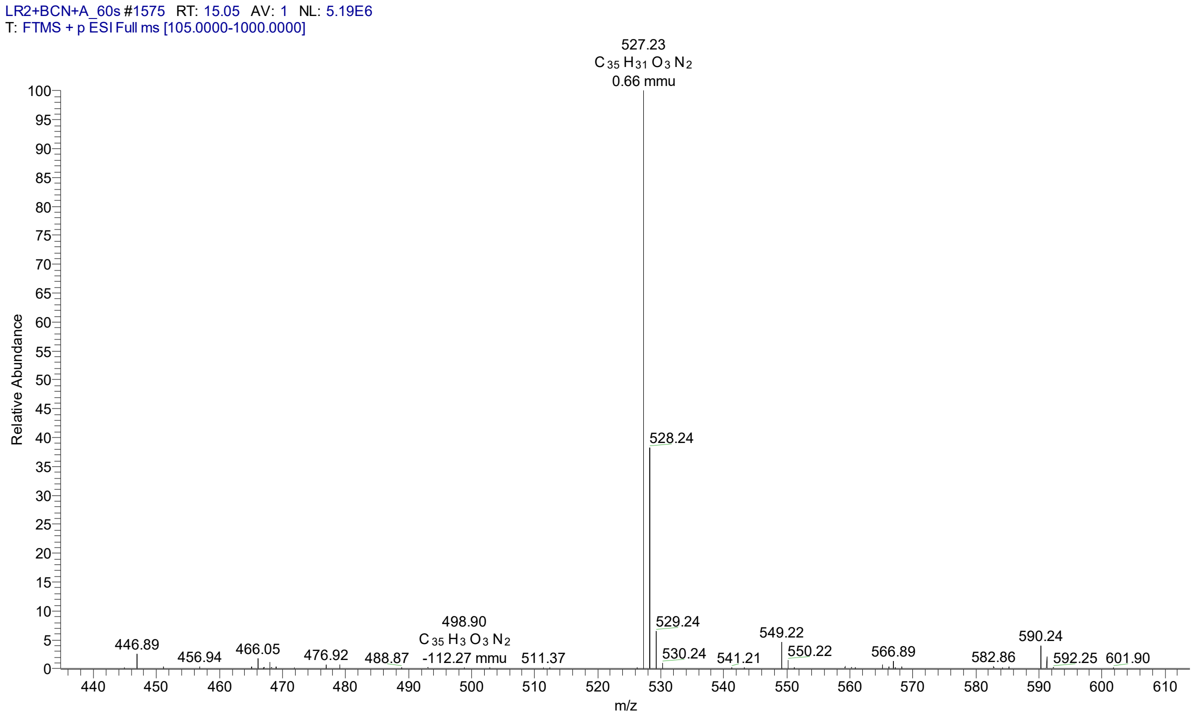

Figure S97: HR-MS (ESI) of product **17**; m/z calculated: $C_{35}H_{30}N_2O_3$ $[M]^+$: 526.22564; measured $[M+H]^+$: 527.23.

2 with 9 (MM)

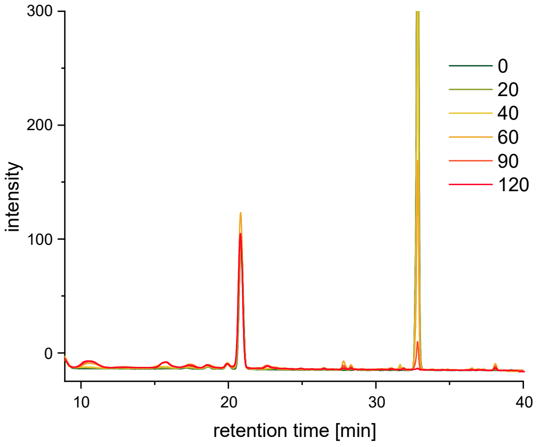

Figure S98: RP-HPLC spectra with 100 µM **2** and 1000 µM **9.**

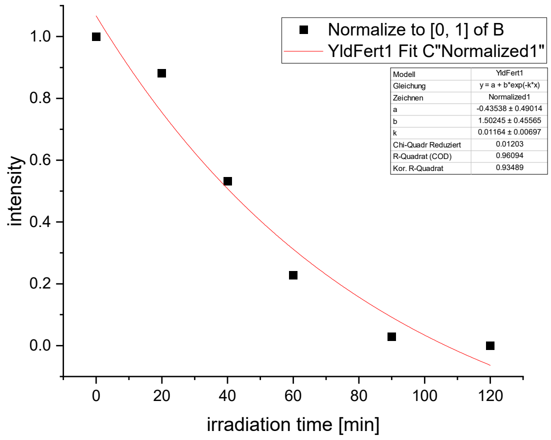

Figure S99: Second order rate constant calculation for **2+9**.

2 with 10 (spirocycle)

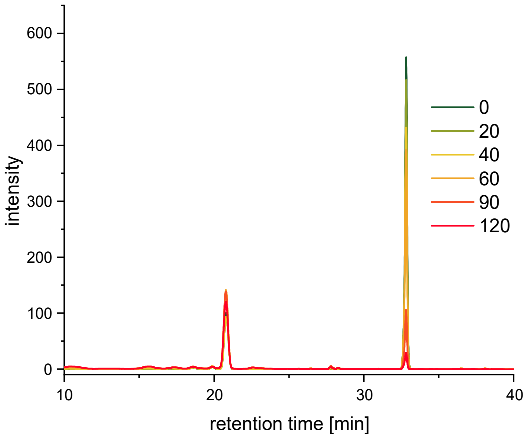

Figure S100: RP-HPLC spectra with 100 µM **2** and 1000 µM **10.**

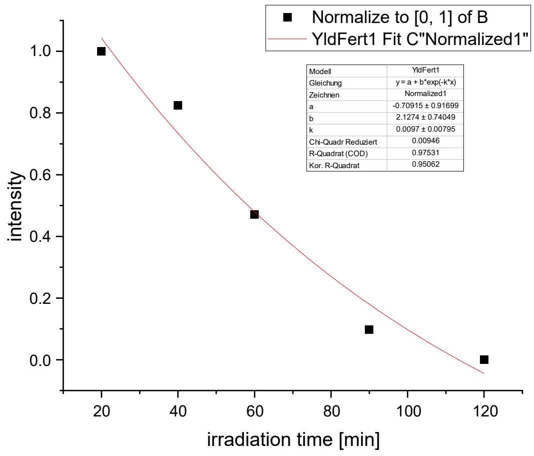

Figure S101: Second order rate constant calculation for **2+10**.

2 with 11 (BCN)

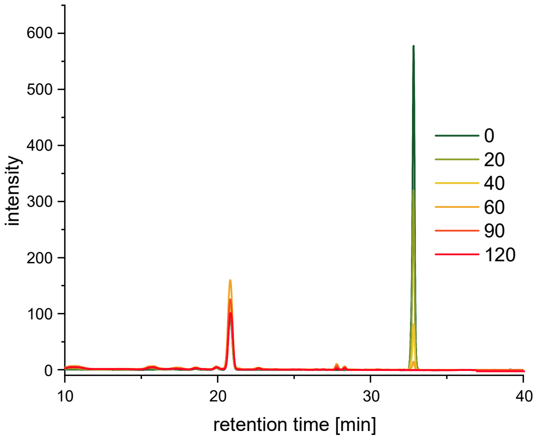

Figure S102: RP-HPLC spectra with 100 μM **2** and 1000 μM **11.**

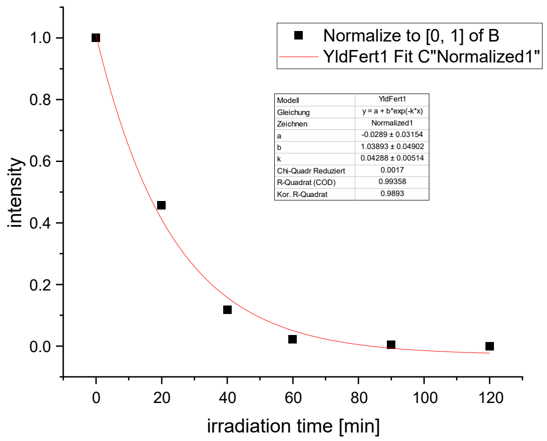

Figure S103: Second order rate constant calculation for **2+11**

f) Cell experiments

Human cervix carcinoma cells (HeLa, ATCC) were cultured under sterile conditions in Dulbecco's modified Eagle's medium ([+] 4.5g/L D-glucose, L-Glutamine, [+] Pyruvate, DMEM, Gibco^{TM}), supplemented with 10% fetal calf serum (HI FBS, Gibco^{TM}) and 1 U/mL Penicillin-Streptomycin (Gibco^{TM}) at 37 °C, 5% CO_2. For metabolic labelling experiments, $1 \cdot 10^4$ cells/well were transferred into an 8-well µ-slide (IBIDI, Ibitreat) and incubated with nucleoside **1** (20 µM, stock solution in DMSO) in DMEM with a final volume of 200 µM/well for 48 h. Cells were fixed with 4% paraformaldehyde in PBS for 10 min. Afterwards fixing was stopped with 50 mM glycine and 50 mM NH_4Cl in PBS for 5 min and washed twice with PBS. Hereupon, the cells were washed with 0.2% Trition-X 100 in PBS for 5 min. Afterwards cells were denaturated with 2 M HCl for 30 min at room temperature. Cells were then washed once with PBS and neutralized with 0.1 M Borax solution ($Na_2B_4O_7 \cdot 10H_2O$) for 10 min and twice washed with PBS. Then, APT (30 µM, stock solution in DMSO) or PT (60 µM, stock solution in DMSO) in MeCN was applied onto cells and incubated for 1 h at room temperature. Accordingly, the IBIDI was photoirradiated with a 405 or 450 nm LED for 30 min at 25°C and afterwards washed with MeCN three times if not described otherwise. After irradiating, the fixed cells were imaged *via* confocal fluorescence microscopy using a Leica DMI8, TCS SP8 inverse microscope with a HC PL APO 63x/1.40 OIL CS2 objective. Image acquisition was conducted at a lateral resolution of 2760 x 2760 pixels and 8 bit depth using the LAS-AF 1.1.0.12420 software. **1** and **2** were excited using the 405 nm laser (10% intensity) and fluorescence emission was measured at 420-500 nm, complemented with a brightfield image.

1 (60 µM) with DRAQ5 (20 µM) in MeCN and PBS

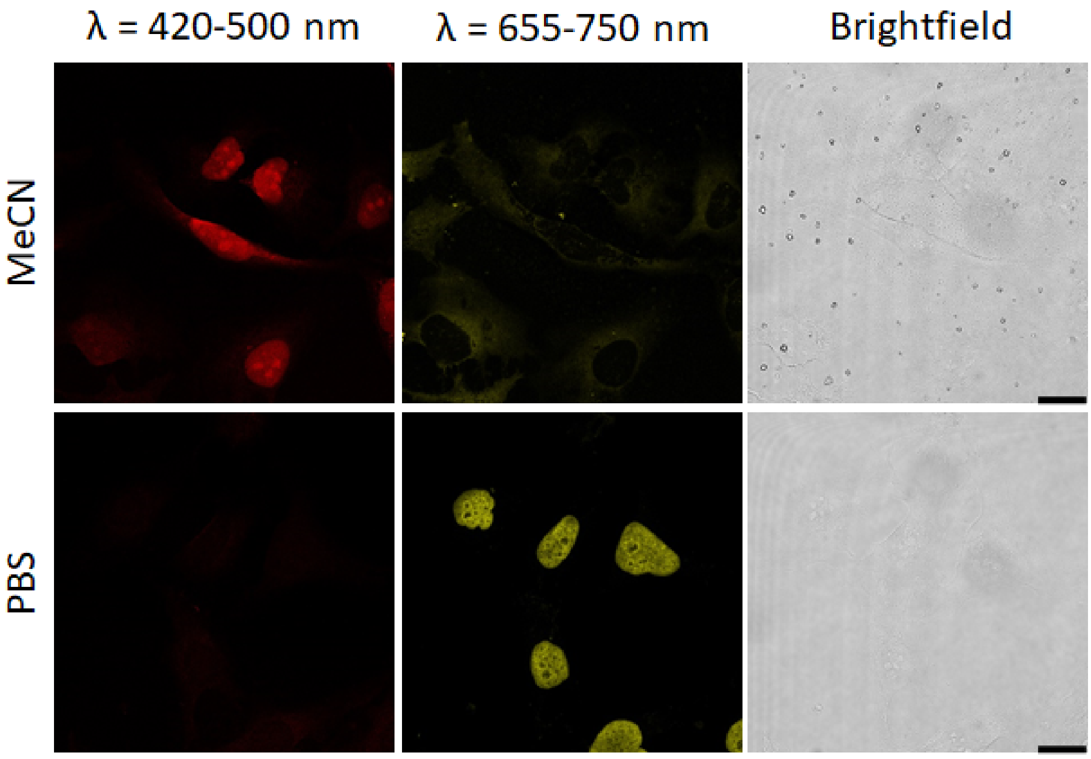

Figure S104: Control staining experiments with DRAQ5 (20 µM) that stains nuclear DNA in PBS and **1** (60 µM) staining the nuclear DNA in MeCN.

2 (30 µM) with DRAQ5 (20 µM) in MeCN and PBS

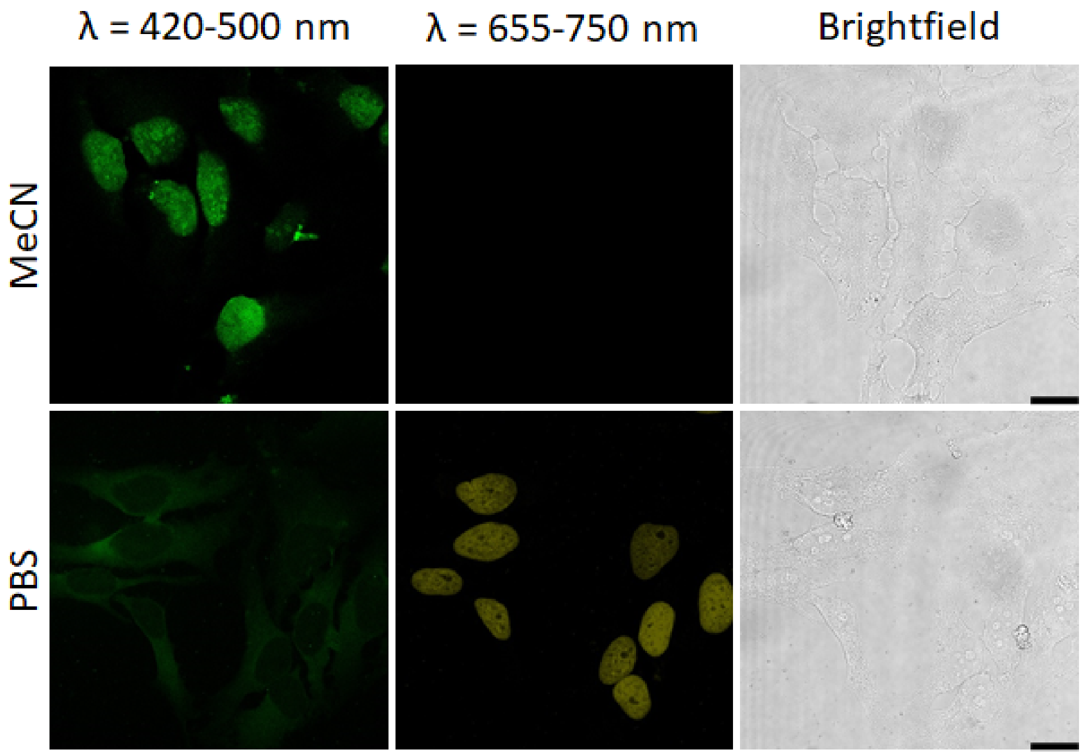

Figure S105: Control staining experiments with DRAQ5 (20 µM) that stains nuclear DNA in PBS and **2** (30 µM) staining the nuclear DNA in MeCN

1 (60 µM) with VdU (20 µM) in MeCN

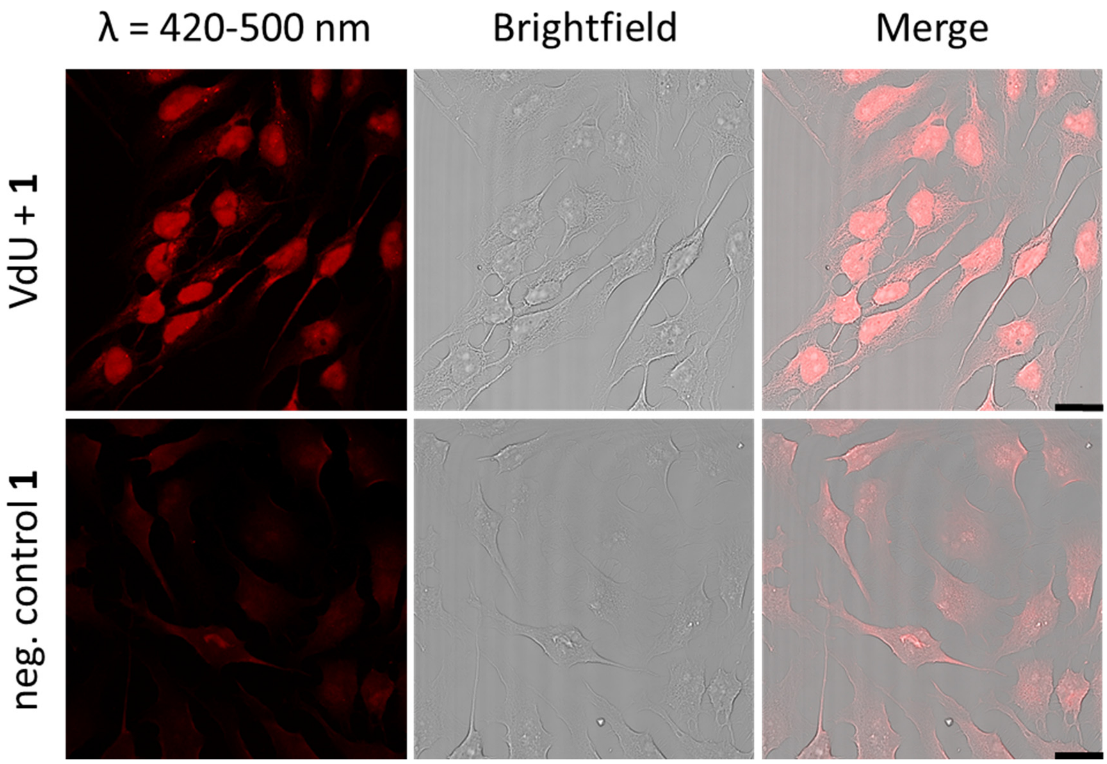

Figure S106: Labelling experiment with **VdU** (20 µM) and **1** (60 µM). Irradiation with 405 nm for 90s. After labelling step and irradiation only one washing step with MeCN was perfomed before confocal microscopy. Negative control includes **1** (60µM) but no **VdU** (20 µM) incubation step before fixation of the cells.

g) References

[1] P. W. Kamm, J. P. Blinco, A. N. Unterreiner, C. Barner-Kowollik, *Chem. Commun.* **2021**, *57*, 3991-3994.
[2] L. Sun, J. Ding, W. Xing, Y. Gai, J. Sheng, D. Zeng, *Bioconjugate Chem* **2016**, *27*, 1200-1204.
[3] A. S. Hashmi, F. Rominger, *Chem. Eur. J.* **2013**, *19*, 382-389.
[4] D. H. Hilko, L. F. Bornaghi, S. A. Poulsen, *J. Org. Chem.* **2018**, *83*, 11944-11955.
[5] Z. Yu, Q. Lin, *J. Am. Chem. Soc.* **2014**, *136*, 4153-4156.
